# How race affects evidence accumulation during the decision to shoot

**DOI:** 10.3758/s13423-017-1369-6

**Published:** 2017-10-05

**Authors:** Timothy J. Pleskac, Joseph Cesario, David J. Johnson

**Affiliations:** 1 Center for Adaptive Rationality, Max Planck Institute for Human Development, Lentzeallee 94, 14195 Berlin, Germany; 2 Psychology Building, Michigan State University, 316 Physics Road, Room 255, East Lansing, MI 48824 USA

**Keywords:** Race bias, First person shooter task, Sequential sampling, Signal detection, Diffusion model

## Abstract

The biasing role of stereotypes is a central theme in social cognition research. For example, to understand the role of race in police officers’ decisions to shoot, participants have been shown images of Black and White males and instructed to shoot only if the target is holding a gun. Findings show that Black targets are shot more frequently and more quickly than Whites. The decision to shoot has typically been modeled and understood as a signal detection process in which a sample of information is compared against a criterion, with the criterion set for Black targets being lower. We take a different approach, modeling the decision to shoot as a dynamic process in which evidence is accumulated over time until a threshold is reached. The model accounts for both the choice and response time data for both correct and incorrect decisions using a single set of parameters. Across four studies, this dynamic perspective revealed that the target’s race did not create an initial bias to shoot Black targets. Instead, race impacted the rate of evidence accumulation with evidence accumulating faster to shoot for Black targets. Some participants also tended to be more cautious with Black targets, setting higher decision thresholds. Besides providing a more cohesive and richer account of the decision to shoot or not, the dynamic model suggests interventions that may address the use of race information in decisions to shoot and a means to measure their effectiveness.

**Electronic supplementary material:**

The online version of this article (10.3758/s13423-017-1369-6) contains supplementary material, which is available to authorized users.

There is no shortage of reports of unarmed Black citizens in the United States being shot by police officers (America’s police on trial, 2014; Cobb, 2016; Don’t shoot, 2014; The counted: People killed by police in the US, 2016). These shootings have raised the questions of whether and how racial stereotypes might impact officers’ split-second decisions to shoot.^1^ Clearly, police officers deciding whether or not to use deadly force are in an uncertain and high-pressure situation, especially when the target person is holding an object in need of rapid identification. It is in the face of such uncertainty that stereotypes can impact behavior by providing information—traits and behaviors associated with the social category (Higgins, 1996; Tajfel, 1969)—that seems to disambiguate the situation. For example, classic work in social psychology has shown that people rate an ambiguous shove as more violent when performed by a Black than a White individual (Duncan, 1976; Sagar & Schofield, 1980).

In the context of shooting decisions, the challenge has been to understand not only whether stereotypes impact the decision to shoot, but how they enter the process. To begin to answer these questions, simplified computer-based analogues of the decision situation have been constructed: A target individual appears on a computer screen and participants must decide whether or not to shoot the target (Correll, Park, Judd, & Wittenbrink, 2002). Mathematical models of the decision process are then applied to the choice data to determine how race impacts the decision process. The model most commonly used to understand the decision to shoot is based on signal detection theory (SDT; Green & Swets, 1966; Macmillan & Creelman, 2005). According to SDT, individuals take a sample of information from the scene and decide to shoot if and only if the strength of the sample exceeds a criterion level of strength. Modeling the decision in this way has indicated that the criterion used for Black targets is lower than that applied for White targets (Correll et al., 2002; Correll, Park, Judd, & Wittenbrink, 2007a).

A great limitation of SDT is that it treats the decision to shoot as a static decision process. That is, it assumes that all the information used to make a decision is extracted from the scene in a single sample. Static approaches often provide a reasonable approximation of the decision process and certainly capture some psychologically important aspects of the decision. In this article, however, we take a different approach and model the decision to shoot as a dynamic process in which information is accumulated as evidence over time until a decision threshold is reached (Edwards, 1965; Laming, 1968; Link & Heath, 1975; Ratcliff, 1978; Stone, 1960).

Moving to dynamic models has important consequences for understanding how stereotypes impact the decision to shoot. One consequence is that the models quantitatively predict both choice and response times, whereas static models predict choices only. A second consequence is that it can provide a more nuanced understanding of how race and other factors impact the different components of the decision process. As we show below, both of these advantages are important because (1) race in some conditions only has a statistically reliable impact on response times and not the observed choices, and (2) race may have multiple, even antagonistic effects on different decision components. Both of these features are difficult for traditional static decision models to handle.

The structure of this article is as follows. We first review the first-person shooter task (FPST; Correll et al., 2002), a task used to study how race impacts the decision to use deadly force. We then describe the drift diffusion model (DDM), the dynamic decision model that we used to model the decision process. We use the model to develop a set of hypotheses and questions about how race might impact the decision process. We next test those hypotheses on four FPST datasets and present results that speak to the validity of the model to meaningfully measure properties of the decision process. Finally, we integrate the data across the four common conditions of the studies to provide an overall summary of the effect of race on the decision process. Taken together, the DDM reveals a multifaceted effect of race on decision making that is stable at the cognitive level across datasets, regardless of the study conditions.

On a methodological note, an important aspect of these four datasets is that they are typical of studies in the published literature, with the observed race bias being more pronounced in response times (Study 1), in error rates (Study 2 and Study 4), or weakly so in both (Study 3). They are also typical in that the designs are close to those used in experimental social psychology, where many subjects complete a small number of trials over many conditions. This type of design presents a unique challenge; fitting dynamic decision models like the DDM typically requires experimental designs in which a few subjects complete many trials over a small number of conditions (often more than 2,000 trials per subject per condition; e.g., Ratcliff & Smith, 2004). We solved this issue by embedding our models within a Bayesian hierarchical framework (Vandekerckhove Tuerlinckx, & Lee, 2011; Wabersich & Vandekerckhove, 2014). The hierarchical framework allows data from one subject to inform their own parameter estimates in different conditions as well as the parameter estimates of other subjects in the same conditions. It thus enabled us to acquire reliable and accurate estimates of the parameters of the decision process. Another advantage of this approach is that it facilitates the integration of data across studies, allowing us to synthesize the evidence for the overall effect of race on the decision process and to analyze how the effect of race on the decision process changed or did not change across studies.

We should note that there have been some applications using the DDM to model the decision process in studies of social cognition (Benton & Skinner, 2015; Klauer & Voss, 2008; Klauer, Voss, Schmitz, & Teige-Mocigemba, 2007; van Ravenzwaaij, van der Maas, & Wagenmakers, 2010; Voss, Rothermund, Gast, & Wentura, 2013), including one report modeling how race impacts the decision to shoot that was published as we worked on this project (Correll, Wittenbrink, Crawford, & Sadler, 2015). Our work builds on these studies, but also goes beyond them in at least three ways. First, the previous studies largely used conventional methods to fit models at the individual level only (though see Krypotos, Beckers, Kindt, & Wagenmakers, 2015). To this end, they either simplified their experimental designs to focus on a single manipulation or simplified the model and examined how a reduced set of process parameters were impacted by race. The Bayesian hierarchical approach allowed us much more flexibility to examine how race impacts many more aspects of the decision process. Second, we used the model to examine how other key factors (e.g., context and response window) might moderate the effect of race or even impact the decision process directly. Third, our Bayesian hierarchical approach offers a solution for estimating the parameters and uncertainty in these parameters at both the individual and the group level. This approach, we contend, is useful not only for gaining a better understanding of the psychology behind decisions to shoot, but also for other questions in social cognition and social psychology where response time and decision data are obtained for a single task across many trials.

## First-person shooter task

Psychologists studying how stereotypes influence the use of deadly force have developed laboratory analogues of this decision, the most common of which is the FPST (Correll et al., 2002). Participants in the FPST view a series of neighborhood images on a computer screen. After a short period of time a target individual appears holding an object. Participants are instructed to press a button labeled “Shoot” if the target is holding a gun and a button labeled “Don’t Shoot” if the target is holding a harmless object (e.g., phone, wallet).

The FPST and similar tasks have been used in countless investigations of the role of race in the decision to shoot. The task has revealed a robust race bias in the decision among undergraduate participants and community samples (e.g., Correll et al., 2002; James, Klinger, & Vila, 2014; Plant, Peruche, & Butz, 2005). In some conditions, particularly when participants face a response deadline of 630 ms, the bias appears more reliably in error rates: Participants are more likely to shoot unarmed Black targets than unarmed White targets (e.g., Correll et al., 2002; Correll, Park, Judd, & Wittenbrink, 2007a; Correll, Park, Judd, Wittenbrink, Sadler, & Keesee, 2007b). When the response window is increased from 630 ms to 850 ms, the observed race bias tends to shift to response times: Participants are faster to shoot armed Black targets and slower to not shoot unarmed Black targets (Correll et al., 2002; Greenwald, Oakes, & Hoffman, 2003; Plant & Peruche, 2005; Plant et al., 2005). This form of bias also tends to be observed in trained police officers (Correll, Park, Judd, Wittenbrink, Sadler, & Keesee, 2007b; Sim, Correll, & Sadler, 2013) and people more familiar with the task (Correll et al., 2007a).

## Modeling the decision to shoot

To go beyond the behavioral data and better understand the race bias at the cognitive level, researchers have employed mathematical models to analyze the decision process in the FPST. The most common approach is to treat the decision as a signal detection process using SDT (Green & Swets, 1966; Macmillan & Creelman, 2005). From this perspective, on each trial, the shooter extracts a sample of information reflecting the degree to which the target appears to be holding a gun. The shooter then compares the strength of that information against a criterion to detect whether a gun (i.e., a signal) is present (Correll et al., 2002, 2007, 2011). When the choice data are subjected to this approach, race affects the decision criterion, with participants setting a lower criterion for Black targets than for White targets, reflecting a bias in their response process.^2^

A limitation of SDT as a model of the decision process is that it is silent in terms of response times. This is problematic when it comes to explaining differences in race effects observed between experiments. Recall that race primarily affects the observed error rates in some cases, but the speed of correct responses in others (a pattern we replicate in our data). Why is extending the response window from 630 to 850 ms enough to induce race-based differences in response times while suppressing any differences in the observed decisions? Conversely, why should reducing the response window to 630 ms be enough to significantly increase the probability of incorrectly shooting unarmed Black targets, while simultaneously suppressing race-based differences in response time? And why focus solely on response times for correct choices and not also incorrect responses? Finally, what should one conclude when the race bias is present in response times but not error rates as is the case, for instance, in some instances when police officers complete the task (Correll et al., 2007; Sim et al., 2013)? While an SDT approach cannot answer these questions, as we show below the DDM is able to do so.

## Drift diffusion model of the first-person shooter task

**Fig. 1:**
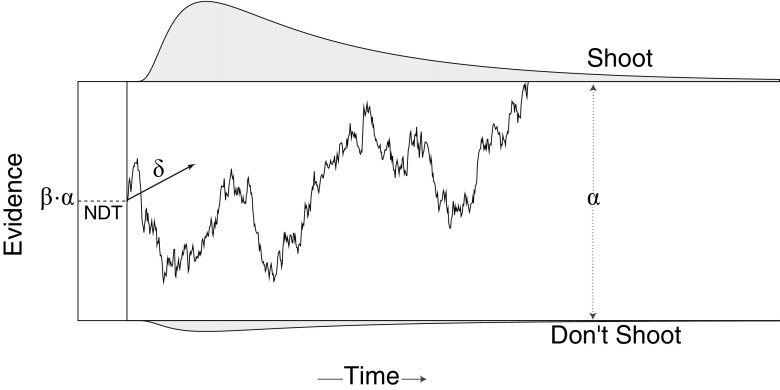
A realization of a drift diffusion process during the first-person shooter task. According to the model, participants deciding whether or not to shoot sequentially accumulate evidence over time. The jagged line depicts the path the evidence takes on a hypothetical trial. The distributions at the top and bottom illustrate the predicted distribution of times for the given set of process parameters at which the evidence reaches each threshold. The relative area under each distribution is the predicted proportion of trials in which participants will choose each response

The DDM describes decision making as a dynamic process that unfolds over time predicting both choice and response time. A realization of this process is shown in Fig. 1. According to the DDM, the decision to shoot or not is based on an internal level of evidence. At the onset of the trial, this evidence can have an initial bias towards either option. Over time, participants extract further information from the scene on whether or not to shoot, which gives rise to an evolving (latent) level of evidence depicted by the jagged line in Fig. 1. The jaggedness arises because each sample of evidence is noisy (i.e., the scene itself and the cognitive and neural processes used to extract evidence introduce variability into the evidence). Once a threshold level of evidence has been reached, a decision is made: the “Shoot” option is selected if the accumulated evidence reaches the upper threshold, the “Don’t Shoot” option if it crosses the lower threshold. The time it takes for the evidence to reach either threshold is the predicted decision time, *t*_*D*_.

The DDM decomposes the observed distribution of choices and response times into four psychologically meaningful parameters. Descriptions of these four main DDM parameters and their substantive interpretations are given in Table 1. Estimates of the parameters are obtained by fitting the DDM directly to the observed distributions of choices and response times. This can be done because, as stated earlier, the DDM predicts the probability of choosing to shoot or not shoot and the distribution of possible response times for a given set of parameters for each trial (Fig. 1).

**Table 1:** Four main parameters of the drift diffusion model and their substantive interpretations

Drift Diffusion Model Parameter	Description
Drift rate (*δ*)	The average strength in evidence at each unit of time, with $-\infty < \delta < \infty $. The sign of the drift rate indicates the average direction of the incoming evidence, with negative values indicating evidence in favor of “Don’t Shoot” and positive values indicating evidence in favor of “Shoot.” The magnitude of the drift rate characterizes the quality of the incoming information.
Threshold separation (*α*)	The separation between the thresholds, with 0 < *α*. With this parameterization, the choice threshold for the uncertain option is set at *α*, and the choice threshold for the certain option set at 0. The threshold separation determines how much a person trades accuracy for speed (i.e., the speed–accuracy tradeoff), with larger values indicating more accurate but slower decisions.
Relative start point (*β*)	The location of the starting point for evidence accumulation relative to the thresholds, with 0 < *β* < 1. With this parameterization, the start point *z* is *z* = *β* ⋅ *α*. The relative start point indexes an initial bias for either response, with values of *β* greater than .5 indicating a bias to choose “Shoot” and values lower than .5 indicating a bias to not shoot.
Non-decision time (*N* *D* *T*)	The amount of contaminant time in the observed response times beyond the deliberation time specified by the DDM, with 0 < *N* *D* *T*. The non-decision time includes the time spent on encoding the stimulus, executing a response, and any other contaminant process.

The drift rate *δ* describes the average strength of evidence in each sample.^3^ A positive drift rate indicates evidence on average pointing to the presence of a gun. A negative drift rate indicates evidence on average pointing to the presence of a non-gun object. The magnitude of the drift rate in either direction characterizes the strength of the evidence for each option.

The drift rate has similar properties to measures of sensitivity such as *d* ^′^ in SDT (Green & Swets, 1966; Macmillan & Creelman, 2005). One difference is that *δ* can be conceptualized as a measure of sensitivity per unit of time whereas *d* ^′^ represents sensitivity across time and thus confounds accuracy with processing time (Busemeyer & Diederich 2010). Another difference is that the DDM can estimate separate drift rates for gun and non-gun objects, whereas *d* ^′^ is a single value representing the difference in sensitivity between the two classes of objects. As we will see, the ability of the DDM to separately measure the quality of information for gun and non-gun objects provides new insights into how race affects the decision process.^4^

The separation *α* between the two thresholds describes the amount of evidence required to make a decision, with larger values indicating greater amounts of information. Decreasing the threshold separation *α* reduces the amount of evidence needed for a choice, which in turn reduces the amount of time a person takes to make the decision and also increases the chances of an error (due to the variability in evidence). Thus, the threshold separation *α* reflects the extent to which a person trades accuracy for speed. This is the mechanism that helps explain how different response windows in the FPST lead to race bias being present in either error rates or response times.

An important aspect of the DDM is that it can also capture an initial bias in the decision to shoot. This bias is characterized by the parameter *β*, which is the location of the starting point of evidence accumulation relative to the total threshold separation. When *β* = .5 there is no bias; biases toward shooting have values closer to 1; and biases toward not shooting have values closer to 0.

Finally, the non-decision time parameter *N* *D* *T* measures contaminants to response times beyond the deliberation time specified by the DDM (see dashed line in Fig. 1). These contaminants include pre- and post-decision deliberation (e.g., encoding vs. motor time) as well as any other process that adds to the response. In practice, it is not usually possible to identify these different contaminants. Thus, the observed response time *t* is an additive combination of a single non-decision time and the predicted decision time from the model, *t* = *t*_*d*_ + *N* *D* *T*.

For a given relative starting point *β*, threshold separation *α*, drift rate *δ*, and non-decision time *N* *D* *T*, the model predicts the probability of a “Shoot” or “Don’t Shoot” decision, as well as the response time distributions for each decision. Expressions and derivations for these functions can be found elsewhere (Busemeyer & Diederich 2010; Cox & Miller 1965; Voss & Voss 2008). More complex models capturing other important aspects of the decision process exist, such as versions including trial-by-trial variability in parameters to account for slow and fast errors (Ratcliff, 1978; Ratcliff & Rouder, 1998; Ratcliff, Van Zandt, & McKoon, 1999), changes in information processing as attention switches between attributes or sources of information (Diederich, 1997; Diederich & Busemeyer, 2015), extra processing stages to account for confidence (Pleskac & Busemeyer, 2010), decay parameters to account for memory decay or the leakage of evidence (Busemeyer & Townsend, 1993; Yu, Pleskac, & Zeigenfuse, 2015), linkage functions to account for neural data (Turner, van Maanen, & Forstmann, 2015), or ways to model choices with more than two alternatives (Diederich & Busemeyer, 2003, Krajbich & Rangel, 2011) or even continuous ratings (Kvam, 2017; Smith, 2016). We have explored some of these more complex models such as models with trial-by-trial variability in the parameters. However, the experimental designs of most studies do not permit accurate estimates of these aspects. For this reason, we focus here on the simpler version of the model, investigating how race and other aspects of the decision scenario impact the four core cognitive parameters specified during the FPST decision process. We believe the theoretical framework we develop here is an important foundation for gaining a better understanding of the decision to shoot and opens the door to future work to build a more complete processing model of the decision.

We should also mention that the DDM is one of many different dynamic decision models that assume a sequential sampling process. In general, these models can be divided into accumulator models and random walk/drift diffusion models (Ratcliff & Smith, 2004; Townsend & Ashby, 1983). Accumulator models accumulate evidence separately for each response alternative, allowing the evidence for one alternative to be independent of the evidence for the other (e.g., Audley & Pike, 1965; Brown & Heathcote, 2008; LaBerge, 1962; Townsend & Ashby, 1983; Usher & McClelland, 2001). Random walk/drift diffusion models, in contrast, accumulate evidence dependently for each response alternative, such that evidence for one alternative is evidence against the other (e.g., Edwards, 1965; Laming, 1968; Link & Heath, 1975; Ratcliff, 1978).^5^ The two model types often make very similar predictions; for our purposes, they typically differ only in the quantitative details of the predictions (Ratcliff & Smith, 2004). In this article, we rely on the DDM to test our general hypothesis that the decision to shoot is best modeled as a dynamic decision process. We focus on the DDM for two reasons. First, to date it is arguably the most successful approach for capturing the dynamic process of evidence accumulation (e.g., Bogacz, Brown, Moehlis, Holmes, & Cohen, 2006; Busemeyer & Townsend, 1993, 2007; Krajbich & Rangel, 2011; Nosofsky & Palmeri, 1997; Pleskac & Busemeyer, 2010; Ratcliff, 1978; Ratcliff & Smith, 2015; Voss et al., 2004; Voss et al., 2007). Second, as we have mentioned and will discuss shortly, in order to model the data we need Bayesian hierarchical instantiations of the models, which are currently available for the DDM (Vandekerckhove et al., 2011; Wiecki, Sofer, & Frank, 2013) (though, for very recent accumulator model implementations, see Annis, Miller, & Palmeri, 2016; Turner, Sederberg, Brown, & Steyvers, 2013).

## Hypotheses on the effects of race on the decision process

According to the DDM, there are different mechanisms by which race can impact the decision to shoot. However, within the framework of the model, there are only two plausible hypotheses by which race can lead to an asymmetric change in error rates and faster “Shoot” decisions for armed Black targets and slower “Don’t Shoot” decisions for unarmed Black targets (Correll et al., 2015; Klauer, Dittrich, Scholtes, & Voss, 2015).

### Start point hypothesis

One mechanism is through the relative start point *β*, with participants setting a starting point closer to the shoot threshold for Black targets than for White targets. This shift in the relative start point thus captures what is meant by the term “trigger happy.” One issue of note here is that, in any given FPST trial, participants do not know the target’s race until the target appears holding the object. Thus, to entertain this hypothesis, we would need to assume that the race of the target individual is the first piece of information that is processed (before any accumulation of gun/non-gun evidence).

### Evidence hypothesis

A second hypothesis is that the evidence participants extract from the scene depends not only on the object, but also on the target. That is, participants process both the target and the object as evidence in determining whether to shoot or not. Thus, the degree to which the evidence from guns points towards “Shoot” and the evidence from non-gun objects points towards “Don’t Shoot” also depends on the race of the target. This hypothesis suggests two possible effects of race on drift rate *δ*, one for guns and one for non-gun objects.

The first effect is that the drift rate for armed Black targets could be stronger (evidence accumulates more quickly) than that for armed White targets: When a Black target is armed, the evidence for “Shoot” is stronger than when a White target is armed. Consequently, armed Black targets are more likely to be shot than armed White targets and on average will be shot more quickly. Therefore, changes to the drift rate for guns would account for both decreased misses and faster correct “Shoot” decisions for Black targets.

The second effect is that the drift rate for unarmed Black targets could be weaker (evidence accumulates more slowly) than that for unarmed White targets: When a Black target is unarmed, the evidence for “Don’t Shoot” is weaker than when a White target is unarmed. Consequently, unarmed Black targets are more likely to be incorrectly shot than unarmed White targets and the decision not to shoot will be registered more slowly for Black than for White targets. Therefore, changes to the drift rate for non-guns would account for both increased false alarms and slower correct “Don’t Shoot” decisions for Black targets.

Thus, a race effect on the drift rate for the gun objects, the non-gun objects, or both, can explain both response time and error rate differences for Black and White targets in the FPST with reference to a single set of parameter changes. Either combination is sufficient to produce an interaction between race and object type in error rates or response times (i.e., race bias). Indeed, at the behavioral level, the reported interaction is sometimes due to race reliably impacting unarmed targets (Plant & Peruche, 2005), armed targets (Study 2 in Correll et al., 2002), or both (Correll, Wittenbrink, Park, Judd, & Goyle, 2011). The DDM enables us to better measure which target shows more of a race effect and why, with important consequences for both predicting and correcting race bias.

### Threshold-separation question

The DDM also raises a number of new empirical questions about the decision process during the FPST. One question is whether the race of the target impacts the quantity of evidence accumulated, i.e., threshold separation *α*. Given that the race of the target and the object become apparent simultaneously, it is possible that race has no effect on *α*. However, perhaps due to increased anxiety or sense of urgency, participants may simply rush to make a decision—any decision—when they see a Black target and thus reduce the threshold separation *α* for Black targets (see, for example, Thura, Cos, Trung, & Cisek, 2014). An alternative possibility is that participants increase the threshold separation *α* for Black targets, perhaps as a means to control their possible stereotype biases (i.e., a motivation to control prejudice; Plant & Devine, 1998). Note just as with the start-point hypothesis, these possible effects on threshold separation do necessitate that some pre-processing of target race must occur.

### Context question

A second question pertains to the moderating effect of context on the race bias. Correll et al. (2011) reported that the race bias is eliminated when targets appear in dangerous neighborhood backgrounds in the FPST. According to SDT, this is because participants lower their criterion for dangerous contexts, which in turn washes out the effect of race on the criterion. In Studies 2, 3, and 4, we investigated how changes in context impact the decision process when the DDM is employed.

### Discriminability question

Finally, we asked how reducing the discriminability of the object (i.e., blurring the image of the gun or other object) changes the decision process. This question actually gets at the properties of the evidence gleaned from objects during the decision to shoot. To see how, consider the decision from the perspective of a signal detection process. From this perspective, the gun is the signal. Blurring the gun object should reduce the average strength of the signal (the strength of the information extracted from the gun object). Now consider what might happen with non-gun objects. If non-gun objects provide no signal (i.e., are just noise), then blurring them should have no effect on the information extracted. However, if non-gun objects also carry some signal (e.g., either by bearing a resemblance to a gun or carrying some information of danger), then blurring them should also reduce the strength of information extracted from non-gun objects. If this is the case, the SDT model will characterize the effect of blur not as a change in discriminability, but as a change in the criterion. This is because discriminability in the SDT model is the difference between the strength of the signal for armed and unarmed targets, and the model assumes that the average signal inferred from the non-gun trials is fixed at 0 (i.e., just noise). The DDM, however, can measure the strength of the evidence for armed and unarmed targets separately and thus can accurately isolate the effect of blur to the strength of the evidence being accumulated (i.e., drift rates).

## General methods

### Experimental methods

We tested the DDM using four separate and previously unpublished datasets. Studies 1 and 2 were unpublished data collected by another lab from undergraduates recruited from psychology subject pools at the University of Chicago.^6^ In Study 1, participants (*N* = 56 self-identified Caucasians) completed 100 trials of a FPST in which the target appeared holding either a gun or a non-gun object. Race of the target was manipulated between trials, and all targets appeared in front of neutral neighborhood scenes (the standard scenes used in the FPST, e.g., parks, city sidewalks). In Study 2, participants (*N* = 116 self-identified Caucasians) completed 80 trials of a FPST which manipulated the race of the target individual, the object held by the target (both within-subjects), and the dangerousness of the context in which targets were presented (between-subjects). Targets were presented in either the standard neutral scenes or urban scenes meant to convey danger, including images of dilapidated buildings, dumpsters, subway terminals with graffiti, etc. (from Correll et al., 2011).

We designed and collected the data for Studies 3 and 4 recruiting participants from the psychology department subject pool at Michigan State University. In Study 3, we sought to replicate the results ourselves. We asked participants (*N* = 38 self-identified Caucasians) to complete a larger number of trials (320) of a FPST that manipulated within-subjects the race of the target individual, the object held by the target, and the context (neighborhood) in which targets were presented. We also manipulated the discriminability of the target to better understand the nature of the information being accumulated during the decision process. The results of Study 3 were, in general, consistent with those of Studies 1 and 2, but the DDM analysis isolated the effect of race to be on the non-gun objects rather than the gun objects. Therefore, we ran a fourth study with a larger sample size. In this final study, participants (*N* = 108 self-identified Caucasians) completed 320 trials of the FPST that again manipulated the race of the target individual, the object held, and the context (neighborhood).

The basic FPST method was consistent across all four studies. We do not have the precise experimental set up for Studies 1 and 2. In Studies 3 and 4, participants completed the task in PsychoPy (1.80.06) on an 20 inch (16.96 by 10.60 inch) iMac computer running OS X (10.6.8). The stimuli were presented so that they filled the screen without stretching (14.13 inch by 10.60 inch). In study 3 participants sat approximately 12 inches from the monitor. In Study 4 we manipulated distance from the screen with participants resting their heads in a chinrest either 12 inches or 24 inches away from the computer screen.

On each trial, one of four background scenes appeared for a fixed duration each. The duration was chosen at random from one of three possible durations (e.g., 500, 750, or 1000ms).^7^ After these background scenes, a target individual was shown holding either a handgun or a non-gun object (e.g., wallet, cell phone, camera). Participants were instructed to press a button labeled “Shoot” if the target individual was armed with a handgun and a button labeled “Don’t Shoot” if he was holding any other object. The target individuals were 20 young to middle-aged adult men; half were Black and half were White. Each individual was presented four times, twice with a handgun and twice with a non-gun object. These 80 individuals appeared in random locations within the backgrounds. Participants first completed a set of practice trials (typically 16) before moving to the experimental trials.

Participants were instructed to respond as quickly as possible, with the response window set at 850ms (Study 1), 630ms (Study 2 and Study 4), or 750ms (Study 3). As is the convention in the FPST task, participants earned points for their performance, and the point structure was designed to bias participants to shoot and reflect to some degree the payoff matrix officers face in the decision to shoot (Correll et al., 2002). A hit (correctly shooting an armed target) earned 10 points and a correct rejection (not shooting an unarmed target) earned 5 points. A false alarm (shooting an unarmed target) was punished by a loss of 20 points, and a miss (not shooting an armed target) led to the deduction of 40 points. If participants responded outside the window, points were deducted and they were told that their response was too slow.

### Behavioral analysis

Although our focus is on how race impacts decisions at the process level, we also report the effects of race at the behavioral level. To do so, we followed convention in the literature and submitted the error rates and correct response times from each study to an analysis of variance. The Supplemental Material provides the full ANOVA tables for all behavioral-level analyses. As the studies were designed within the framework of Null Hypothesis Testing, we rely on p-values and estimates of effect sizes for the substantitive conclusions from the behavioral level analyses. However, we also report Bayes factors for each effect as a means of informing the interpretation and the degree of confidence one can have in the specific conclusion.

Inclusion Bayes factors provide an estimate of the evidence for a particular effect combined across all the possible ANOVA models containing the effect (Rouder et al., 2016). The Bayes factors were estimated using JASP (JASP Team, 2017; Morey & Rouder, 2015). The Bayes factors are provided in terms of the evidence in favor of the alternative hypothesis, thus we use the notation *B* *F*_10_. Conventionally, Bayes factors between 1 and 3 are understood as indicating weak evidence for the given hypothesis, 3 to 20 as indicating positive evidence, 20 to 100 strong evidence, and greater than 100 very strong evidence. Bayes factors less than 1 indicate evidence in favor of the other hypothesis (Raftery, 1995).

### Process-level analysis

We examined the effect of race and other manipulations on the process using the DDM. To do so, we embedded the models within a hierarchical framework and used Bayesian estimation techniques to estimate the model parameters and the effects of the different conditions on those parameters (Kruschke, 2014; Lee & Wagenmakers, 2013). This hierarchical approach allowed us to reliably estimate the parameters of the DDM for the experimental designs used with the FPST, in which a large number of subjects complete a limited number of trials across several conditions. These designs are a challenge for conventional methods of fitting the DDM because the reliability and accuracy of the parameters are impacted (especially estimates of drift rates; Ratcliff & Childers, 2015). The hierarchical framework offers a solution to this problem by simultaneously modeling both individual- and group-level differences so that data from each participant inform the parameter estimates of the others.

**Fig. 2:**
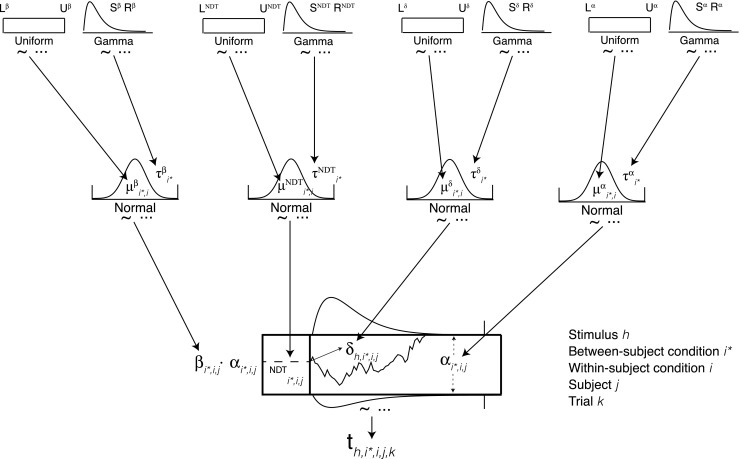
Diagram of the hierarchical drift diffusion model (DDM). The *k* th response time for subject *j* in within-subject condition *i*, between-subject condition *i* ^∗^, and with stimulus *h* is generated by a drift diffusion process. The markers on the normal distributions indicate that the priors were truncated. Similar markers placed on the DDM process indicate the possibility of modeling the censored data in Study 1 and 2, where choice and response time were not recorded if the response fell beyond the deadline

Figure 2 depicts the general hierarchical DDM. The Supplemental Material provides the JAGS code and the specifications of the priors used to estimate the model. The hierarchical structure means that each process parameter of the DDM had a higher order group-level prior. For example, the model encapsulated our beliefs in possible a priori values of the relative starting point for condition *i*, subject *j*, with a truncated normal distribution,

$$\beta_{i,j} \sim N(\mu^{\beta}_{i},\tau^{\beta}).$$

The normal distribution was truncated so that it fell between .1 and .9.^8^ The parameters *μ* *i* *β* and *τ* ^*β*^ are the mean and precision (the inverse of the variance) of the group-level distribution. Our prior beliefs in possible values of these hyperparameters were set to be uniform for the mean, and gamma distributed for the precision parameter.^9^

Figure 2 also has vertical lines at the tails of the response time distributions. This property reflects the fact that, in Studies 1 and 2, data outside the response window were censored (i.e., the observed response and response time were not recorded for trials in which the response was made outside the response window). This is a problem for the DDM and any model of the distribution of response times: If censoring is not accounted for, the distributions of response times will appear faster than the true empirical distribution, which will in turn impact the parameter estimates (e.g., increasing the magnitude of the estimated drift rates). The Bayesian approach makes it possible to build censoring directly into the model (Kruschke, 2014, p. 730) and we use this opportunity in Studies 1 and 2. More details are provided in the Supplemental Material.

As we have noted, many previous studies using the FPST have employed SDT to analyze choice data (Correll et al., 2002; 2007a, b; Greenwald et al., 2003; Kenworthy, Barden, Diamond, & del Carmen, 2011; Sadler, Correll, Park, & Judd, 2012; Sim et al., 2013). Therefore, for all the studies we report in this paper we also submitted the data to a Bayesian signal detection analysis (Lee, 2008; Lee & Wagenmakers, 2013). A full description of the SDT model, and the analysis are provided in the Supplemental Material. Our goal in doing this was to establish how the DDM gives a different, more complete, account of the data. In general, our analyses confirmed this showing that in addition to being unable to explain response times, the signal detection model was unable to identify a race bias in Study 1, incorrectly isolated a manipulation of discriminability in Study 3 to the criterion, and in general showed a varying effect of race on the decision criterion as the response window was manipulated across the four studies. Please see the Supplemental Material for more information.

#### Model estimation and specification

We estimated the posterior distributions over the parameters of the hierarchical models using Markov Chain Monte Carlo (MCMC) methods. These are numerical methods for approximating a distribution with a large representative sample. A full description of the estimation technique is provided in the Supplemental Material.

In parameterizing the DDM, we were guided by our two central hypotheses about how race impacts the decision process. This implied that the starting point, drift, and threshold should be allowed to vary as a function of the race of the target. To accomplish this, we let the group means of the DDM process parameters vary as a function of the race of the target as well as any of other experimental manipulation (e.g., context, discriminability). That is, we did not arbitrarily fix the DDM parameters to be equal across conditions and instead sought to examine how the data impacted (if at all) these parameters.

One question we did face was how to handle object type. The group means of the drift rates were allowed to vary as a function of object as well. This means the strength of the evidence for gun objects does not have to correspond to the strength of the evidence for non-gun objects, similar to other approaches that add a criterion to classify the evidence fed into the evidence accumulation process (see also Ratcliff, 1978; White & Poldrack, 2014).

However, one could ask if the other parameters also vary as a function of the object type. Mathematically, estimating the relative start point requires stimuli that on average point towards the upper boundary and stimuli that on average point to the bottom boundary (Link, 1978). Thus, the relative starting point must be fixed across the different object types.

To investigate the necessity of allowing the threshold separation and non-decision time to vary as a function of object type, we carried out a model comparison analysis where one or the other, both, and neither were allowed to vary at the group level as a function of object types. Using the Deviance Information Criterion (DIC; Spiegelhalter, Best, Carlin, & Van Der Linde, 2002) as measure of goodness of fit, all four studies showed that a model allowing both the threshold separation and non-decision time to vary as a function of object type provided a better fit to the data. However, based on two observations, we constrained the threshold separation to be constant across object type in all of our analyses. First, across all four studies, examination of the posterior estimates of the group-level mean threshold separation (*μ* ^*α*^) showed no or negligible effects of object type. Second, in another study where we manipulated the response window within subjects, we found that the threshold separation did not vary as a function of race. This finding was confirmed both with model comparisons using the DIC and by examining the posterior distributions (Johnson, Cesario, & Pleskac, 2017). For the rest of the article, “hierarchical DDM” refers to the model in which the relative starting point, threshold separation, drift rate, and non-decision time were allowed to vary as a function of race and all other experimental manipulations (e.g., context, discriminability), and only drift and non-decision time were allowed to vary as a function of object type as well.

In order to verify the appropriateness of the model for the FPST, we conducted a parameter recovery analysis of the hierarchical DDM. The analysis (reported in the Supplemental Material) showed that the model accurately and reliably recovered the parameters of the hierarchical DDM. We also conducted the posterior predictive checks for each study comparing the predicted and observed choice probabilities, mean response times, and response time distributions (see the Supplemental Material). The posterior predictive checks showed that the model gave a good account of the data across all four studies and all conditions. Nevertheless, future investigations should design studies better suited to evaluate the viability of more complex models, such as models including trial-by-trial variability in the parameters (Ratcliff, 1978; Ratcliff & Rouder, 1998) and multiple stages of processing (Diederich & Busemeyer, 2015).

#### Inferences from the hierarchical models

As our interest is on assessing how much and in which direction factors like race and context impact the decision process and the uncertainty in these effects, we take an estimation approach to our analyses (Gelman, Carlin, Stern, & Rubin, 2003, Kruschke, 2014). Thus, in our analyses, we report the mean posterior value and the 95% Highest Density Interval (HDI) in brackets next to the mean to describe the posterior distribution over the parameters. Values within the HDI are more credible (i.e., have higher probability density) than values outside the HDI, and the values within the HDI have a total posterior probability of 95%. To assess the effect of different conditions on the parameters, we report the difference between conditions in terms of the parameter value and the corresponding HDI as well as the differences in the estimates of the parameters standardized by their group-level variability in the parameter (e.g., $d = \frac {\mu ^{\delta }_{Black} - \mu ^{\delta }_{White}}{ \sqrt {1/\tau ^{\delta }} }$). Our focus, especially at this stage of study, is on estimating the effect of particular conditions, but in comparing the conditions we generally asked if the credible values contained 0 or not.

Taking this estimation approach does raise the question of whether we are begging the question, that is, presupposing a difference and testing the difference. To investigate just how well our hierarchical DDM can identify differences in the parameters, we simulated three different types of settings: (1) a difference between conditions in the relative start-point (*β*) but no other parameters, (2) a difference between condition in the drift-rates but no other parameters; and (3) a difference in the drift rates and a difference in the threshold but no other differences in the parameters. We then estimated the hierarchical DDM from each of these simulated datasets. Across all three settings, the hierarchical DDM does a good job of correctly identifying the true effect (> 92*%* of the time) and never incorrectly identified an effect in a different process parameter (see Supplemental Material for more details). We take this as evidence that our approach has good accuracy in terms identifying the effect of different factors on the decision to shoot.

Another Bayesian approach that could be taken is a model comparison approach that tests different hypotheses by comparing different models (e.g., Rouder, Speckman, Sun, Morey, & Iverson, 2009, 2012, 2016; Wagenmakers et al., 2010). This approach has several advantages including identifying a model that minimizes the chance of overfitting the data. However, we did not take this approach for several reasons. First, at this stage in the research our interest is on estimating the effect of the manipulation and our uncertainty in that effect on all the parameters. This, we feel, is the most informative approach in terms of uncerstanding how the process model accounts for this type of data. Second, our model recovery analyses give us confidence that we can reliably detect differences between conditions with the parameter estimates. Third, the conclusions from a model comparison approach are highly sensitive to the priors that are chosen whereas the parameter estimates are relatively robust. Thus, we rely on the Bayesian estimation approach (for further discussion on these issues see Gelman & Rubin, 1995; Kruschke & Liddell, in press; Kruschke & Vanpaemel, 2015; Lee, in press; Wagenmakers, Lee, Rouder, & Morey, in press, 2017).

Note that the posterior distribution, as examined in our Bayesian analysis, is the same regardless of the number of statistical tests conducted or the intentions of the experimenter (Kruschke, 2014). It depends only on the data and the specified model, including the priors and the likelihood function. Thus, there is no need to correct error rates for multiple comparisons or for the use of an omnibus test. Our analysis focused on examining the posterior distribution from the most informative angles in terms of how race and other factors impacted the decision process. We report these results in the paper. The Supplemental Material provides tables listing the main effects and interactions on each process parameter for the Bayesian hierarchical SDT model and the Bayesian hierarchical DDM.

## Study 1: what happens under conditions where race bias is predicted only in response times?

Study 1 might be regarded as a “standard” FPST design, with race manipulated within subjects, targets in neutral contexts, and the response window set at 850 ms. Past research has found that, with this response window, race bias emerges primarily in response times and not in error rates. That is, participants are faster to correctly shoot an armed Black target than an armed White target, but slower to correctly not shoot an unarmed Black target than an unarmed White target (Correll et al., 2002). A similar pattern of results emerges when trained police officers complete the task with shorter response windows (Correll et al., 2007; Sim et al., 2013). We expected to find the same pattern of results at the behavioral level, with race having an effect only on response times but not on errors. This expectation is a challenge for SDT (and for any theory that treats decision making as a static process), which fails to include time as an identifiable variable and thus is silent on the race bias in these datasets.

### Behavioral analysis

#### Response times

**Fig. 3:**
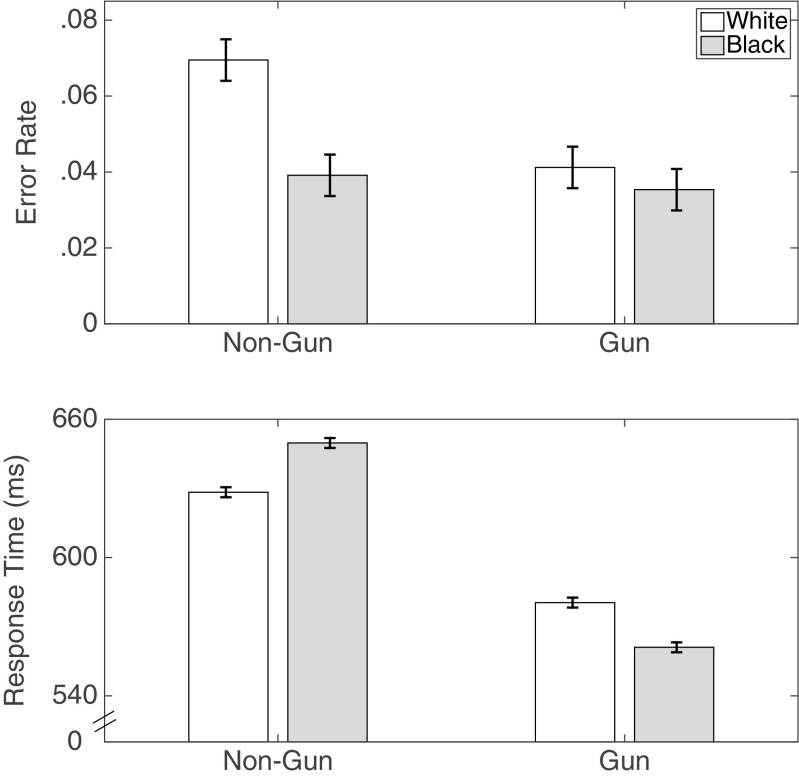
Error rates and response times for correct choices from Study 1. Error bars are 95% confidence intervals with the standard error estimated from the mean squared error of the interaction term between race and object from the ANOVA

Figure 3 displays the error rates and response times from Study 1. As expected, with an 850 ms window, there was a significant race by object interaction in response times, *F*(1,55) = 75.45, *p* < .001, ${\eta ^{2}_{p}} = .58$, *B* *F*_10_ > 1000.^10^ Participants were slower to correctly not shoot unarmed Black targets than unarmed White targets, *t*(55) = −6.50,*p* < .001, *B* *F*_10_ > 1000, but faster to correctly shoot armed Black targets than armed White targets, *t*(55) = 5.97,*p* < .001, *B* *F*_10_ > 1000. There was also a main effect for objects, such that participants were slower to correctly not shoot than shoot, *F*(1,55) = 349, *p* < .001, ${\eta ^{2}_{p}} = .86$, *B* *F*_10_ > 1000.

#### Error rates

Figure 3 also shows that there was an interaction in error rates between object and race, *F*(1,55) = 5.04,*p* = .03, ${\eta ^{2}_{p}} = .08$, *B* *F*_10_ = 3.01. However, the pattern of the interaction was not consistent with that typically reported in past studies: There were fewer errors for unarmed Black targets than for unarmed White targets (*t*(55) = −3.25,*p* = .002, *B* *F*_10_ = 14.99) and statistically no race differences in the error rates for armed targets.

Note also that the higher error rate for White armed targets led to a main effect of race, with more errors for (armed or unarmed) White target individuals, *F*(1,55) = 7.26, *p* = .01, ${\eta ^{2}_{p}} = .12$, *B* *F*_10_ = 7.35. Finally, consistent with past studies and with the point structure of the FPST, there was also a main effect of the object, with higher rates of shooting unarmed individuals (false alarms) than of not shooting armed individuals (misses), *F*(1,55) = 6.26, *p* = .015, ${\eta ^{2}_{p}} = .10$, *B* *F*_10_ = 4.13.

### Drift diffusion analysis

**Fig. 4:**
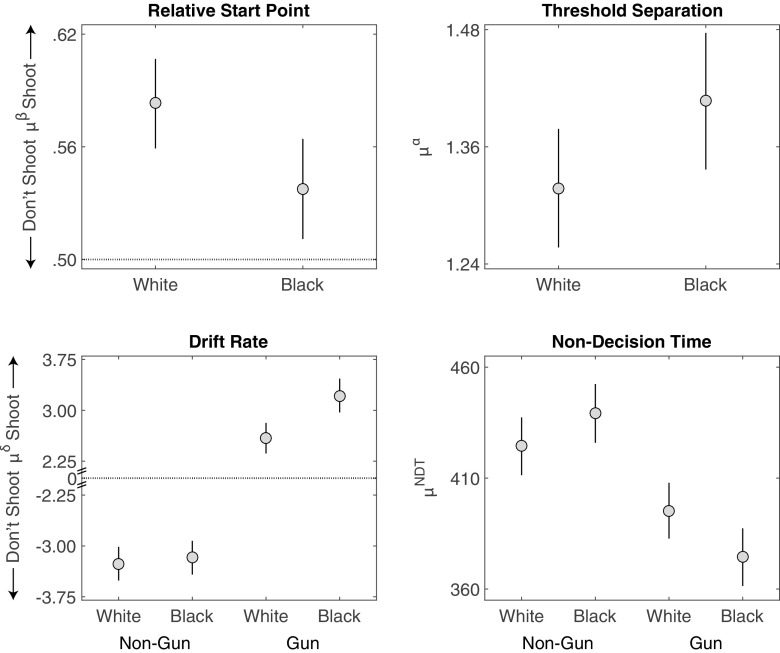
Study 1 posterior means (dots) and 95% HDI (bars) for the group-level parameter estimates of the DDM in each condition

Figure 4 displays the group-level estimates of the relative start point *μ* ^*β*^, threshold separation *μ* ^*α*^, drift rate *μ* ^*δ*^, and non-decision time *μ* ^*NDT*^.

#### Relative start point

We first turn to start point *β*, and ask: Were participants more inclined to shoot or not shoot at the start of the decision process, and did this inclination differ by target race? As Fig. 4 shows, participants were on average biased towards shooting, with an average relative start point above .5. This relative bias towards shooting was predicted in that the payoff structure encouraged shooting. This position of the relative start point explains why participants were on average slower to choose to not shoot as well as the higher rate of shoot decisions. It also speaks to the validity of the model, in that the estimated relative start point accurately reflected the payoff structure of the task.

With respect to the start point hypothesis, we did not find that the start point was biased towards shooting for Black targets. In contrast, the start points for Black targets were closer to the “Don’t Shoot” boundary than the start points for White targets were (*M* = −0.05 [−0.08,−0.01], *d* = −0.85 [−1.56,−0.14] ). This difference explains the lower level of errors for Black unarmed targets observed in this sample.

#### Threshold separation

Figure 4 also shows that participants tended to set a greater distance between thresholds (*μ* ^*α*^) for Black than for White targets, though the difference was not credible (*M* = 0.09 [−0.001,0.18], *d* = 0.60 [−0.002,1.22]).

#### Drift rate

Turning to the drift rates, we asked whether race influenced the strength of evidence of the gun and non-gun objects during evidence accumulation. The bottom left panel of Fig. 4 shows that, in this study, the effect of race on drift rates depended on the object. Race did not have a credible impact on the drift rates for non-gun objects (*M* = 0.09 [−0.26,0.43], *d* = 0.16 [−0.44,0.75]). There was, however, a credible difference in the drift rates for guns: Drift rates were larger for Black targets than for White targets (*M* = 0.62 [0.29,0.96], *d* = 1.07 [0.48,1.68]). That is, evidence to shoot had a faster rate of accumulation when a Black target was holding a gun than when a White target was holding a gun.

#### Non-decision time

Finally, non-decision time estimates were smaller for guns than for non-guns (*M* = −47.1 [−59.9,−34.1], *d* = −1.04 [−1.34,−0.74]), potentially due to the variety of non-gun objects used in the FPST. There was very little effect of race on non-decision times (*M* = −3.1 [−16.1,9.9],*d* = −0.07 [−0.36,0.22]). There was an interaction between race and object on non-decision times (*M* = 17.7 [4.8,30.5],*d* = 0.39 [0.11,0.68]). However, as this interaction was not observed in our other studies, we do no interpret it further.

### Interim conclusion

The results of Study 1 support the evidence hypothesis on the effect of race on the decision process. In particular, the drift rates for gun objects were higher for Black targets than for White targets, suggesting that the race of the target individual is processed as evidence when deciding whether or not to shoot.

This is a different understanding of the effect of race than the one provided by SDT, where the effect is typically isolated to the response process of setting a lower, more liberal criterion to shoot for Black targets. In fact, fitting SDT to this dataset shows no credible effect of race on the decision criterion (*M* = 0.13 [−0.01,0.26], *d* = 1.64 [−0.38,4.75]) (see Supplemental Material). If anything, as the estimates suggest, there was a trend for the opposite effect. Conventionally in the literature on the FPST this would be accepted because the race bias in Study 1 was only expected in the response times and not in error rates. We see this as a distinct advantage of the DDM in that it can can identify influences of race on decision parameters even in the presence of no race effects on error rates. Furthermore, as we will show across studies, regardless of how the race bias manifests itself in behavior, the DDM isolates the bias to a common source: evidence accumulation.

The DDM also identifies other potential effects of race beyond the biasing of racial stereotypes. In this study, participants appeared to have a starting point that was biased towards not shooting Black targets and, at the same time, a trend towards increasing the threshold separation for Black targets. Both of these results point towards participants working to counteract or control their prejudices. As these effects were small, however, we examined their robustness in the following studies.

## Study 2: how does context impact the decision process and the effect of race?

The goal of Study 2 was to examine how a shorter response window impacts the decision process. Behaviorally, past results have shown that, with a shorter response window, the race bias appears in error rates. Based on Study 1, the DDM should still isolate the effect of race to a change in the rate of evidence accumulation, while the change in response window should primarily impact the threshold participants set. This study also allowed us to investigate the context question: For half the subjects, the target appeared in a “dangerous” neighborhood and for the other half, in the same neutral context used in Study 1.

### Behavioral analysis

#### Error rates

**Fig. 5:**
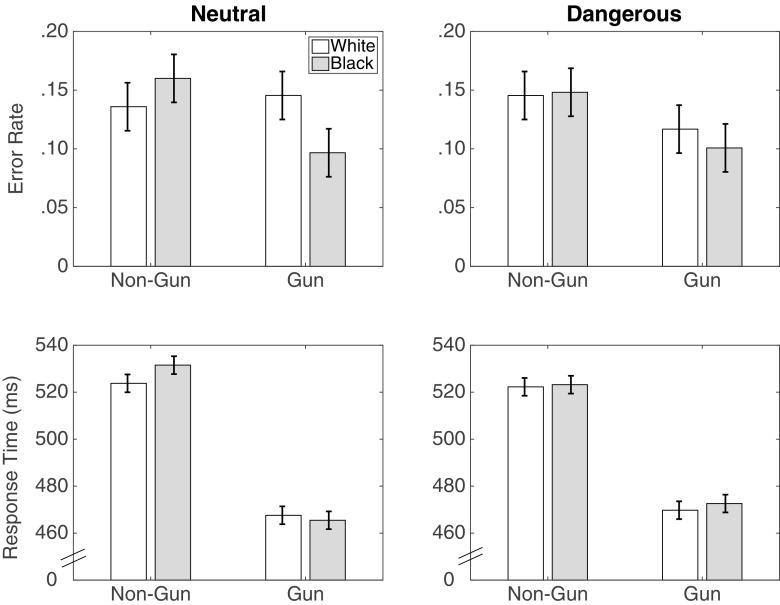
Error rates and response times for correct choices from Study 2. Error bars are 95% confidence intervals with the standard error estimated from the mean squared error of the interaction term between race, object, and context from the ANOVA

Figure 5 displays the error rates and response times in Study 2. The expected three-way interaction between object, race, and context on error rates did not reach conventional significance levels, *F*(1,114) = 3.69, *p* = .06, ${\eta _{p}^{2}} = .029$, *B* *F*_10_ = 0.022. Nevertheless, consistent with past studies, there was an interaction between race and object in the neutral condition *F*(1,57) = 14.07, *p* < .001, ${\eta _{p}^{2}} = .20$, *B* *F*_10_ = 5.46, but it dissipated in dangerous condition *F*(1,57) = 0.84, *p* = .36, ${\eta _{p}^{2}} = .02$, *B* *F*_10_ = 0.136. In the neutral condition, participants were more likely to incorrectly not shoot an armed White target than an armed Black target (misses), *t*(57) = −3.41, *p* < .001, *B* *F*_10_ = 23.09, but more likely (though not significantly so) to shoot an unarmed Black target than an unarmed White target (false alarms), *t*(57) = 1.66, *p* = .10, *B* *F*_01_ = 1.89.

#### Response times

With conventional frequentist tests there was a three-way interaction between object, race, and context (though the effect was small and the Bayes factors imply no effect), *F*(1,114) = 5.38, *p* = .02, ${\eta _{p}^{2}} = .05$, *B* *F*_10_ = 0.024. Participants were slower to correctly not shoot an unarmed Black target than an unarmed White target in the neutral condition (*t*(57) = 2.42, *p* = 0.02, *B* *F*_10_ = 2.08), but not in the dangerous condition.

### Drift diffusion analysis

**Fig. 6:**
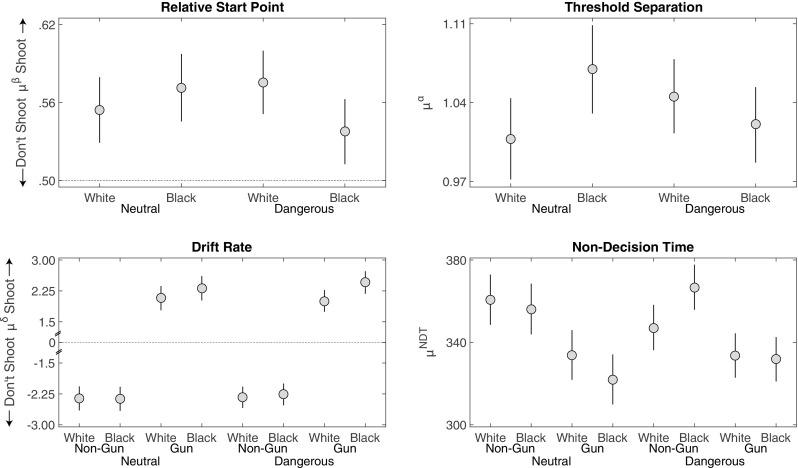
Study 2 posterior means (dots) and 95% HDI (bars) for the group-level parameter estimates of the DDM in each condition

Figure 6 displays the group-level estimates of the relative start point *μ* ^*β*^, threshold separation *μ* ^*α*^, drift rate *μ* ^*δ*^, and non-decision time *μ* ^*NDT*^. A complete analysis of the effect of the manipulations on the process parameters is provided in the Supplemental Material.

#### Relative start point

There was no credible race difference in the relative start point (*M* = −0.01 [−0.04,−0.01], *d* = −0.16 [−0.55,0.22]), nor was there any credible effects of context or an interaction.

#### Threshold separation

There are two important observations from the threshold separation estimates in Study 2. First, relative to Study 1, participants had a lower threshold (Table 2). This difference in thresholds is consistent with an *a priori* property of the DDM, namely, that as time pressure increases the threshold separation should decrease, thus trading accuracy for speed. We return to this result in the composite analysis, where we model all common conditions of the four studies simultaneously. Nevertheless, this result, as well as the starting point bias towards the “Shoot” option, speaks to the validity of the model to meaningfully measure properties of the decision process.

Consistent with the trend we saw in Study 1, we found that participants set higher thresholds for Black targets in the neutral contexts (*M* = 0.06 [0.01,0.12],*d* = 0.82 [0.11,1.57]). However, in the dangerous contexts, there was no credible difference between Black and White targets (*M* = −0.02 [−0.07,0.02],*d* = −0.32 [−0.95,0.29]). As Fig. 6 shows, threshold separations in the dangerous condition fell largely between those of Black and White targets, respectively, in the neutral condition.

#### Drift rate

Turning to drift rate differences the rate of evidence accumulation was higher for armed Black targets than for armed White targets though the effect was smaller than in Study 1 (*M* = 0.34 [0.06,0.62],*d* = 0.43 [0.08,0.79]). Nevertheless consistent with Study 1 a gun provided stronger evidence toward the “Shoot” decision when held by a Black target than when held by a White target. Also like Study 1 there was very little effect of race on the non-gun object (*M* = 0.03 [−0.24,0.31],*d* = −0.04 [−0.31,0.38]). Context did not have a credible effect on the drift rates for gun or non-gun objects, nor was there an interaction between race and object for the gun or non-gun object.

**Table 2:** Summary statistics of the posterior estimates of the group level mean threshold separation *μ* ^*α*^ collapsed across conditions for each study

	Mean	95% HDI
Study 1 (850 ms)	1.36	[1.27, 1.46]
Study 2 (630 ms)	1.04	[0.98, 1.09]
Study 3 (750 ms)	1.10	[1.03, 1.17]
Study 4 (630 ms)	0.99	[0.95, 1.03]

#### Non-decision time

The group-level mean non-decision time estimates also showed the same shift to smaller magnitudes for gun objects (*M* = −27.3 [−35.3,−19.1],*d* = −0.65 [−0.84,−0.45]). Again, there were also some apparent interactions between race and context in the non-decision time estimates; however, these interactions did not replicate in subsequent studies so we refrain from further interpretation.

### Interim conclusion

The results of Study 2 show that, as in Study 1, participants were quicker to accumulate evidence towards shooting when a Black target was armed than when a White target was armed, and that this held in both neutral and dangerous contexts. This result implies that participants use both the object and the target—at least for armed targets—to decide between “Shoot” and “Don’t Shoot,” and that this bias is present regardless of the context.

Study 2 found no credible effect of race on the relative start point. However, we did find that in the neutral condition (of this between-subjects manipulation) participants set a credibly larger threshold separation, and thus exhibited more caution for Black targets. This result is consistent with the trend we observed in Study 1. In Study 2, this difference dissipated in dangerous contexts. In fact, it appears that participants responded to the dangerous condition by seeking to collect a little more information before deciding to shoot, regardless of target race.

## Study 3: how does discriminability of the object impact the decision process in the FPST?

In Study 3, we sought to replicate the basic effects of race and context on the decision process. To further test the effect of the response window on the threshold separation *α*, we used a response window of 750 ms and predicted that the threshold separation would fall between that of Study 1 (850 ms) and Study 2 (630 ms). Finally, to address our discriminability question, we blurred the object shown to participants in half of the trials by using photo manipulation software to “smudge” it. As discussed earlier, changing the discriminability of objects can provide information on the evidence being extracted from the objects. In particular, it can help reveal if the non-gun objects carry no information pertinent to the shoot decision, as assumed by the typical SDT analysis, or if the the non-gun objects convey information as to the the shoot decision. If there is no information then blurring the non-gun objects should have no effect on the decision in these trials, but if there is some information then blurring them should decrease false alarms.

### Behavioral analysis

#### Error rates

**Fig. 7:**
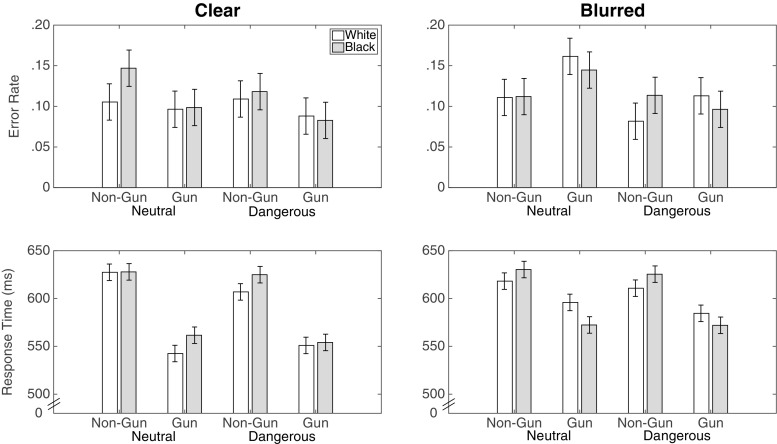
Error rates and response times for correct choices from Study 3. Error bars are 95% confidence intervals with the standard error estimated from the mean squared error of the interaction term between race, object, context, and discrimination, from the ANOVA

Figure 7 displays the error rates and response times from Study 3. Consistent with a race effect conventional p-values indicated a two-way interaction between race and object in the error rate, *F*(1,37) = 8.14,*p* = .007, ${\eta _{p}^{2}} = .180$, *B* *F*_10_ = 0.518. There was a greater proportion of incorrect choices to shoot unarmed Black than unarmed White targets (.12 vs .10), *t*(37) = 2.698, *p* = .010, *B* *F*_10_ = 4.01. However, there was not a significant difference in the proportion of incorrect choices to not shoot armed Black vs. armed White targets (.11 vs. .12). There was also an interaction between race and object in response times, *F*(1,37) = 5.55,*p* = .024, ${\eta _{p}^{2}} = .131$, *B* *F*_10_ = 0.032. Participants were significantly slower to correctly not shoot unarmed Black targets (627 ms) than unarmed White targets (616 ms), *t*(37) = 2.48,*p* = .013, *B* *F*_10_ = 2.56, but there was not a significant difference in response times for correctly shooting armed Black (565 ms) vs. armed White targets (568 ms). Thus, in Study 3, we again found support for the typical race effect on error rates and response times, though the Bayes factors for these results suggest caution in interpreting them. Moreover, in a departure from the findings of Correll et al. (2011) and to some degree Study 2, none of these effects depended on context.

**Fig. 8:**
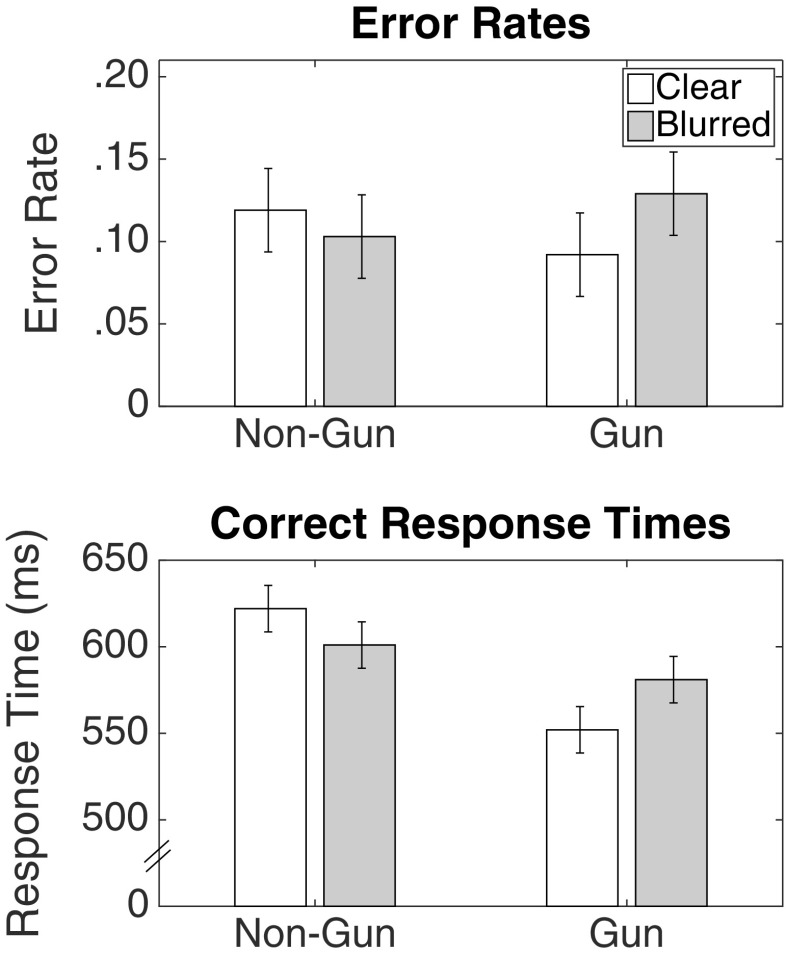
The effect of the manipulation of discrimination on error rates and response times for correct choices from Study 3. Error bars are 95% confidence intervals with the standard error estimated from the mean squared error of the interaction term between race, object, context, and discrimination, from the ANOVA

The new manipulation in Study 3 was the discrimination manipulation. Discrimination did not interact with the race manipulation. However, Fig. 8 shows that it did affect the processing of the object. In particular, there was an interaction between the discriminability of the object and the type of object, *F*(1,37) = 18.84, *p* < .001, ${\eta _{p}^{2}} = .337$, *B* *F*_10_ = 87.99. When a non-gun object was blurred, there was a significant decrease in the proportion of incorrect choices to shoot unarmed targets (.12 for clear vs .10 for blurred conditions), *t*(37) = −2.50,*p* = .016,*B* *F*_10_ = 2.67. Yet, when the gun was blurred, there was a significant increase in the proportion of incorrect choices to not shoot armed targets (.09 for clear vs. .13 for blurred), *t*(37) = 4.12,*p* < .001,*B* *F*_10_ = 125.1. This simultaneous increase in incorrectly not shooting armed targets (misses) and decrease in incorrectly shooting unarmed targets (false alarms) suggests that both the gun and non-gun objects conveyed information that swayed participants towards shooting.

This outcome is particularly problematic for signal detection analyses, which assume that the non-gun objects provide no signal for the shoot decision (i.e., they are just noise). As a result, in terms of the manipulation of discriminability, the SDT model isoloates the effect of the discrimination manipulation of the criterion estimates, which were larger when the objects were blurred than when they were clear (*M* = 0.15 [0.08,0.21],*d* = 2.05 [0.46,4.53]). There was no credible difference between blurred and non-blurred objects in terms of sensitivity to shoot (*M* = −.14 [−0.37,0.10],*d* = −0.16 [−0.43,0.12]) (see Supplemental Material). The effect of discriminability on the decision criterion highlights the difficulty that the SDT model has in properly characterizing this property. This is due to the fact that apparently non-gun objects provided some signal for the shoot decision. As a result, blurring gun and non-gun objects lessened the strength of the information for shooting for both objects. Because the SDT model assumes that the non-gun (i.e., noise) distribution is fixed on zero, it reflects this change as a shift in criterion.^11^

#### Response times

Consistent with the error rates, the discrimination manipulation also had an effect on the observed response times. In particular, the effect of blur depended on the object type, *F*(1,37) = 10.72, *p* = 0.002, ${\eta _{p}^{2}} = 0.225$, *B* *F*_10_ = .125. Participants were slower to correctly shoot an armed target when the object was blurred (552 ms for clear vs. 581 ms for blurred), *t*(37) = 6.14,*p* < .001,*B* *F*_10_ = 048. However, there was no significant difference in response times when the target was unarmed (622 ms for clear vs. 601 ms for blurred).

### Drift diffusion analysis

**Fig. 9:**
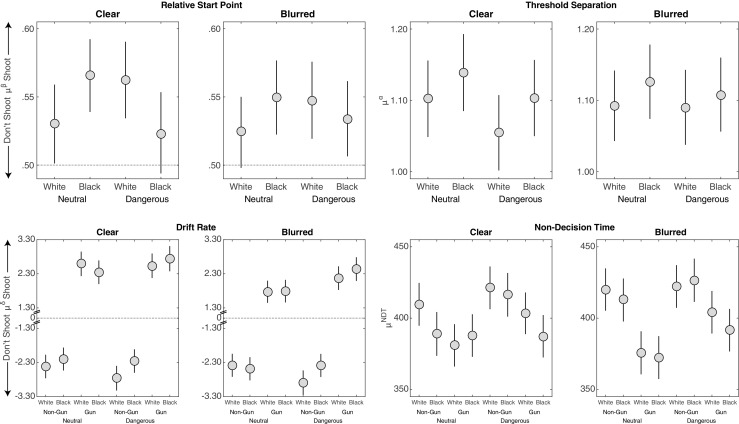
Study 3 posterior means (dots) and 95% HDI (bars) for the group-level parameter estimates of the DDM in each condition

Figure 9 summarizes the posterior distributions of the group estimates for the starting bias *μ* ^*β*^, threshold separation *μ* ^*α*^, drift rate *μ* ^*δ*^, and non-decision time *μ* ^*NDT*^.

#### Relative start point

Consistent with the other analyses, while there was an initial bias towards shooting, race did not have a credible effect on the relative response bias.

#### Threshold separation

As predicted, threshold separation for Study 3 fell between that of Study 1 and Study 2 (see Table 2). Similar to Studies 1 and 2, there was a trend to greater threshold separation for Black than White targets (*M* = 0.03 [−0.00,0.07],*d* = 0.32 [−0.03,0.68]). In contrast to Study 2, the effect of race on threshold separation did not depend on context (*M* = −0.001 [−0.04,0.04],*d* = −0.01 [−0.35,0.35]).

#### Drift rate

In contrast to the other two studies, we did not find a credible difference between the drift rates for White and Black armed targets (i.e., the gun drift rate) (*M* = 0.06 [−0.18,0.31],*d* = 0.07 [−0.22,0.38]). Instead, in Study 3, the race effect was on the non-gun objects, with the drift rate for unarmed Black targets being weaker for not shooting than that for unarmed White targets (*M* = 0.28 [0.04,0.52],*d* = 0.34 [0.05,0.64]).

Figure 9 also shows that the effect of context in Study 3 was partially isolated to the drift rates associated with the gun objects. In particular, the drift rates for armed targets were larger in dangerous contexts (*M* = 0.34 [0.10,0.59],*d* = 0.42 [0.12,0.72]), suggesting that dangerous contexts in this study elicited greater sensitivity to stimulus information when manipulated within subjects.

As expected, drift rates were also impacted by the manipulation of discriminability. Blurring the object led to a decrease in drift rates for armed targets holding a blurred gun relative to a non-blurred gun (*M* = −0.51 [−0.76,−0.27],*d* = −0.62 [−0.93,−0.32]). There was not a credible difference for unarmed targets, although blurring non-gun objects did on average lead to a decrease in drift rates for non-gun objects (i.e., drift rates pointed more strongly towards “Don’t Shoot”) (*M* = −0.13 [−0.38,0.11],*d* = −0.16 [−0.46,0.13]).

#### Non-decision time

Finally, there were two interpretable effects on non-decision time. As in the earlier studies, non-decision times were larger for non-gun than for gun objects (*M* = 26.9 [19.4,34.3],*d* = −0.61 [−0.79,−0.44]). Non-decision times in Study 3 were also larger in the dangerous condition than in the neutral condition (*M* = 15.4 [7.7,22.9],*d* = 0.35 [0.18,0.53]). Paired with the change in drift rates, one post hoc explanation for this effect is that the within-subjects design may have led to different encoding strategies between neutral and dangerous contexts, resulting in different non-decision times and drift rates. However, we did not find a consistent impact of context on the decision process across our three studies, suggesting that caution is warranted in interpreting this result.

### Interim conclusion

Decision processes in Study 3 were similar to those observed in the other studies, but some differences did emerge. As in all previous analyses, we relative start points were not larger for Black targets (i.e., start point hypothesis). Threshold separations were, on average, larger for Black targets, but as in the other studies the effect was not large.

Race also impacted evidence accumulation. In contrast to Studies 1 and 2, however, the effect was on non-gun objects, with Black unarmed targets having drift rates that were weaker towards not shooting than White unarmed targets. This type of race bias is particularly alarming as it leads to more false alarms or shooting of unarmed Black targets than unarmed White targets. The effect of race on the drift rates for unarmed targets in Study 3 is symmetrical with the effects of race on the drift rates for armed targets in Studies 1 and 2. Either one is sufficient to produce the race bias (i.e., an interaction between race and object) observed in error rates or response times.

The discrimination manipulation cast light on the properties of the information gleaned from the scene. Blurring the objects reduced the hit rate (shooting armed targets) and the false alarm rate (shooting unarmed targets).^12^ Whereas the SDT model isolates this effect of the blur to a bias in the response, the DDM—through its ability to separately model the quality of the evidence for gun and non-gun objects—attributes it to a reduction in the strength of the information towards shooting. Moreover, the drift rates from the DDM suggest (as one might expect) that this information was weak in the non-gun objects.

The context manipulation in Study 3 led to an increased drift rate and increased non-decision times. As mentioned, one post-hoc interpretation is that the within-subjects design may have led to different encoding strategies between neutral and dangerous contexts. In contrast, Study 2, which used a between-subjects manipulation of context, isolated the context effect to the threshold separation. Because of these conflicting results as well as the differences in the race effect (which emerged for armed vs. unarmed targets), we ran a final experiment with a larger sample size with the goal of addressing these differences between studies.

## Study 4: using a larger sample size to isolate the effects of race and context

Across Studies 1, 2, and 3, we consistently found that the observed race bias was isolated to the drift rates of the DDM, supporting the evidence accumulation hypothesis. However, in Studies 1 and 2 the effect was on the gun objects, whereas in Study 3 it was on the non-gun objects. In addition, Studies 2 and 3 identified different effects of context on the decision process, with Study 2 isolating the effect of context to changes in threshold separations and Study 3 isolating the effect to non-decision time and drift rates. One possible reason for this difference is that context was manipulated between subjects in Study 2 but within subjects in Study 3.

To try to better isolate the effects of race and context, we conducted a fourth study with a much larger sample size (*N* = 108), with each participant completing twice as many trials per condition (*n* = 40). As in Study 2, we set the response window to 630 ms. We therefore expected the race effect to appear in the error rates at the behavioral level, and the threshold separation to be similar in magnitude to Study 2. We manipulated race and context within subjects.^13^

### Behavioral analysis

#### Error rates

**Fig. 10:**
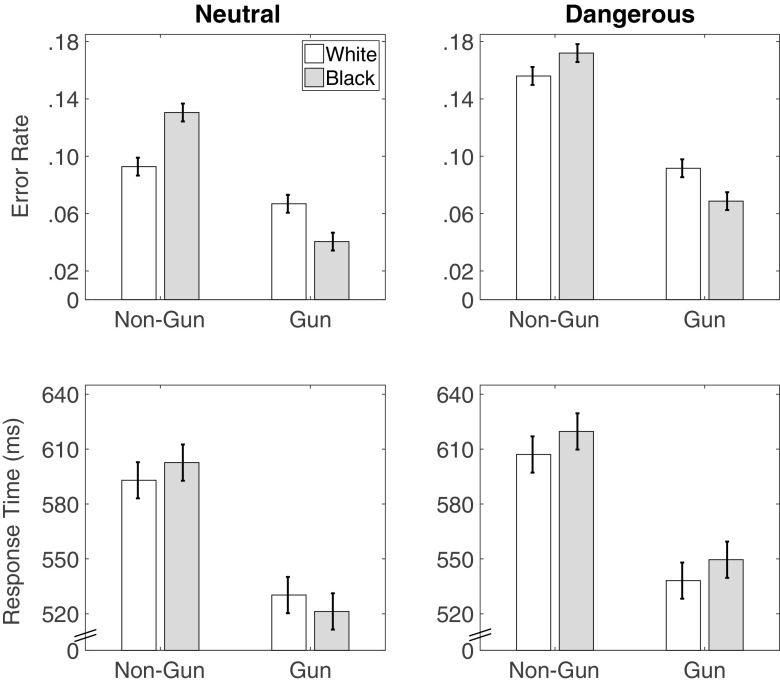
Error rates and response times for correct choices from Study 4. Error bars are 95% confidence intervals with the standard error estimated from the mean squared error of the interaction term between race, object, and context, from the ANOVA

Figure 10 shows the error rates and correct response times from Study 4. The standard race effect was present in the data, with a two-way interaction between race and object in the error rate, *F*(1,107) = 37.94,*p* < .001, ${\eta _{p}^{2}} = .26$, *B* *F*_10_ = 36.77. There was a greater proportion of incorrect choices to shoot unarmed Black than unarmed White targets (.31 vs .28), *t*(107) = 4.58, *p* =< .001, *B* *F*_10_ > 1000, and a lower proportion of incorrect choices to not shoot armed Black than armed White targets (.22 vs. 24), *t*(107) = −4.17, *p* =< .001, *B* *F*_10_ > 1000.

#### Response times

There was not a significant interaction between race and object in response times. Thus, in Study 4, consistent with the literature and our own results with a response window of 630 ms, we found evidence for the typical race effect on error rates. Replicating the results of Study 3 and departing from Study 2 and the findings of Correll et al. (2011), the race bias did not depend on context, nor was there an overall effect of context on response times.

### Drift diffusion analysis

**Fig. 11:**
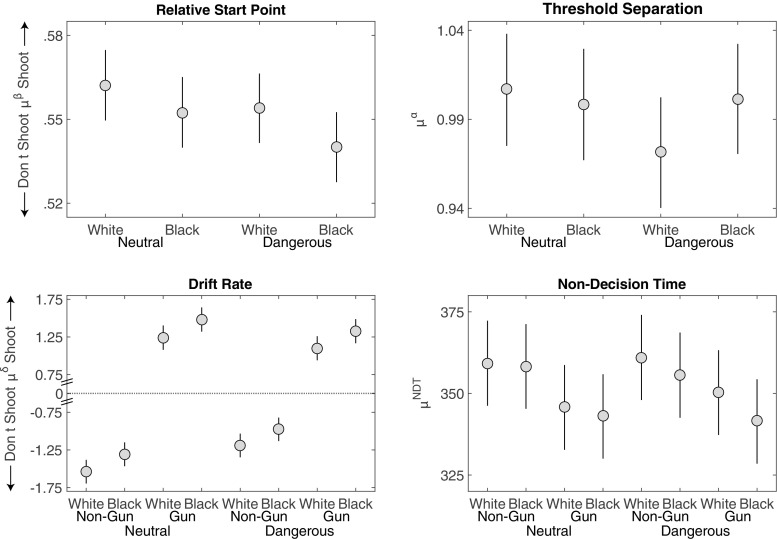
Study 4 posterior means (dots) and 95% HDI (bars) for the group-level parameter estimates of the DDM in each condition

Figure 11 summarizes the posterior distributions of the group estimates for the relative start point *μ* ^*β*^, threshold separation *μ* ^*α*^, drift rate *μ* ^*δ*^, and non-decision time *μ* ^*NDT*^.

#### Relative start point

As in the other studies, there was an initial bias towards shooting, and race did not have a credible effect on the relative start point (*M* = −0.01 [−0.02,0.004],*d* = −0.24 [−0.50,0.02]). If anything, as in Study 1, there was a trend for lower relative start points for Black targets.

#### Threshold separation

As predicted, the threshold separation parameter was in a similar range as in Study 2 (Table 2). However, we found no credible difference in threshold separation between Black and White targets (*M* = 0.01 [−0.02,0.04],*d* = 0.07 [−0.14,0.27]). There was also no credible effect of context on thresholds.

#### Drift rate

The bottom left panel of Fig. 11 shows that race impacted the drift rates for both armed and unarmed targets. As in Studies 1 and 2, the drift rate was greater in magnitude for armed Black targets than for armed White targets (*M* = 0.24 [0.08,0.39],*d* = 0.33 [0.10,0.55]). Moreover, replicating Study 3, we also found that the drift rate was greater in magnitude for unarmed Black targets than for unarmed White targets (*M* = 0.22 [0.06,0.38],*d* = 0.31 [0.09,0.53]). This simultaneous effect of race on both armed and unarmed targets is the strongest form of the race bias and explains the complete cross-over interaction observed in the error rates.

We should also note that, consistent with the larger error rates in the dangerous context, especially for non-gun objects, drift rates for non-gun objects were smaller in magnitude (closer to 0) in dangerous contexts (*M* = 0.34 [0.18,0.50],*d* = −0.48 [−0.26,0.70]). A similar trend was apparent for gun objects (*M* = −0.15 [−0.31,0.01],*d* = −0.20 [−0.43,0.02]).

#### Non-decision time

Finally, as the bottom right panel of Fig. 11 shows, non-decision times were larger for non-gun than for gun objects (*M* = 13.2 [4.0,22.3],*d* = −0.19 [−0.33,−0.06]).

### Interim conclusion

Study 4 yielded three main results. First, it provided further support for the evidence accumulation hypothesis, with the race of the target impacting the drift rates of both armed and unarmed targets. Thus, across all four studies, the DDM shows that the race of the target enters the decision as information that is accumulated over time.

Second, in contrast to the other studies, we did not find increased response caution in response to Black targets. This raises the question of how much empirical support there is for an increase in threshold separation for Black targets. We address this question next, using the Bayesian hierarchical DDM to model the effect of race across all four studies.

Third, changing the background scenes from neutral to dangerous scenes in Study 4 led to yet another effect, namely, a decrease in the magnitudes of the drift rates. That is, in each study in which context was manipulated, we observed a different result. We believe these unreliable effects of context speak against the interpretation of Correll et al. (2011) that the type of neighborhood serves as a reliable cue in deciding to shoot.

## Composite analysis of the race manipulation

As a final step in using the DDM to understand how race impacts the decision process, we fit the hierarchical DDM to the data from all four studies simultaneously.^14^ In doing so, we used only the conditions of the FPST that were common across all four studies, namely, those in which targets appeared in front of a neutral background holding a non-blurred object. To maintain consistency, we used the same model as in all the other studies, treating experiment as another condition, so that each group-level mean process parameter was allowed to vary between experiments as well as between the race conditions. Thus, this analysis allowed us to investigate how race influenced the process parameters across all four studies. Moreover, because the response window changed between the experiments, we can examine the effects of the response window not only on the threshold separation, but also on the other parameters of the DDM.

**Fig. 12:**
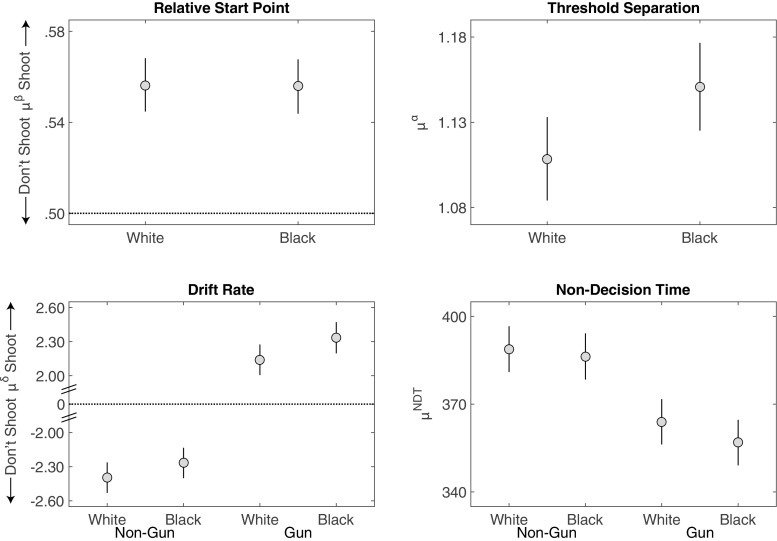
Posterior means (dots) and 95% HDI (bars) for the group-level parameter estimates of the DDM in the common conditions across all four studies

**Fig. 13:**
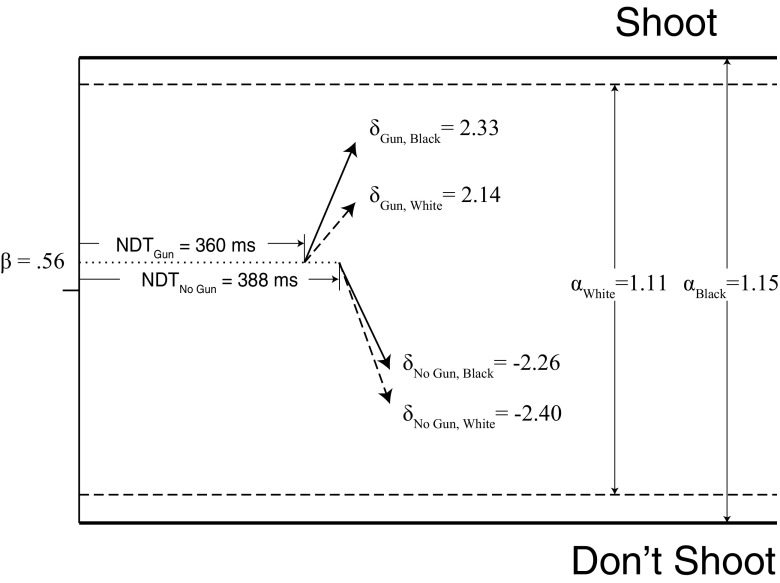
Illustration of the effect of race on the drift diffusion parameters. Note that we show the drift rates for non-gun objects for Black and White targets although the difference between these two parameters did not exclude 0 with a 95 %HDI

Figure 12 displays the group-level parameter estimates of the DDM averaged across all four studies as a function of the race of the target. A stylized summary of how the race of the target impacted the decision process is given in Fig. 13. This composite analysis shows that, consistent with the point scheme of the FPST, there was an initial bias towards shooting, but no effect of race on the relative start point (*M* = −0.003 [−0.02,0.02],*d* = −0.01 [−0.31,0.30]). In terms of thresholds, across all four studies there was a credible increase in the threshold separation for Black targets (*M* = 0.04 [0.01,0.08],*d* = 0.31 [0.05,0.58]).

Across the studies, the race of the target impacted the evidence that participants accumulated. In the composite analysis, this race effect is primarily driven by the gun objects, with the drift rates being greater in magnitude for armed Black targets than for armed White targets (*M* = 0.19 [0.01,0.39],*d* = 0.25 [0.01,0.51]). The drift rates for unarmed Black targets were also larger than those for unarmed White targets, but the effect was smaller (*M* = 0.13 [−0.05,0.32],*d* = 0.17 [−0.08,0.42]). Neither of these differences depended on the size of the response window (or study) (see Supplemental Material). In comparison, using SDT to examine this combined dataset would suggest that the effect of race on the response criterion did depend on the response window (*M* = 0.08 [0.01,0.14],*d* = 0.43 [0.07,0.79]) (see Supplemental Material). We believe that this interaction between race and response window clearly illustrates the weakness of SDT as a model of the decision to shoot during the FPST.

The race of the targets did not affect the non-decision times. However, non-decision times were larger for non-guns than for guns (*M* = 27.1 [29.2,34.7],*d* = 0.47 [−0.61,−0.34]).

The composite analysis also allowed us to examine how the response window impacted decision processes. As the response window increased across studies, threshold separation increased by on average 0.22([0.19,0.26]; *d* = 1.64 [1.35,1.94]) (Table 2). Some studies have shown that changes in time pressure, like the changes in the response window implemented in our studies, do not solely impact the threshold separation (i.e., time pressure may not have a selective influence on the threshold separation). Rather, decreases in time pressure have also been associated with stronger drift rates (Rae, Heathcote, Donkin, Averell, & Brown, 2014) as well as with an increase in non-decision time (Voss et al., 2004). We also found both of these effects. As response windows increased, drift rates for guns increased by on average 0.94([0.75,1.13]; *d* = 1.23 [0.98,1.50]), drift rates for non-gun objects decreased (i.e., grew stronger) by − 0.90([−1.09,−0.71]; *d* = −1.18 [−1.44,−0.93]), and non-decision times increased by on average 53.2 ([45.3,61.0]; *d* = −0.47 [−0.61,−0.34]).

## General discussion

In this article, we developed and tested a formal framework for modeling the decision to shoot in the FPST as a dynamic stochastic process. The modeling framework assumes that the decision unfolds as a drift diffusion process and accounts for both choice and response time distributions simultaneously. This stands in contrast to existing approaches, both with the FPST and more generally in the area of social cognition, which typically provides no way of understanding choices and response times within the same formal model. A second feature of the model is that it is embedded within a Bayesian hierarchical framework, which, as we have shown, makes it possible not only to model choices and response times, but also to characterize and measure the effect of different factors on the decision process at both the group and individual level within experimental designs widely used in social psychology. Importantly we see this work as providing a crucial foundation to start to better understand the decision to shoot. From this foundation we can establish methods to better characterize race bias and understand how the decision to shoot is made. In order to take these important steps one must establish a formal modeling framework of the processes underlying the decision to shoot. This is what we have sought to do here. Next, we review the implications of our findings with respect to the process parameters of the DDM and use those implications to map out the next steps in this approach. We also address the limitations of our sample, task, and approach, in modeling the decision to shoot.

### The effect of race on drift rates

The DDM provides an interesting and novel process account of the role of race in decisions to shoot during the FPST. This dynamic account is perhaps more complicated than that provided by SDT. However, it also appears to be more complete and integrative. Across all four studies, we found that the strength of the evidence participants accumulated in deciding between the “Shoot” and “Don’t Shoot” option depended on the race of the target (the Evidence Hypothesis). In Studies 1 and 2, when the target was armed (i.e., holding a gun), the rate of evidence accumulation towards the “Shoot” option was much faster for Black targets than for White targets. Thus, participants made fewer errors for armed Black targets and were faster to correctly choose to shoot Black targets. In Study 3, when the target was unarmed (i.e., holding a non-gun), the rate of evidence accumulation towards the “Don’t Shoot” option was weaker (or less negative) for Black targets, leading to more errors in incorrectly shooting unarmed Black targets and to participants being slower to correctly not shoot Black targets. In Study 4, race effects were observed for both gun and non-gun objects. As mentioned earlier, these differences in the race effect being isolated to gun, non-gun, or both objects, are consistent with the mixed results from previous studies, which have reported the race by object interaction at the behavioral level to be the result of a difference in unarmed targets (Plant & Peruche, 2005), armed targets (Study 2 in Correll et al., 2002), or both (Correll et al., 2011). An advantage of the DDM is that we can more precisely isolate the driver of these results to the accumulation of evidence. Across the studies, our results tend to suggest the race effect is more pronounced for gun objects, perhaps reflecting the nature of the stereotype expectancy that drives the behavioral bias (i.e., that Blacks are expected to have guns, not that Whites are expected to have non-guns).

This understanding of how race impacts the decision process differs from that offered by SDT, which has focused on the decision criterion. As we have shown across the four studies, the DDM account provides a much more consistent and parsimonious explanation for the data. There are two different explanations of this shift in drift rates for Black vs. White targets. One explanation is that the difference in drift rates means that—instead of collecting evidence solely in terms of the presence of a gun—participants process both the object and the race of the target in determining whether or not to shoot. Thus, not only does this result resonate with past accounts suggesting that stereotypes enter the decision process via information processing (Payne, 2005, 2006; Plant et al., 2005), it is also consistent with accounts suggesting that participants base their decision on the perceived threat of the target (Correll et al., 2002, 2011).

A second explanation is analogous to signal detection theory. In this case, the object gives rise some underlying information in terms of threat or the match to a prototypical gun. The information is compared to a criterion transforming it into evidence for shooting or not and then the evidence is accumulated (Ratcliff & McKoon, 2008). According to this mechanism, a lower drift criterion is used for Black targets than White targets so that a larger range of the information extracted from the scene is transformed into evidence supporting “Shoot.” Our data and models cannot distinguish between these two different explanations. Nevertheless, in both cases the result is the same in that the effect of race is isolated to the evidence accumulation process.

Finally, it is worth mentioning that the DDM we used does not explicitly assume an order in which aspects of the scene are processed. However, the lack of a race effect on response bias suggests that, at least in our data, the race of the target may not have been consistently processed first. Yet there certainly are situations in which participants first process the race of the person and then the object (or vice versa). Indeed these or similar studies have been conducted (see for example Payne, 2001). The DDM can be expanded to account for these different processing orders by making the drift rate a function of the aspect being attended to (e.g., object, race of the target). Such an expanded view has the potential to reveal a rich set of choice and response time patterns (Diederich & Busemeyer, 2015).

### The effect of race on threshold separation

The DDM also reveals a second pathway by which race impacts the decision to shoot in the FPST, namely, via the effect on threshold separation. In particular, in some conditions we found that participants set larger threshold separations for Black targets than for White targets and thus required more evidence before making a decision on Black targets. Insofar as the threshold indexes an underlying psychological process, this may be an attempt to strategically counteract a race bias, perhaps reflecting a motivation to control prejudice (Plant & Devine, 1998). All else being equal, an increase in threshold separation for Black targets would result in more accurate performance in these trials. Indeed, in Study 1 as well as other previous studies (Ma Correll, Wittenbrink, Bar-Anan, Sriram, & Nosek, 2013; Sadler et al., 2012; Sim et al., 2013) (see also Plant et al., 2005, for a similar result in the process-dissociation model), sensitivity in terms of *d* ^′^ was larger for Black targets than for White targets (see Supplemental Material).

In terms of reducing the observed race bias in errors, this change in threshold can be partially effective in that it can reduce the difference in the rates of Black and White unarmed targets being incorrectly shot. However, this strategy does not come without costs: it also leads to a larger difference in errors for armed targets, with even fewer “Don’t Shoot” decisions for armed Black (vs. White) targets and increased response times for Black targets. Moreover, as should be clear, this strategy does not change the race bias that is present in the actual accumulation of evidence (i.e., the drift rates).

We believe the opposing forces of the race effect observed in threshold separation and drift rate illustrate the advantage of DDM to reveal the complex effect of race on the decision to shoot. The change in threshold separation may provide a new perspective on the control processes that participants use to counteract race biases. Control processes have typically been discussed in the context of dual-process models, where two qualitatively different systems produce different responses to the task at hand (Bargh, 1999; Chaiken & Trope, 1999; Evans & Frankish, 2009; Sherman, Gawronski, & Trope, 2014; Sloman, 1996): The fast, more automatic, unintentional system produces the response based on the stereotypic association, whereas the slower, more controlled, intentional system produces the response based on the relevant information. The DDM and the threshold separation parameter show how processes typically considered to be under conscious control may influence response times at speeds of responding typically thought to capture automatic processes. This approach offers an important answer to why and how the amount of time participants have to make a decision impacts the observed decision by showing why changes in the response window impact error rates. Finally, the role of controlling the threshold separation also opens up new questions. For instance, recent work has begun to identify the neural circuitry involved in setting levels of response caution (i.e., threshold separation) during low-level perceptual decision tasks (Forstmann, Anwander, Schäfer, Neumann, Brown, Wagenmakers, & Turner, 2010; van Maanen, Brown, Eichele,Wagenmakers, Ho, Serences, & Forstmann, 2011), raising the intriguing question of whether and how these processes play a role in more socially charged decisions.

We should mention that often in sequential sampling models it is convention to fix the threshold separation to be constant between trials. We did not do this for two reasons. First, it is also commonly assumed that the response criterion in SDT would not be adjusted systematically from trial to trial. However, that is exactly what is reported as occurring when SDT is fit to the data from the FPST (Correll et al., 2002, 2007, 2011). Given these findings, we felt it would be important to examine how aspects of the response process may change from trial to trial when a dynamic perspective of the decision process is taken. Second, just as we learned that time pressure may not have a singular effect on the decision process (Rae et al., 2014; Voss et al., 2004), it also seems pertinent to examine the effect of between-trial manipulations on other aspects of the decision process. As we have outlined, we think this opens up new questions both about motivation and about how people control their threshold.

### The (lack of an) effect of race on the start point

The DDM also helps identify what is *not* responsible for the race bias in the FPST. In our data, the bias is not due to participants being “trigger happy” in the presence of Black targets. At least in the current design of the FPST, this is clear from the lack of difference in the relative starting points for Black and White targets. This result also speaks against the hypothesis that the stereotypical race response is the first response to arrive and bias the decision maker in the decision process (Payne, 2001, 2006; Payne & Bishara, 2009). Instead, the stereotypical association at least for novice young adults appears to shape the evidence accumulated online, as the difference in drift rates indicates. It is worth noting that different task designs might be more or less conducive to obtaining starting biases. For instance, a bias in the relative starting point may be more likely if the participant knows the race of the target on the upcoming trial in advance, as is typically the case when a police officer responds to a call. This point highlights the critical role of the design of the FPST for making inferences about the behavior of real-world decision makers, and the need for researchers to more closely match the decision landscape of laboratory decisions with that of real-world situations (James, Vila, & Daratha, 2013; James, Klinger, & Vila, 2014).

### The effect of context on the decision process

We also used three of our studies to probe how changes in context impacted the effect of race and the decision process in general. Correll et al. (2011) reported that the contexts or neighborhoods moderated the effect of race on the decision process, with participants setting lower criteria for dangerous neighborhoods regardless of the race of the target. This result was interpreted as showing that cues such as the level of danger of a neighborhood may create a predisposition to shoot in the FPST that apparently can wipe out the effect of race. Our results with the DDM offer a different account. First, the context never credibly impacted the effect of race on the drift rates. Second, changes in context had different effects across studies, impacting the threshold separation (Study 2), increasing drift rates towards the correct responses (Study 3), or increasing drift rates towards shooting for non-gun objects (Study 4). Taken together, these effects speak against a moderating role of context on the effect of race—and any consistent effect of context on the decision process in general. We suggest that part of the difficulty here is that the context, by definition, is not focal to the task and thus lends itself to different interpretations depending on how it is manipulated and what other variables are varied around it. In comparison, our analyses indicate that the effect of race on the decision process is quite consistent.

### Other applications of the DDM to the FPST

We are not the first to suggest that the DDM or a related sequential sampling model may provide a viable alternative to explaining data from the FPST (Correll et al., 2015) or similar tasks (Klauer & Voss, 2008). Correll et al. (2015) also found that race impacts the strength of the evidence accumulated in the FPST, with participants accumulating stronger evidence towards shooting Black targets than White targets. Yet this article goes substantially beyond those results in several ways. One is that due to the structure of the data, we developed and tested a Bayesian hierarchical model for the DDM, as opposed to fitting the model at the individual level using maximum likelihood. As we discussed earlier, this framework allows for more accurate estimates of the parameters at the individual and group level. It might also rectify a finding from Correll et al. (2015) that does not seem quite right: Although the point structure of the FPST encourages a bias to shoot Correll et al. (2015) reported an overall starting bias of *less than* .5, indicating that participants showed a tendency to not shoot. Yet *a priori* the starting bias should be greater than .5. Our analyses showed the predicted positive starting bias toward shooting across all four studies.^15^

As should be clear, the Bayesian hierarchical model also allowed us to ask questions about the effect of race that are more difficult to address using approaches that only fit the model at the individual level. For instance, we found some evidence that participants sometimes set larger threshold separations for Black than for White targets. Correll et al. (2015), presumably due to limited number of observations per subject, had to fix the threshold separation to be equal between race conditions a priori. Another way we go beyond past studies is that we were able to examine how other factors, such as response window, context, and discriminability, impact the decision process during the FPST. Rather surprisingly, these factors had little to no impact on the effect of race on the drift rates, reinforcing past results that speak to the power of racial stereotypes (Bargh, 1999).

Many studies in recent years have claimed to demonstrate flexibility and malleability of stereotype activation due to context changes (see, e.g., Blair, 2002; Blair, Ma, & Lenton, 2001; Casper, Rothermund, & Wentura, 2010; Castelli & Tomelleri, 2008; Sinclair, Lowery, Hardin, & Colangelo, 2005; Wittenbrink et al., 2001). However, there has also been criticism of these conclusions (e.g., Bargh, 1999). It is important to note that, in all studies, stereotype activation is assessed by comparing average response times across various conditions. The key assumption is that slower responses to, say, certain stereotype words reflect weaker activation of those stereotype terms. However, the modeling approach advocated in this article suggests a different possibility: Rather than stereotypes or their activation changing, changes in some other decision parameter could lead to slower response times, even while the stereotype and its activation remains constant (as indicated by the drift rates).

More generally, past uses of the DDM in the social literature have tended to treat it as a vehicle for revealing something important about a specific task—e.g., the FPST—and as a method interchangeable with other methods (e.g., SDT, eye-tracking methods). Besides demonstrating that the DDM is not simply interchangeable with SDT, we have shown that it can tell us something about social cognitive processes in general and that—through its ability to account for data often considered consistent with a dual process with a single sequential sampling process—the DDM is important in its own right. Hence, we attempt a more general statement about cognitive process and models than has been accomplished in the past.

### Implications for the decision to use deadly force by police officers

A major motivation for this research was to begin to understand the split-second decision that police officers have to make on whether or not to use deadly force, and how the race of the target might impact that decision. There are many limitations with our studies that impede our ability to make strong statements to how this decision plays out in the field in dangerous situations. Obviously the participants were never in danger and the scene was on the computer monitor. Another limitation is the decision itself. The decision in the FPST is not the same decision that police officers face in the field. In the FPST, participants are only supposed to shoot if the target is holding a gun. In the field, police officers must continuously assess the level of threat and the presense of a gun is only one factor. Moreover, in the FPST, participants have to explicitly choose between “Shoot” or “Don’t Shoot.” The real shoot decision arguably lacks an explicit “Don’t Shoot” option. Does this mean a qualitatively different decision process is used? The answer at this point is unknown. However, the single choice option of “Shoot” is parallel to what experimental psychologists call a Go/No-Go procedure (Donders, 1969/1868) (see also Logan & Cowan, 1984; Verbruggen & Logan, 2008). During this procedure participants are given two options and participants must respond to one of the choices (“Go” or “Shoot”) but must withhold a response to the other alternative (“No-Go” or “Don’t Shoot”). This response can also be modeled with a drift-diffusion process with only a single boundary, what is called a shifted Wald distribution (Wald, 1947). In model comparisons, however, a better model of the Go/No-Go procedure is sometimes the two-boundary model (Gomez, Perea, & Ratcliff,^2007^).

Another limitation is that our samples were all undergraduate students and not police officers. This raises the question whether the same effects be observed on police officers’ decisions to shoot? We believe that the DDM may be able to capture the complex pattern of results observed in police officers. Although trained officers often show similar response time biases, they typically do not show biases in error rates, shooting unarmed Black and White individuals at roughly similar rates, and sometimes showing reversals of the typical race effect (Correll et al., 2007; James et al., 2013, 2014; Plant & Peruche, 2005; Sim et al., 2013). Based on the response time data, we would expect to see different drift rates for Black and for White targets. The lack of a race effect on error rates in this population is likely due to police officers showing higher drift rates on average, meaning they have greater processing efficiency in extracting the relevant information from the scene. This increase would make their biases in error rates less pronounced. The advantage of the Bayesian hierarchical DDM is that it provides a means to measure and test for these biases at the process level, even if they are not apparent at the behavioral level. Our current work with young adult participants establishes the viability of the DDM to go forward with this important next step.

The use of the DDM to understand race biases can extend beyond simply characterizing race biases. By identifying how the race of the target impacts the process, different training approaches can be identified. Our results suggest that the race bias apparent in the FPST is due to participants processing the object and the race of the target holding the object interdependently. Thus, although one might expect that advising people to slow down and collect more information would counteract biases, the DDM indicates that it will not wipe out the race bias. This is because the race bias is located in the information accumulated over time. All else equal, collecting more information for all targets will reduce bias in errors. However, this collect-more-information strategy will not address the race bias itself which is in the evidence accumulation. This is a problem because in real-world circumstances, waiting long enough to avoid errors is often not an option. One solution, which was sometimes taken by our participants, is to increase the threshold separation for Black targets, thus offsetting the bias for shooting unarmed targets. However, even here, the bias will still be in the evidence and this asymmetric increase in threshold for Black targets will not address the bias in the errors for armed targets. Another solution may be to offset the bias in evidence accumulation via changes in the initial start point, such as by changing incentives or expectations to bias individuals away from shooting Black targets. A final possibility is to change how individuals process the evidence itself—perhaps by training them to focus only on relevant aspects of the situation, namely, the object that the target is holding. These are all possible solutions that our model identifies as a means to counteract this problem of allowing race to influence the decision to shoot. We must reiterate that these predictions are derived from results with young adults completing a much simplified version of the task. Before these training procedures are investigated further the next important step is to investigate how our results generalize to police officers in more realistic environments.

## Conclusion

Police officers sometimes have to make critical decisions on whether or not to use deadly force under uncertainty and time pressure. A rich set of empirical results accumulated using the FPST show that racial stereotypes systematically bias the decision to shoot. Past theoretical accounts have attributed this effect to the role of automatic stereotype processes or to a response bias. However, neither of these accounts give a satisfactory explanations of all the choice and response time data obtained using the FPST. We have shown that the DDM gives a parsimonious, single process account of the decision to shoot in the FPST. More importantly, it shows how different components of the process interact: we found that racial stereotypes biased the information used to make the decision, while at the same time participants appeared to counteract the bias by collecting more evidence for Black than White targets. We believe that this ability of the DDM to quantitatively characterize multiple aspects of the decision process—controlled and automatic—represents a significant advance in the study of social cognitive processes.

## Electronic supplementary material

Below is the link to the electronic supplementary material.

(PDF 1.02 MB)

## Footnotes

1 Measuring the degree of bias based on actual shootings is not straightforward due to questions about the biases and reliability of the reports. In general, however, reports indicate that the proportion of Blacks relative to Whites being shot by police is greater than would be expected based on population proportions alone (Brown & Langan, 2001; Geller, 1982; Geller & Scott, 1992; Jacobs & O’Brien, 1998; Meyer, 1980; Robin, 1963; Ross, 2015; Smith, 2004). Recent analyses show that a racial bias in the use of force is still present after controlling for arrest rates, but if one conditions solely on the use of lethal force then, on average, no statistically reliable racial disparity is found (Goff, Lloyd, Gelle, Raphael, & Glaser, 2016), or perhaps the opposite racial disparity is found (Cesario, Johnson, & Terrill, 2017).

2 Another model that has been used is the process dissociation model (Payne, 2005, 2006; Plant et al., 2005). Although the process dissociation model and SDT models have different conceptual interpretations, they reparameterize the choice data in a similar manner and consequently their parameters will often be perfectly correlated. For instance, the measure of control in the process dissociation model and the measure of sensitivity in SDT are both a function of the difference between the hit and false alarm rates and are thus perfectly positively related. A similar relationship holds between the measure of automaticity in the process dissociation model and the response criterion in SDT. Thus, the limitations we identify with SDT’s account of the decision to shoot also apply to the process dissociation model.

3 The noise in each sample is determined by the parameter *σ* ^2^ called the drift coefficient. For our purposes it is set to 1.0. This is because the drift coefficient is a scaling parameter; that is, if the parameter were doubled, other parameters of the model could be doubled to produce exactly the same predictions. However, with multiple conditions we can estimate how this noise parameter changes and potentially obtain better fits and more accurate parameter estimates (Donkin, Brown, & Heathcote, 2009).

4 In principle, each object could have a different drift rate, modeling the variability between objects (e.g., different guns, different non-gun objects). One way to do this is to model the stimuli as random effects rather than fixed effects, which would perhaps be more appropriate throughout experimental psychology (see Clark 1973; Judd, Westfall, & Kenny, 2012). Although the Bayesian modeling framework we introduce later allows this, for simplicity, we do not model the variability between stimuli and instead focus on modeling the systematic variability between gun and non-gun trials.

5 DDMs are the continuous-time versions of random walks.

6 We thank Josh Correll for sharing these data.

7 In Studies 1, 2, and 3, there was no reliable effect (interaction or main effect) of foreperiod duration on choice accuracy or mean response times. Study 4 did not record the foreperiod duration used for each trial. Thus, for all analyses we collapse across this factor.

8 This truncation was done for theoretical reasons as *β* must fall between 0 and 1, and for practical reasons as the estimation process becomes unstable with values close to 0 and 1. Thus, we set the upper limits away from these boundaries.

9 Using different priors, such as more diffuse normals, had minimal impact on the parameter estimates.

10 ANOVA analyses with response times were calculated using an inverse transformation of observed response times.

11 A SDT model that models different classes of stimuli rather than a single class of stimuli would also likely capture this effect (see, e.g., Glanzer & Adams, 1985).

12 Note that this parallels the results for White vs. Black targets.

13 To explore the possibility that distance from the screen was a confounding factor, we manipulated this variable within subjects. As it proved to have no effect, however, we collapsed across this variable in our analyses.

14 We use the term composite rather than meta-analysis as there is a clear dependency on the designs of the studies. Nevertheless, we believe there is value in synthesizing the data across these studies to give a sense of the total empirical support for the effect of race on the decision to shoot that can be had from these four studies.

15 Our Bayesian hierarchical DDM also provided a means to model the missing data caused by the non-recording of responses that fell outside the response window. It is unclear whether and how Correll et al. (2015) accounted for this censoring problem, which will also bias parameter estimates.
