# Supplementary material for: How race affects evidence accumulation during the decision to shoot
Source: Psychon Bull Rev. 2017 Oct 5;25(4):1301–30. doi: 10.3758/s13423-017-1369-6 (PMC6096652; doi:10.3758/s13423-017-1369-6)
Supplement: Supplementary file 1 — (PDF 1.02 MB) [file 13423_2017_1369_MOESM1_ESM.pdf]

Supplemental Material for How Race Impacts Evidence Accumulation During the Decision to Shoot

Timothy J. Pleskac*, Joseph Cesario** and David Johnson**

*Center for Adaptive Rationality, Max Planck Institute for Human Development, Berlin, Germany
**Department of Psychology, Michigan State University

June 22, 2017

Contents

1 Analysis of Variance Tables **3**
1.1 Study 1 . . . 3
1.2 Study 2 . . . 3
1.3 Study 3 . . . 4
1.4 Study 4 . . . 7
1.5 Composite Analysis . . . 7

2 Bayesian Model Estimation **8**
2.1 Hierarchical Bayesian Signal Detection Model . . . 9
2.1.1 Description of Model . . . 9
2.1.2 JAGS code . . . 11
2.2 Hierarchical Bayesian Drift Diffusion Model . . . 12
2.2.1 Description of model . . . 12
2.2.2 JAGS code . . . 13

3 Signal Detection Analysis **14**
3.1 Study 1 . . . 14
3.2 Study 2 . . . 15
3.3 Study 3 . . . 15
3.4 Study 4 . . . 17
3.5 Composite Analysis . . . 17

4 Parameter Recovery Analysis for the Hierarchical Drift Diffusion Model **18**

5 Posterior Predictive Checks for the Drift Diffusion Model **22**
5.1 Study 1 . . . 23
5.2 Study 2 . . . 25
5.3 Study 3 . . . 29
5.4 Study 4 . . . 35

6 Drift Diffusion Model Process Parameter Effects Tables **37**
6.1 Study 2 37
6.2 Study 3 38
6.3 Study 4 40
6.4 Composite Analysis 42

1 Analysis of Variance Tables

We list below the analysis of variance tables for the error rates and response times for correct choices for all four studies including the combined analysis. Analyses on the response times were conducted using the inverse of the RT ($1/RT$). All analyses were carried out using JASP version 0.8 Beta 5 (JASP Team, 2017). Sums of squares are Type III Sum of Squares.

As the studies were designed within the framework of Null Hypothesis Testing, we rely on p-values and estimates of effect sizes for the substantitive conclusions from the behavioral level analyses. However, we also report Bayes factors for each effect as a means of informing the interpretation and the degree of confidence one can have in the specific conclusion.

The reported Bayes factors are inclusion Bayes factors. Inclusion Bayes factors provide an estimate for the evidence for the particular effect combined across all the possible ANOVA models containing the effect (Rouder et al., 2016). The Bayes factors were estimated using JASP (JASP Team, 2017; Morey & Rouder, 2015). We use the notation BF_{10} to indicate Bayes factors indicating evidence in favor of the alternative hypothesis. Conventionally, Bayes factors between 1 and 3 are understood as weak evidence for the given hypothesis, 3 to 20 as positive, 20 to 100 as strong, and greater than 100 as very strong. Bayes factors less than 1 indicate evidence in favor of the other hypothesis (Raftery, 1995).

1.1 Study 1

Table S1: ANOVA Summary Table for Study 1 Error Rates

	Sum of Squares	df	Mean Square	F	p	η_p^2	BF_{10}
Object	0.014	1	0.014	6.260	.015	.102	4.131
Residual	0.127	55	0.002				
Race	0.018	1	0.018	7.262	.009	.117	8.797
Residual	0.139	55	0.003				
Object * Race	0.008	1	0.008	5.039	.029	.084	3.014
Residual	0.092	55	0.002				

Table S2: ANOVA Summary Table for Study 1 Correct Response Times

	Sum of Squares	df	Mean Square	F	p	η_p^2	BF_{10}
Object	2.446	1	2.446	349.038	$<.001$	.864	> 1000
Residual	0.385	55	0.007				
Race	0.001	1	0.001	0.601	.442	.011	> 1000
Residual	0.118	55	0.002				
Object * Race	0.162	1	0.162	75.445	$<.001$	.578	> 1000
Residual	0.118	55	0.002				

1.2 Study 2

Table S3: ANOVA Summary Table for Study 2 Error Rates

	Sum of Squares	df	Mean Square	F	p	η_p^2	BF_{10}
Context	0.005	1	0.005	0.194	.660	.002	0.0901
Residual	3.063	114	0.027				
Object	0.122	1	0.122	8.903	.003	.072	73.840
Object * Context	0.004	1	0.004	0.261	.610	.002	0.068
Residual	1.1563	114	0.014				
Race	0.010	1	0.010	2.107	.149	.018	0.397
Race * Context	9.399e-4	1	0.9.399e-4	0.190	.664	.002	0.033
Residual	0.564	114	0.005				
Object * Race	0.061	1	0.061	10.562	.002	.082	1.609
Object * Race * Context	0.021	1	0.021	3.685	.057	.029	0.022
Residual	0.657	114	0.006				

Table S4: ANOVA Summary Table for Study 2 Response Times in Correct Choices

	Sum of Squares	df	Mean Square	F	p	η_p^2	BF_{10}
Context	0.001	1	0.001	0.037	.849	.000	0.262
Residual	4.152	114	0.036				
Object	7.214	1	7.214	462.498	$< .001$	.802	> 1000
Object * Context	0.051	1	0.051	3.252	.074	.028	0.935
Residual	1.778	114	0.016				
Race	0.014	1	0.014	2.712	.102	.023	0.109
Race * Context	6.666e-4	1	6.666e-4	0.130	.719	.001	0.031
Residual	0.583	114	0.005				
Object * Race	0.008	1	0.008	1.892	.172	.016	0.095
Object * Race * Context	0.024	1	0.024	5.377	.022	.045	0.024
Residual	0.500	114	0.004				

1.3 Study 3

Table S5: ANOVA Summary Table for Study 3 Error Rates

	Sum of Squares	df	Mean Square	F	p	η_p^2	BF_{10}
Object	6.342e-4	1	6.342e-4	0.080	.779	.002	13.431
Residual	0.295	37	0.008				
Race	0.005	1	0.005	0.936	.340	.025	0.106
Residual	0.207	37	0.006				
Context	0.072	1	0.072	11.591	.002	.239	16.240
Residual	0.230	37	0.006				
Discrim.	0.019	1	0.019	5.289	.027	.125	18.698
Residual	0.130	37	0.004				
Object * Race	0.034	1	0.034	8.138	.007	.180	0.518
Residual	0.156	37	0.004				
Object * Context	0.011	1	0.011	2.452	.126	.062	0.368
Residual	0.167	37	0.005				
Object * Discrim.	0.106	1	0.106	18.841	<.001	.337	87.994
Residual	0.207	37	0.006				
Race * Context	1.978e-4	1	1.978e-4	0.035	.852	.001	0.064
Residual	0.206	37	0.006				
Race * Discrim.	0.005	1	0.005	1.164	.288	.031	0.106
Residual	0.174	37	0.005				
Context * Discrim.	0.013	1	0.013	4.530	.040	.109	0.408
Residual	0.110	37	0.003				
Object * Race * Context	6.996e-4	1	6.996e-4	0.010	.922	.000	0.018
Residual	0.264	37	0.007				
Object * Race * Discrim.	3.745e-4	1	3.745e-4	0.061	.807	.002	0.068
Residual	0.229	37	0.006				
Object * Context * Discrim.	0.012	1	0.012	3.924	.055	.096	0.224
Residual	0.110	37	0.003				
Race * Context * Discrim.	0.012	1	0.012	1.542	.222	.040	0.010
Residual	0.287	37	0.008				
Object * Race * Context * Discrim.	0.007	1	0.007	1.481	.231	.038	1.701e-4
Residual	0.183	37	0.005				

Table S6: ANOVA Summary Table for Study 3 Correct Response Times

	Sum of Squares	df	Mean Square	F	p	η_p^2	BF_{10}
Object	2.238	1	2.238	15.166	<.001	.291	> 1000
Residual	5.460	37	0.148				
Race	0.065	1	0.065	5.199	.028	.123	0.025
Residual	0.465	37	0.013				
Context	0.242	1	0.242	1.741	.195	.045	0.095
Residual	5.151	37	0.139				
Discrim.	0.052	1	0.052	3.719	.061	.091	0.025
Residual	0.515	37	0.014				
Object * Race	0.104	1	0.104	5.554	.024	.131	0.032
Residual	0.695	37	0.019				
Object * Context	0.194	1	0.194	1.423	.241	.037	0.155
Residual	5.049	37	0.136				
Object * Discrim.	0.176	1	0.176	10.721	.002	.225	0.048
Residual	0.607	37	0.016				
Race * Context	0.092	1	0.092	5.808	.021	.136	0.015
Residual	0.586	37	0.016				
Race * Discrim.	0.025	1	0.025	0.182	.672	.005	0.004
Residual	4.979	37	0.135				
Context * Discrim.	0.020	1	0.020	1.377	.248	.036	0.010
Residual	0.538	37	0.015				
Object * Race * Context	2.958e-4	1	2.958e-4	0.017	.897	.000	0.001
Residual	0.640	37	0.017				
Object * Race * Discrim.	0.207	1	0.207	1.579	.217	.041	0.001
Residual	4.861	37	0.131				
Object * Context * Discrim.	0.018	1	0.018	1.257	0.269	.033	0.001
Residual	0.542	37	0.015				
Race * Context * Discrim.	0.021	1	0.021	0.153	.698	.004	< .001
Residual	4.987	37	0.135				
Object * Race * Context * Discrim.	0.072	1	0.072	0.542	.466	.014	< .001
Residual	4.896	37	0.132				

1.4 Study 4

Table S7: ANOVA Summary Table for Study 4 Error Rates

	Sum of Squares	df	Mean Square	F	p	η_p^2	BF_{10}
Race	2.611e-4	1	2.611e-4	0.070	.791	.001	6.610
Residual	0.397	107	0.004				
Context	0.335	1	0.335	56.732	$< .001$	.346	> 1000
Residual	0.633	107	0.006				
Object	1.086	1	1.086	28.914	$< .001$	.213	> 1000
Residual	4.017	107	0.038				
Race * Context	0.005	1	0.005	1.292	.258	.012	0.332
Residual	0.374	107	0.003				
Race*Object	0.143	1	0.143	37.938	$< .001$	.262	36.765
Residual	0.404	107	0.004				
Context*Object	0.036	1	0.036	7.419	.008	.065	1.705
Residual	0.519	107	0.005				
Object * Race * Context	0.009	1	0.009	2.045	.156	.019	0.200
Residual	0.450	107	0.004				

Table S8: ANOVA Summary Table for Study 4 Response Times in Correct Choices

	Sum of Squares	df	Mean Square	F	p	η_p^2	BF_{10}
Race	5.336e-4	1	5.336e-4	0.012	.911	.000	0.037
Residual	4.580	107	0.043				
Context	2.937	1	2.937	3.087	.082	.028	8.055
Residual	101.795	107	0.951				
Object	5.753	1	5.753	44.759	$< .001$	.295	> 1000
Residual	13.754	107	0.129				
Race * Context	0.184	1	0.184	1.329	.252	.012	0.026
Residual	14.813	107	0.138				
Race*Object	0.274	1	0.274	1.570	.213	.014	0.031
Residual	18.683	107	0.175				
Context*Object	0.593	1	0.593	2.718	.102	.025	0.675
Residual	23.331	107	0.218				
Object * Race * Context	0.115	1	0.115	0.650	.422	.006	0.001
Residual	18.890	107	0.177				

1.5 Composite Analysis

We also included the ANOVAs on the data used in the composite analysis. This analysis examined the race bias effect across all four studies in the common conditions of the FPST (i.e., the neutral context with a non-blurred object). Experiment was entered into the analysis as a between subjects factor with four levels.

Table S9: ANOVA Summary Table for Composite Analysis of Error Rates

	Sum of Squares	df	Mean Square	F	p	η_p^2	BF_{10}
Object	0.250	1	0.250	20.218	<.001	.073	>1000
Object * Study	0.085	3	0.028	2.2997	.078	.026	10.228
Residual	3.169	256	0.012				
Race	9.802e-5	1	9.802e-5	0.026	.873	.000	21.283
Race * Study	0.049	3	0.016	4.262	.0006	.048	1.926
Residual	0.978	256	0.004				
Object * Race	0.079	1	0.079	18.605	<.001	.068	119.209
Object * Race * Study	0.089	3	0.030	6.980	<.001	.076	10.670
Residual	1.090	256	0.004				

Table S10: ANOVA Summary Table for Composite Analysis of Response Times of Correct Choices

	Sum of Squares	df	Mean Square	F	p	η_p^2	BF_{10}
Object	11.395	1	11.395	1055.289	<.001	.805	>1000
Object * Study	0.167	3	0.056	5.156	.002	.057	>1000
Residual	2.764	256	0.011				
Race	0.020	1	0.020	4.709	.031	.018	23.57
Race * Study	0.041	3	0.014	3.151	.026	.036	26.36
Residual	1.109	256	0.004				
Object * Race	0.063	1	0.063	15.240	<.001	.056	122.89
Object * Race * Study	0.133	3	0.044	10.783	<.001	.112	196.60
Residual	1.051	256	0.004				

2 Bayesian Model Estimation

All of our analyses with SDT and the DDM were embedded within a hierarchical framework (Vandekerckhove et al., 2011; Wabersich & Vandekerckhove, 2014) and use Bayesian estimation techniques (Kruschke, 2014; Lee & Wagenmakers, 2013) to estimate the parameters (and the effects of the different conditions on the parameters). Uninformative priors were used for each parameter to let the data have maximal influence on the posterior estimates.

Our model estimation procedure was implemented with JAGS 3.4 using Matlab via matjags to interface with JAGS. The general JAGS code used is given below. The estimation of the SDT model used four parallel chains. Each chain consisted of 1000 burn-in steps (unrecorded samples to allow the chain to reach the reasonable parameter space) and 20,000 samples for a total of 80,000 samples.

The estimation of the drift diffusion model was a bit more computationally intensive. We used 32 chains each with 2000 burn-in steps and 2,500 samples for a total of 80,000 samples.

The chains were evaluated for the representativeness and accuracy using the procedures outlined by Kruschke (2014). Representativeness was evaluated using visual inspection of trace plots of the chains and density plots. All the chains at the group level were inspected visually with random samples of chains from the individual level. Representativeness was also evaluated numerically using the Gelman-Rubin statistic with the conventional heuristic that values of the Gelman-Rubin statistic

above 1.1 were worrisome. All the chains met these standards suggesting representativeness of the posterior distributions. Accuracy was evaluated by examining the autocorrelation and the effective sample size. The effective sample size estimates the sample size of the chain after accounting for the autocorrelation present in the samples. As a rough standard we sought to have approximately an effective sample size of approximately 10000. Our main focus in this paper was on comparing the group level mean estimates of the parameters. All of those distribution were at least greater than 8000 and most were above 10000.

2.1 Hierarchical Bayesian Signal Detection Model

2.1.1 Description of Model

Many published studies using the FPST employ signal detection theory (SDT) to analyze the choice data (e.g., Correll et al., 2002; Correll, Park, Judd, & Wittenbrink, 2007; Correll, Park, Judd, Wittenbrink, Sadler, & Keesee, 2007; Greenwald et al., 2003; Kenworthy et al., 2011; Sadler et al., 2012; Sim et al., 2013). According to this account, when participants are presented with a target they draw a sample of information relating to the presence of the gun. They compare this internal estimate of danger x against a criterion c, and if the value exceeds the criterion they choose "Shoot"; otherwise, they choose "Don't Shoot." The model assumes that the targets encountered vary on this danger dimension. The standard model used in the analyses assumes that the magnitudes are normally distributed, with trials in which non-gun objects are presented producing danger estimates that are normally distributed, with a mean of 0 and a standard deviation of 1. Trials in which gun objects are presented have a mean of d' and a standard deviation of 1. The parameter d' measures the degree to which the targets that are holding a gun have a higher average danger value.

The advantage of signal detection analysis is that it separates accuracy or sensitivity in discriminating between dangerous and neutral targets from properties of the response, such as the participants' goals or expectations at the time of the decision. Past analyses have shown that manipulations of race and context primarily impact the criterion c that participants use, suggesting that these manipulations primarily impact participants' expectations. In particular, participants tend to set a lower, more liberal criterion c for Black targets than for White targets.

We submitted the data from each of the four studies to a Bayesian signal detection analysis (Lee, 2008; Lee & Wagenmakers, 2013). The advantage of this approach is that the data can be modeled hierarchically to yield group-level and individual estimates. Figure S1 shows a graphical model of the hierarchical SDT model we used. The JAGS code for the model is given below.

The hierarchical structure means that each process parameter of the SDT model had a higher order group-level prior. For example, focusing on d', according to the model for each between-subject condition i^*, within subject condition i, subject j there was a different sensitivity, $d'_{i^*,i,j}$. Prior beliefs in the distribution of the values for each subject in each condition was represented with a (diffuse) normal distribution a mean $\mu^{d'}_{i^*,i}$ and precision τ_{i^*} (the inverse of the variance). We assume each subject is from the same population or group. Thus, part of our uncertainty can be isolated to uncertainty in what the sensitivity was at the group level as well as the precision. This uncertainty was modeled with group level distributions shown at the top of Figure S1. The precision parameters (τ) do not vary by within-subjects conditions. This approach was taken to model the within-subjects nature of the manipulation, by building a dependency between the conditions for subjects (Kruschke, 2014). Between-subjects manipulations (indexed by i^* in Figure S1) were structured so that both the mean and precision parameters varied between the conditions.

As Figure S1 shows, our prior beliefs in possible values of these hyperparameters were set to be normally distributed for the mean, and gamma distributed for the precision parameter. We used

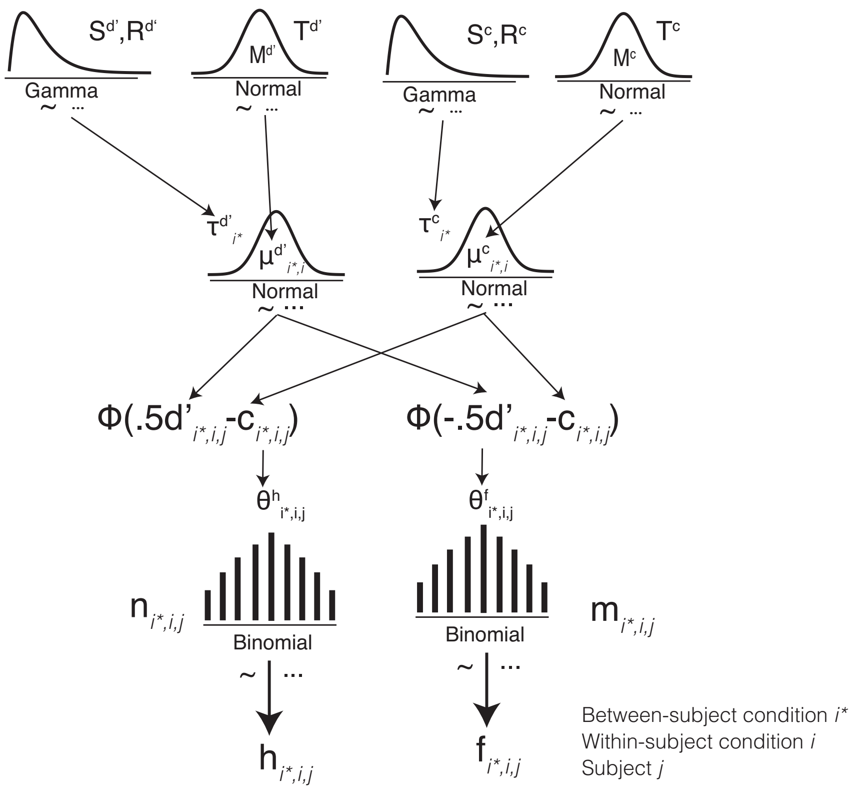

Figure S1: Diagram of the hierarchical signal detection model. The hth hit and fth false alarm for subject j in within-subject condition i, between-subject condition i^*, are each generated by binomial distribution.

diffuse priors on these hyperparameters so that the precision in our beliefs was low, letting the data have maximal influence on the posterior estimates. In a Bayesian analysis, we used the observed data to update our beliefs in these parameters, obtaining posterior distributions over their possible values. The posterior distributions reflect the degree of belief or degree of certainty associated with the possible values of the parameters after observing the data.

2.1.2 JAGS code

```
model {
#hyperpriors
for( i in 1 : nWCon) { #index for within conditions
  for (j in 1:nBCon) { # index for between condition,
  #need to remove between index if no between conditions
    muc[i,j] ~ dnorm(0,.001)
    mud[i,j] ~ dnorm(0,.001)
    mucPrior[i,j] ~ dnorm(0,.001)
    mudPrior[i,j] ~ dnorm(0,.001)
  }
}

for (j in 1 :nBCon) {# index for between condition,
#need to remove between index if no between conditions
    lambdac[j] ~ dgamma(.001,.001)
    lambdad[j] ~ dgamma(.001,.001)
    sigmac[j] <- 1/sqrt(lambdac[j])
    sigmad[j] <- 1/sqrt(lambdac[j])
}
for( i in 1 : nWCon) { #index for within conditions
    for (j in 1:nSub){#index for number of subjects
        #priors
        #reparameterization using equal var gaussian SDT
        c[i,j] ~ dnorm(muc[i,bCon[j]],lambdac[bCon[j]])
        d[i,j] ~ dnorm(mud[i,bCon[j]],lambdad[bCon[j]])
        thetah[i,j] <- phi(d[i,j]/2-c[i,j])
        thetaf[i,j] <- phi(-d[i,j]/2-c[i,j])
        h[i,j] ~ dbin( thetah[i,j], s[i,j])
        f[i,j] ~ dbin( thetaf[i,j], n[i,j])
        #posterior prediction
        hitPost[i,j] ~ dbin(thetah[i,j], s[i,j])
        faPost[i,j] ~ dbin(thetaf[i,j], n[i,j])
    }
}
}
```

The inputs to the model were as follows

h matrix of observed number of hits for each within subject condition, for each subject (i.e., Shoot, Gun trials) of size (No. Within x No. Subjects)

f vector of observed number of false alarms for each within subject condition, for each subject (i.e., Shoot, Non-Gun Trials) of size (No. Within x No. Subjects)

s vector of observed signal trials or each within subject condition, for each subject (i.e., Gun Trials) of size (No. Within x No. Subjects)

n vector of observed noise trials or each within subject condition, for each subject (i.e., Non-Gun Trials) of size (No. Within x No. Subjects)

bCon indicator vector of between subject condition

nWCon number of within subject conditions

nBCon number of between subject conditions

nSub number of subjects

2.2 Hierarchical Bayesian Drift Diffusion Model

2.2.1 Description of model

There are many different ways to parameterize the Hierarchical Bayesian DDM. We carried out several preliminary analyses in terms of identifying how to parameterize the DDM to model the data from the FPST (Heathcote et al., 2015). The model we used allowed the relative start point, threshold separation, drift rate, and non-decision time to vary as a function of race and all other experimental manipulations (e.g., context and discriminability), and only drift and non-decision time were allowed to vary as a function of object type. Please see the main text for a description of how and why we came to this specification.

Note we also examined whether including trial-by-trial variability in drift rate, relative start point, and non-decision time, improves the fit of the model (Ratcliff, 1978; Ratcliff & Rouder, 1998; Ratcliff & Smith, 2004). In Study 1 and Study 2 this model failed to converge, likely due to such few observations per subject (only 80 to 100 observations per subject). In Study 3 and 4, we were able to estimate the model, and the fit improves. But, the conclusions reported in the paper remain the same. For parsimony and for consistency in the paper we rely on the model without trial-by-trial variability in the parameters

We also conducted a parameter recovery analysis of the hierarchical drift diffusion model. A summary of the analysis is provided below. Briefly, for the standard experimental design of the FPST where $N = 50$ participants complete $n = 100$ trials our model recovery analysis shows that we can accurately and reliably recover the parameters of the hierarchical DDM. We also confirmed that changes in the relative start point, drift rate, and threshold separation, were accurately detected by the model.

One challenge that arose in modeling the data from Study 1 and 2 is that the data were censored above the response deadline (i.e., the observed response and response time were not recorded for trials in which the response was made outside the response window). This is a problem for the DDM and any model of the distribution of response times: If censoring is not accounted for, the distribution of response times will appear faster than it is, which will in turn impact the parameter estimates. The Bayesian approach makes it possible to build censoring directly into the model (Kruschke, 2014, p. 730). The method models the probability of the observed time falling beyond the response deadline. It does this by imputing a random value generated from the model and the credible parameter values at that step in the chain. Importantly, the imputed value must fall beyond the response deadline.

The code below shows how censoring was modeled within the hierarchical DDM. The model requires the specification of the intervals in which the data were censored or not. For example, in Study 1, responses longer than 0.63 s were not recorded. This means for the JAGS dwiener distribution that data below -0.63 and above 0.63 were not recorded. These two thresholds define three intervals. The first interval (bin = 0 in the code) identifies values below -0.63 that were censored. The second interval (bin = 1) identifies responses that were recorded. The third interval (bin = 2) identifies responses above 0.63 that were censored. JAGS uses the thresholds and their associated bins to impute the values of the appropriate bin. One challenge in this approach is that both the observed response and the response times were censored. To overcome this obstacle, we inferred the responses (i.e., "Shoot" or "Don't Shoot") on these trials from the observed relative frequency of these responses for gun and non-gun objects for each subject, collapsing across the conditions.

2.2.2 JAGS code

```
model {
#hyperpriors
for (j in 1:nBtwn) { #between sub index
#remove between index completely if no btwn condtion
   for (i in 1:nWConObject){ #within sub index w object
       muDelta[i,j] ~ dunif(-5,5)
       muNDT[i,j] ~ dunif(.001,1)
       #sample hyperpriors for inference tests
       muDeltaPrior[i,j] ~ dunif(-5,5)
       muNDTPrior[i,j] ~ dunif(.001,1)
    }
}
for (j in 1:nBtwn) { #between sub condition
#remove between index completely if no btwn condtion
    for (i in 1:nWCon){ #within sub condition wo object
       muBeta[i,j] ~ dunif(.1,.9)
       muBetaPrior[i,j] ~ dunif(.1,.9)
       muAlpha[i,j]  ~ dunif(.1,5)
       muAlphaPrior[i,j]  ~ dunif(.1,5)
    }
}
for (j in 1:nBtwn) { #between sub index
#remove between index completely if no btwn condtion
    tauDelta[j] ~ dgamma(.001, .001)
    tauBeta[j] ~ dgamma(.001, .001)
    tauAlpha[j] ~ dgamma(.001, .001)
    tauNDT[j] ~ dgamma(.001, .001)
}
#PRIORS
for (j in 1:nSub){ #subject index
    for (i in 1:nWCon){ #within con index wo object
        beta[i,j] ~ dnorm( muBeta[i,btwnCon[j]] , tauBeta[btwnCon[j]] ) T(.1,.9)
        alpha[i,j]  ~ dnorm( muAlpha[i,btwnCon[j]] , tauAlpha[btwnCon[j]] ) T(.1,5)
    }
    for (i in 1:nWConObject){ #within con index w object
        delta[i,j] ~ dnorm( muDelta[i,btwnCon[j]] , tauDelta[btwnCon[j]] ) T(-5,5)
        ndt[i,j] ~ dnorm( muNDT[i,btwnCon[j]] , tauNDT[btwnCon[j]] ) T(.001,1)
}}


for( i in 1 : nData ) {
    ybin[i] ~ dinterval( y[i] , censorLimitVec[i,] )
    y[i] ~ dwiener( alpha[wCon[i],sub[i]], ndt[wConObject[i],sub[i]], beta[wCon[i],
        ↪ sub[i]], delta[wConObject[i],sub[i]])
    #prey[i] ~ dwiener( alpha[wCon[i],sub[i]], ndt[wConObject[i],sub[i]], beta[wCon[i
        ↪ ],sub[i]], delta[wConObject[i],sub[i]])
    #use prey for the posterior predictions of the rts
}
}
```

The inputs to the model were as follows

y vector of observed response times (in seconds) coded as positive if trial was a 'Shoot' trial, negative if trial was 'Don't Shoot', and NaN if response fell outside response window.

nData number of observations

wConObject vector indicating the within subject condition including object for each trial

nWConObject number of within subject conditions including object

wCon vector indicating the within subject condition (excluding object) for each trial

nWCon number of within subject conditions (excluding object)

btwnCon indicator vector of between subject condition

nBtwn number of between subject conditions

sub vector indicating subject for each trial

nSub number of subjects

ybin vector indicating if the trial was censored and what bin the trial was in. The first interval (bin = 0 in the code) identifies values that were a 'Don't Shoot' and were less than $-RW$ where RW is the response window time in seconds. The second interval (bin = 1) identifies responses that were recorded. The third interval (bin = 2) identifies responses greater than RW.

censorLimitVec A matrix of size No. of trials x 2 with the response window values, e.g., for one trial this would be $[-RW, RW]$.

3 Signal Detection Analysis

3.1 Study 1

Table S11 summarizes the posterior group-level estimates of parameters of the model for Study 1. Given past results showing no effect with the 850 ms response deadline, we did not expect to observe any difference in the response criterion in this study. Consistent with this prediction, no credible effect of race was found on the response criterion (Table S12). One unexpected effect was that there was a credible effect of race on sensitivity, with participants having greater sensitivity for Black targets than for White targets. (though see also Ma et al., 2013; Sadler et al., 2012; Sim et al., 2013).

Table S11: Study 1 Posterior Means and 95% HDI (in Brackets) for the Group-Level Parameter Estimates of the Signal Detection Model

	Sensitivity $\mu^{d'}$	Criterion μ^c
White	3.39 [3.14, 3.64]	-0.15 $[-0.24, -0.06]$
Black	3.81 [3.54, 4.09]	-0.03 $[-0.12, 0.07]$

Table S12: Summary of Standardized Effects of Race on Group-Level $\mu^{d'}$ and μ^c for Study 1

		95% HDI		
Factor	Mean	Lower	Upper	Prop <0
Sensitivity $\mu^{d'}$	0.65	0.09	1.23	.009
Criterion μ^c	1.64	-0.38	4.75	.03

3.2 Study 2

Table S13 summarizes the posterior group-level estimates of the Bayesian SDT model for Study 2. Table S14 and S15 summarizes the effect of race, context, and their interaction on the group-level estimates of sensitivity and response criterions. Consistent with past studies, there was no credible effect of race, context, or an interaction on group-level sensitivity. Overall, participants did set a lower criterion for Black targets. Consistent with Correll et al. (2011), the effect of race on criterion was driven largely by the neutral contexts: Participants set a lower criterion for Blacks than for Whites in the neutral condition ($M = -0.17$ $[-0.28, -0.05]$), but a similar criterion for both target types in the dangerous condition ($M = -0.07$ $[-0.19, 0.06]$).

Table S13: Study 2 Posterior Means and 95% HDI (in Brackets) for the Group-Level Parameter Estimates of the Signal Detection Model

Race	Context	Sensitivity $\mu^{d'}$	Criterion μ^c
White	Neutral	2.31 [2.07, 2.54]	0.03 $[-0.05, 0.11]$
Black	Neutral	2.46 [2.22, 2.71]	-0.14 $[-0.23, -0.06]$
White	Dangerous	2.40 [2.18, 2.62]	-0.06 $[-0.14, 0.03]$
Black	Dangerous	2.47 [2.25, 2.69]	-0.12 $[-0.21, -0.03]$

Table S14: Summary of Standardized Effects of Race and Context on Group-Level $\mu^{d'}$ for Study 2

		95% HDI		
Factor	Mean	Lower	Upper	Prop <0
Race	0.18	-0.17	0.52	.16
Context	0.07	-0.28	0.43	.34
Race * Context	0.06	-0.28	0.41	.36

Table S15: Summary of Effects of Race and Context on the Group-Level Criterion μ^c for Study 2

		95% HDI		
Factor	Mean	Lower	Upper	Prop <0
Race	-0.71	-1.32	-0.14	> .99
Context	-0.18	-0.71	0.35	.75
Race * Context	-0.31	-0.84	0.22	.88

3.3 Study 3

Table S16 lists the group-level mean sensitivity ($\mu^{d'}$) and criterion (μ^c) estimates. Table S17 and S18 summarizes the effects of the manipulations on the group-level estimates. As in past studies, criteria were lower for Black targets, but there was no effect of race on sensitivity. In this study, there was no credible effect of context, neither was there an interaction with context on the response criteria. However, participants did show increased sensitivity in the dangerous conditions.

In terms of discriminability, the criterion estimates were larger when the objects were blurred ($M = 0.07$ $[0.02, 0.11]$) than when they were clear ($M = -0.08$ $[-0.13, -0.03]$) (see Table S16). There was no credible difference between blurred and non-blurred objects in terms of sensitivity to shoot. The effect of discriminability on the decision criterion highlights the difficulty that the SDT model has in properly characterizing this property. This is due to the fact that the non-gun objects provided some signal for the shoot decision.

Table S16: Study 3 Posterior Means and 95% HDI (Brackets) for the Group-Level Parameter Estimates of the Signal Detection Model

Race	Context	Discriminability	Sensitivity $\mu^{d'}$	Criterion μ^c
White	Neutral	Clear	2.77 [2.43, 3.10]	-0.02 $[-0.12, 0.08]$
Black	Neutral	Clear	2.54 [2.22, 2.88]	-0.13 $[-0.22, -0.04]$
White	Dangerous	Clear	2.88 [2.53, 3.22]	-0.05 $[-0.15, 0.05]$
Black	Dangerous	Clear	2.83 [2.50, 3.18]	-0.11 $[-0.21, -0.01]$
White	Neutral	Blurred	2.41 [2.08, 2.74]	0.13 [0.05, 0.22]
Black	Neutral	Blurred	2.48 [2.16, 2.82]	0.09 $[-0.01, 0.18]$
White	Dangerous	Blurred	2.84 [2.51, 3.18]	0.10 [0.00, 0.20]
Black	Dangerous	Blurred	2.75 [2.41, 3.08]	-0.06 $[-0.15, 0.04]$

Table S17: Summary of Standardized Effects of Race and Context on Group-Level $\mu^{d'}$ for Study 3

		95% HDI		
Factor	Mean	Lower	Upper	Prop <0
Race	-0.08	-0.36	0.19	.73
Context	0.32	0.04	0.59	.01
Discrim.	-0.16	-0.43	0.12	.87
Race * Context	0.00	-0.28	0.27	.49
Race * Discrim.	-0.07	-0.35	0.20	.70
Context * Discrim.	-0.09	-0.36	0.19	.73
Race * Context * Discrim.	-0.10	-0.38	0.18	.76

Table S18: Summary of Standardized Effects of Race and Context on Group-Level μ^c for Study 3

		95% HDI		
Factor	Mean	Lower	Upper	Prop <0
Race	-0.56	-1.82	-0.46	.88
Context	-0.64	-1.98	0.39	.91
Discrim.	2.05	0.46	4.52	0
Race * Context	0.211	-0.81	1.33	.33
Race * Discrim.	0.10	-0.93	1.21	.43
Context * Discrim.	0.59	-0.44	1.91	.11
Race * Context * Discrim.	-0.56	-1.82	0.46	.88

3.4 Study 4

Table S19 lists the group-level mean sensitivity ($\mu^{d'}$) and criterion (μ^{c}) estimates. Table S20 and S21 summarizes the effects of the manipulations on the group-level estimates. The criterion was lower for Black targets, but there was no effect of race on sensitivity. There was also no credible effect of context or interaction with context or sensitivity on the response criterion.

Table S19: Study 4 Posterior Means and 95% HDI (in Brackets) for the Group-Level Parameter Estimates of the Signal Detection Model

Race	Context	Sensitivity $\mu^{d'}$	Criterion μ^{c}
White	Neutral	1.49 [1.37, 1.62]	-0.03 [$-0.08, 0.01$]
Black	Neutral	1.47 [1.35, 1.59]	-0.14 [$-0.19, -0.09$]
White	Dangerous	1.20 [1.08, 1.32]	-0.10 [$-0.14, -0.05$]
Black	Dangerous	1.24 [1.12, 1.36]	-0.16 [$-0.21, -0.11$]

Table S20: Summary of Standardized Effects of Race and Context on Group-Level $\mu^{d'}$ for Study 4

		95% HDI		
Factor	Mean	Lower	Upper	Prop <0
Race	0.010	-0.208	0.224	.46
Context	-0.461	-0.678	0.239	> .99
Race * Context	-0.056	-0.272	0.161	.69

Table S21: Summary of Standardized Effects of Race and Context on the Group-Level Criterion μ^{c} for Study 4

		95% HDI		
Factor	Mean	Lower	Upper	Prop <0
Race	-0.447	-0.132	-0.038	> .99
Context	-0.212	-0.461	0.027	.96
Race * Context	-0.122	-0.359	0.130	.84

3.5 Composite Analysis

We also fit the hierarchical signal detection model to the data used in the composite analysis. We report the effects tables below. For ease of interpretation we report the effects in terms of response window instead of Study. Table S22 shows that there was a credible increase in sensitivity as the response window was increased across the studies. Table S23 shows that across experiments participants tended to set a lower decision criterion for Black than White targets though the HDI does overlap with 0. This is consistent with the interaction between race and response window where, if you recall, in Study 1 participants did not show a credible effect of race on the decision criterion (Table S12). This result is understood as the result of the larger response window in Study 1, and

illustrates the limitation in modeling the decision to shoot as a signal detection process.

Table S22: Summary of Standardized Effects of Race and Context on Group-Level $\mu^{d'}$ for Composite Analysis

		95% HDI		
Factor	Mean	Lower	Upper	Prop <0
Race	0.128	-0.121	0.377	.16
Window	1.876	1.577	2.174	0
Race * Window	0.028	-0.224	0.278	.41

Table S23: Summary of Standardized Effects of Race and Context on the Group-Level Criterion μ^{c} for Composite Analysis

		95% HDI		
Factor	Mean	Lower	Upper	Prop <0
Race	-0.365	-0.715	0.001	.98
Window	-0.081	-0.436	0.279	.67
Race * Window	0.429	0.068	0.788	.01

4 Parameter Recovery Analysis for the Hierarchical Drift Diffusion Model

We conducted a set of parameter recovery analyses using the Hierarchical Drift Diffusion Model. Here we summarize the results from three different analyses: one where the race bias was in the group level starting point, an analysis where the race bias was in the group level drift rates, and an analysis where the race bias was in the drift rates and larger thresholds for Black targets. For each analysis, we generated data at the experiment level. Each experiment had $N = 50$ simulated participants with $n = 100$ trials per participant (i.e., 25 trials per condition). This is the typical dataset size used with the First Person Shooter Task, like in Study 1.

We simulated datasets with several different parameter values, but the ones reported below come from the composite analysis reported in the main table collapsing across all four experiments and race. The group level means and standard deviations (the square root of the inverse of the precision parameter) used to generate the data are given in Table S24. Without an *a priori* estimate of the effect size for race we used the estimate from Study 1. The difference in drift rates for armed Black and White targets was 0.62. Accounting for the precision in the group level drift rates, using the estimated parameters from the composite analysis this difference corresponds to a $d = \frac{\mu^{\delta}_{Black} - \mu^{\delta}_{White}}{\sqrt{1/\tau^{\delta}}} \approx 1.00$. We used this estimate to generate a race effect in the start point, the drift rates, and/or threshold separations. The data were generated using a random walk approximation of the stochastic differential equation of a drift diffusion process where each time step in the walk was set to take $0.0001s$ (Tuerlinckx et al., 2001).

After simulating a dataset, we then fit the DDM used in the main analyses of the paper to the data. This model allowed the group level mean relative start point μ^{β} and threshold separation μ^{α} to vary between the race conditions and allowed the the group level mean drift rates μ^{δ} and non-decision time μ^{NDT} to vary between race and object conditions. Fitting the model was computationally intensive so we aimed to have at least 50 simulated datasets per model analysis. Due to occasional datasets resulting in non-converging model fits (about 1 in 10), we actually simulated 60 datasets. Using Matlab and JAGS 3.4.0 on a Linux machine with Intel Xeon E5-2670 with 32 cores with processing speed of 2.6 GHZ to simulate and estimate the model (see Bayesian Model Estimation for more details), each analysis took approximately 9 hours to complete.

Table S24 lists the average posterior mean estimates and the average values of the 95% HDI for each of the generating parameters. As one can see the model recovery does an excellent job in recovering the parameters.

We also used the parameter recovery analysis to estimate the proportion of times the hierarchical DDM analysis would identify a credible race effect, a kind of power analysis (Kruschke, 2010, 2013). Table S25 lists for each parameter the average difference between the race conditions, the average estimates of the corresponding 95% HDI, and the proportion of times a credible effect of race was identified using a 95% HDI. The model analysis shows that the DDM (a) never incorrectly identifies a race effect in a different process parameter; and (b) does a good job of correctly identify credible effect.

One will note that across the four studies the effect of race on the drift rates does get smaller. This raises the question how this will impact the inferences. This is a difficult and computationally intensive question to answer. However, we sought to at least get some insight here, though certainly more work with a larger number of simulations is needed. Using the estimates from the composite analysis, supposing the effect size drops to $d = 0.5$ and nothing else is changed (i.e., sample size of $N = 50$ participants who each complete $n = 100$ trials total i.e. 25 trials per condition) our simulation show that using a 95% HDI the proportion of times a credible effect is found in any of the parameters approaches 50 to 70%. If the number of participants is increased to $N = 100$ participants then the proportion of times a credible effect goes to $> 85\%$ for drift rates and threshold separations, and $> 60\%$ for relative start points. Another option is to also increase the number of trials, which has a similar effect. For instance, for a medium size effect using 160 trials (as in our Study 4) the proportion of times a credible effect is found goes to $> 85\%$ for drift rates and thresholds and $> 70\%$ for relative start points.

It is important to note that in all the model recoveries (as Table S24 illustrates) the estimation of the parameters was quite accurate. That is the order and magnitude of the parameters was maintained. This means the sign of the difference is maintained and the magnitude of the difference is maintained (i.e., a low Type S and Type M error rate) (Gelman & Tuerlinckx, 2000; Gelman & Carlin, 2014). Thus, even if a credible effect may not be found the values of the parameters carry information speaking to the effect. This also means that we could also use a smaller HDI like 90% or even 80%. This would increase the proportion of times a credible effect consistent with the race effect would be identified.

In sum, the hierarchical DDM used in the paper accurately and reliably recovers the generating parameters using simulated datasets that correspond to those used in the literature. It also has reasonable accuracy in detecting medium to large race effects in the process parameters. Nevertheless, we do recommend increasing the number of subjects and the number of trials for smaller effects, and to estimate other interesting aspects of the decision process such as trial-by-trial variability in the parameters.

Table S24: Summary of parameter recovery for three different possible effects of race on the process parameters.

	Race Bias in Start Point				Race Bias in Drift Rate				Race Bias in Drifts & Incr. Thresholds for Black Targets			
	True Value	M	95% HDI		True Value	M	95% HDI		True Value	M	95% HDI	
μ_W^β	0.53	0.54	0.52	0.56	0.55	0.56	0.53	0.58	0.55	0.55	0.53	0.58
μ_B^β	0.58	0.58	0.55	0.60	0.55	0.56	0.53	0.58	0.55	0.56	0.53	0.58
$\sqrt{1/\tau^\beta}$	0.06	0.05	0.04	0.07	0.06	0.05	0.04	0.07	0.06	0.05	0.04	0.07
μ_W^α	1.13	1.14	1.09	1.19	1.13	1.14	1.10	1.19	1.06	1.08	1.03	1.12
μ_B^α	1.13	1.14	1.10	1.19	1.13	1.14	1.10	1.19	1.20	1.21	1.16	1.26
$\sqrt{1/\tau^\alpha}$	0.13	0.14	0.11	0.16	0.13	0.13	0.10	0.16	0.13	0.13	0.11	0.16
$\mu_{NonGun,W}^\delta$	-2.33	-2.32	-2.57	-2.08	-2.71	-2.71	-2.99	-2.43	-2.71	-2.71	-2.99	-2.43
$\mu_{NonGun,B}^\delta$	-2.33	-2.32	-2.57	-2.08	-1.95	-1.96	-2.23	-1.70	-1.95	-1.93	-2.20	-1.68
$\mu_{Gun,W}^\delta$	2.23	2.21	1.97	2.46	1.85	1.86	1.60	2.13	1.85	1.88	1.61	2.15
$\mu_{Gun,B}^\delta$	2.23	2.24	1.99	2.49	2.62	2.61	2.33	2.89	2.62	2.62	2.34	2.90
$\sqrt{1/\tau^\delta}$	0.76	0.76	0.66	0.86	0.76	0.78	0.66	0.90	0.76	0.77	0.67	0.87
$\mu_{NonGun,W}^{NDT}$	0.39	0.39	0.37	0.40	0.39	0.39	0.37	0.40	0.39	0.39	0.37	0.40
$\mu_{NonGun,B}^{NDT}$	0.39	0.39	0.37	0.40	0.39	0.39	0.37	0.40	0.39	0.39	0.37	0.40
$\mu_{Gun,W}^{NDT}$	0.36	0.36	0.34	0.38	0.36	0.36	0.34	0.38	0.36	0.36	0.34	0.38
$\mu_{Gun,B}^{NDT}$	0.36	0.36	0.34	0.38	0.36	0.36	0.34	0.38	0.36	0.36	0.34	0.38
$\sqrt{1/\tau^{NDT}}$	0.06	0.06	0.05	0.06	0.06	0.06	0.05	0.06	0.06	0.06	0.05	0.07

The values for the mean and 95% HDI are averaged across the simulations.

Table S25: Summary of hierarchical DDM ability to identify race effect

	Race Bias in Start Point				Race Bias in Drifts				Race Bias in Drifts & Incr. Thresholds for Black Targets			
	M	95% HDI		Pr(CE)	M	95% HDI		Pr(CE)	M	95% HDI		Pr(CE)
Relative Start Point	0.05	0.02	0.09	.92	0.00	-0.03	0.04	0	0.00	-0.03	0.04	0
Threshold Separation	0.00	-0.07	0.07	0	0.00	-0.07	0.07	0	0.14	0.07	0.21	.98
Drift for non-gun	0.01	-0.37	0.39	0	0.75	0.37	1.13	.98	0.78	0.40	1.16	.98
Drift for gun	0.00	-0.39	0.38	0	0.75	0.36	1.13	.98	0.74	0.36	1.12	1.00
Non-Decision Time	0.00	-0.02	0.02	0	0.00	-0.02	0.02	0	0.00	-0.02	0.02	0

The values for the mean and 95% HDI are averaged across the simulations. The Prop. CE table reports the proportion of simulated datasets where a credible race effect at the group level was found for each process parameter using a 95% HDI.

5 Posterior Predictive Checks for the Drift Diffusion Model

We also examined how well the posterior predictions from the DDM correspond to the observed choice and response time data. For each study and each condition we examined the degree of correspondence between choice probabilities, mean response times, and response time distributions. In terms of the choice probabilities and mean response times we summarized the correspondence between the posterior predictions and the observed data by showing the group-level predictions for each condition and independent response type. We also plotted the observed average performance measure in each plot as well as the average performance measure for each individual. Plotting the distribution of choice proportions and mean response times at the individual level is important as it illustrates that there is shrinkage in posterior predictions for the hierarchical DDM, where lower level parameters are shifted towards the modes of the higher level distributions (Kruschke, 2014). As a result, the posterior predictive HDIs are not centered around the observed average choice proportions and response times from the data, which are more sensitive to extreme values.

We also calculated the proportion of subjects who fall outside the 95% posterior predicted interval for the choice proportions and mean response times in each condition of each study. These are provided in Tables S26, S27, S28, and S29. These statistics are similar to the ones suggested by Gelman et al. (2003) (see Chapter 6) to evaluate lack of fit. They confirm what the figures show, which is the model captures the data well. There are individuals who fall outside the 95% HDI, but by and large it is our judgment that the model does reasonably well in capturing the data across the each condition of each of the four studies. In some cases, particularly in the mean response times for the errors there is some deviation, but this is to be expected given the low numbers of observations at the subject level.

The posterior predictions of the mean response times in Studies 1 and 2 also reveal the consequences of the response deadlines. All the observed data are, by design, before the response deadline. Normally, this would be a problem for the DDM, as it predicts a response time distribution that is continuous over all possible times. The Bayesian approach taken in this paper, however, makes it possible to model this censoring of the data and, as a result, the posterior predictions do a good job of recreating the data, even with the cutoff (see Section 2.2). However, one noticeable exception is seen in the mean response times for the "Don't Shoot" responses to non-gun objects in Study 2 (see Figure S6). The aggregate response time distributions in Figure S7 show that these responses (which tend to be the slowest) were affected by the response window (630 ms) in this study cutting off the right tail of the distribution leading to the poor fits. As this problem was only evident in this study we left the DDM as is and did not work to correct this misfit.

As just mentioned, we also also examined the degree of correspondence between the observed response time distributions and the predicted response time distributions. To generate the predicted response times we used JAGS to sample from the DDM using the posterior group level distributions. For each condition and response, we then collapsed across subjects and trials to estimate the predicted response time distribution. Note this creates an incredibly large amount of samples of response times (e.g., 80,000 samples per trial) so we randomly sample a smaller sample of samples per trial (e.g., 8000 per trial). Per the recommendation of Van Zandt (2000), to plot the response time distributions we passed the observed response times and predicted response times through a Gaussian kernel.

All in all given the different studies, each with multiple conditions, with many subjects, we believe the fit of the model to the data is reasonably good. There are certainly places for improvement, but we leave those for studies better designed to investigate more fine-tuned models.

5.1 Study 1

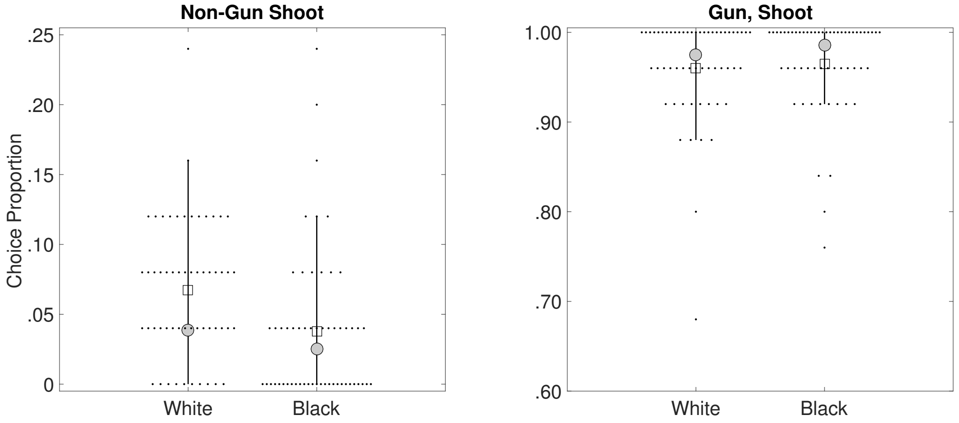

Figure S2: Posterior predictions of the false alarm and hit rates in Study 1. The large shaded dots are the mean posterior prediction at the group level. The bars are the 95% HDI. The unshaded squares are the observed average false alarm and hit rates. The small solid dots are the observed individual rates, which are scattered horizontally to better show the distribution of individual rates. All values were normalized to 25 observations each, the number of trials in each condition.

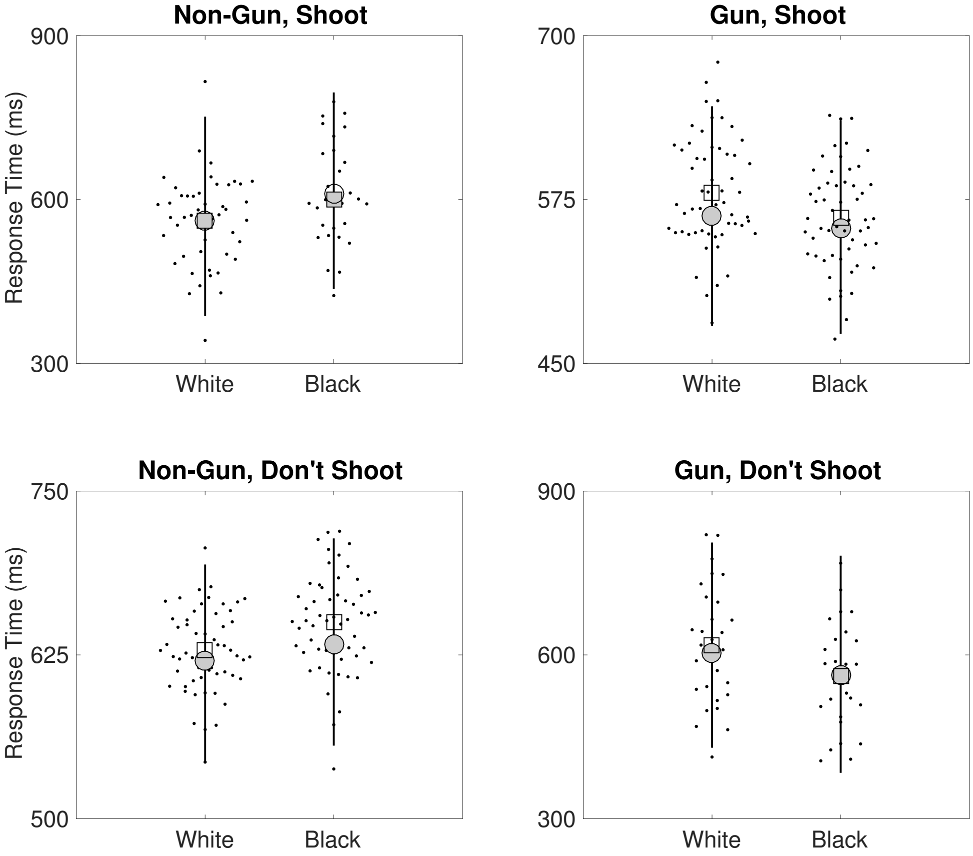

Figure S3: Posterior predictions of the mean response times in Study 1. The large shaded dots are the mean posterior prediction at the group level. The bars are the 95% HDI. The unshaded squares are the observed average response times. The small solid dots are the observed average response times, which are scattered horizontally to better show the distribution of individual response times.

Table S26: Proportion of observed choice proportions and mean response times falling outside the posterior predicted 95%HDI in Study 1 (see Figures S2 and S3).

	Choice Proportions	Response Times	
		Don't Shoot	Shoot
White, Non-Gun	.02	.02	.04
Black, Non-Gun	.07	.05	.02
White, Gun	.07	.05	.07
Black, Gun	.07	0	.07

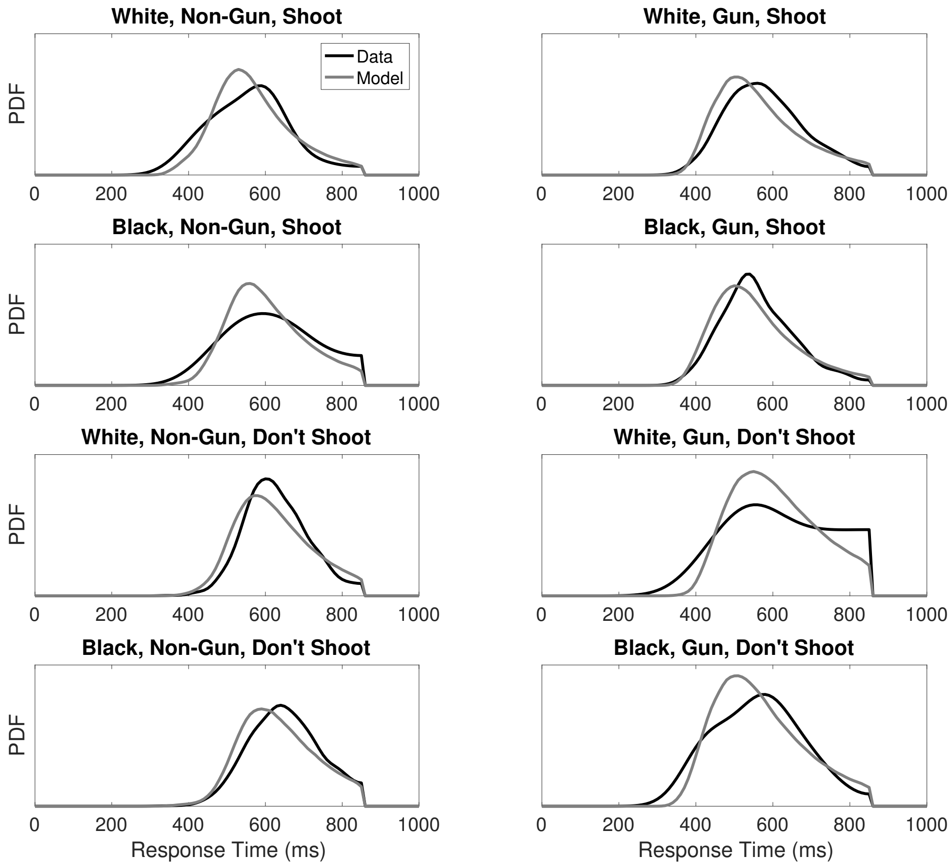

Figure S4: Observed (black) and predicted (grey) response time distributions at the group level for Study 1. The response window was 850 ms and all responses outside this window were not recorded.

5.2 Study 2

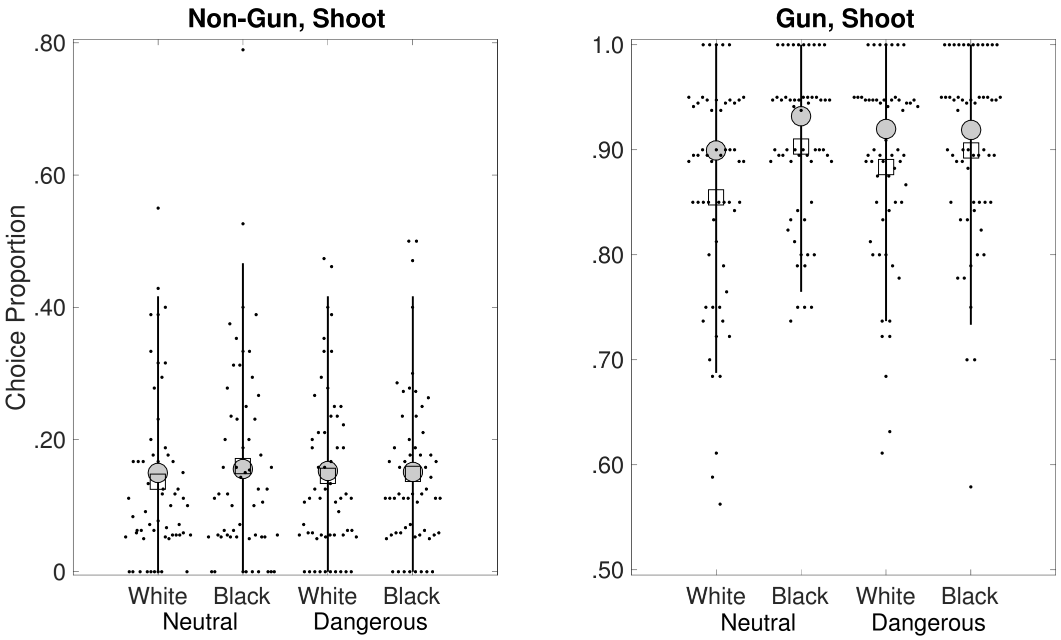

Figure S5: Posterior predictions of the false alarm and hit rates in Study 2. The large shaded dots are the mean posterior prediction at the group level. The bars are the 95% HDI. The unshaded squares are the observed average false alarm and hit rates. The small solid dots are the observed individual rates, which are scattered horizontally to better show the distribution of individual rates. All values were normalized to 20 observations each, the number of trials in each condition.

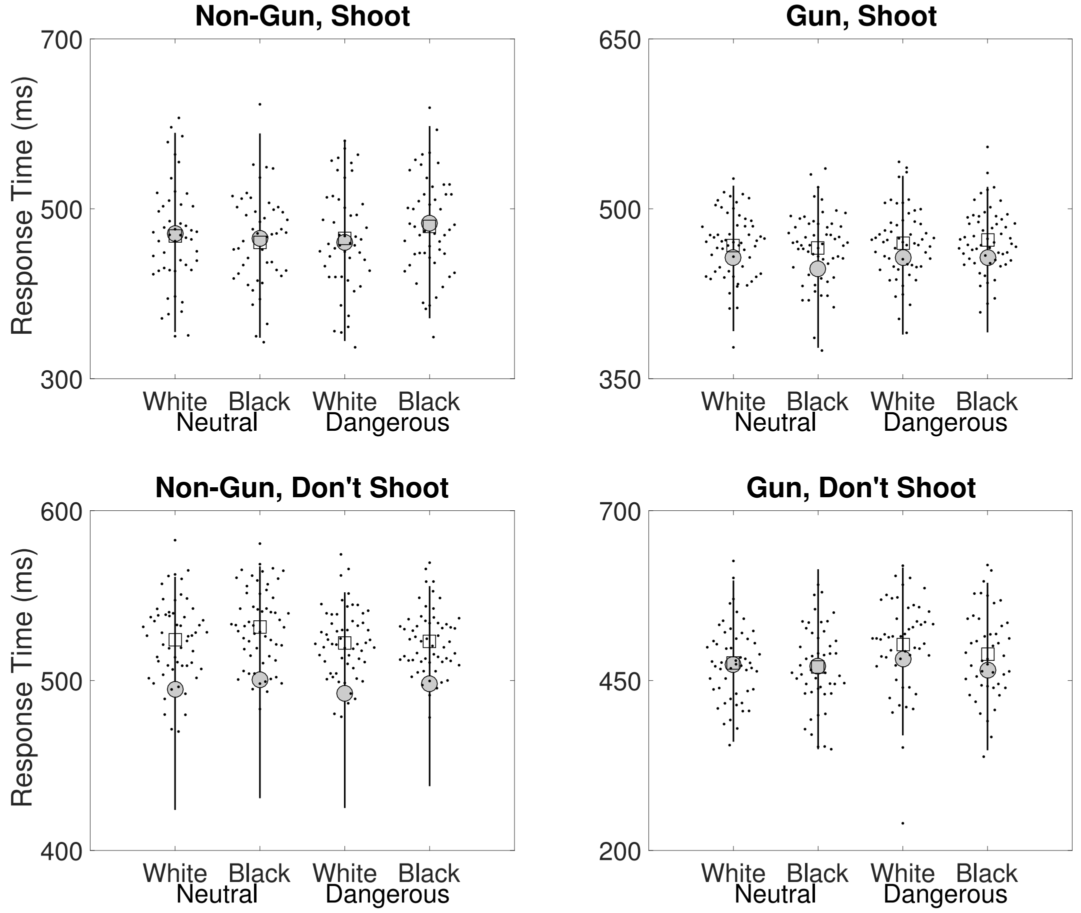

Figure S6: Posterior predictions of the mean response times in Study 2. The large shaded dots are the mean posterior prediction at the group level. The bars are the 95% HDI. The unshaded squares are the observed average response times. The small solid dots are the observed average response times, which are scattered horizontally to better show the distribution of individual response times. Note that the model systematically predicts faster response times in correct rejections (non-gun, "Don't Shoot"). As Figure S7 shows this appears to occur because the response window disproportionately impacts non-gun objects (the slower responses), which is especially apparent in this study with the smallest response window (630 ms). As a result the DDM struggles to account for the distribution of response times for the non-gun objects. Given this misfit does not appear to happen in the other responses or in the other studies we have chosen to leave the model as is.

Table S27: Proportion of observed choice proportions and mean response times falling outside the posterior predicted 95% HDI in Study 2 (see Figures S5 and S6).

	Choice Proportions	Response Times	
		Don't Shoot	Shoot
White, Non-Gun, Neutral	.03	.07	.07
Black, Non-Gun, Neutral	.03	.03	.05
White, Gun, Neutral	.05	.05	.03
Black, Gun, Neutral	0	0	.07
White, Non-Gun, Danger	.03	.05	.02
Black, Non-Gun, Danger	.05	.07	.03
White, Gun, Danger	.09	.05	.05
Black, Gun, Danger	.05	.07	.03

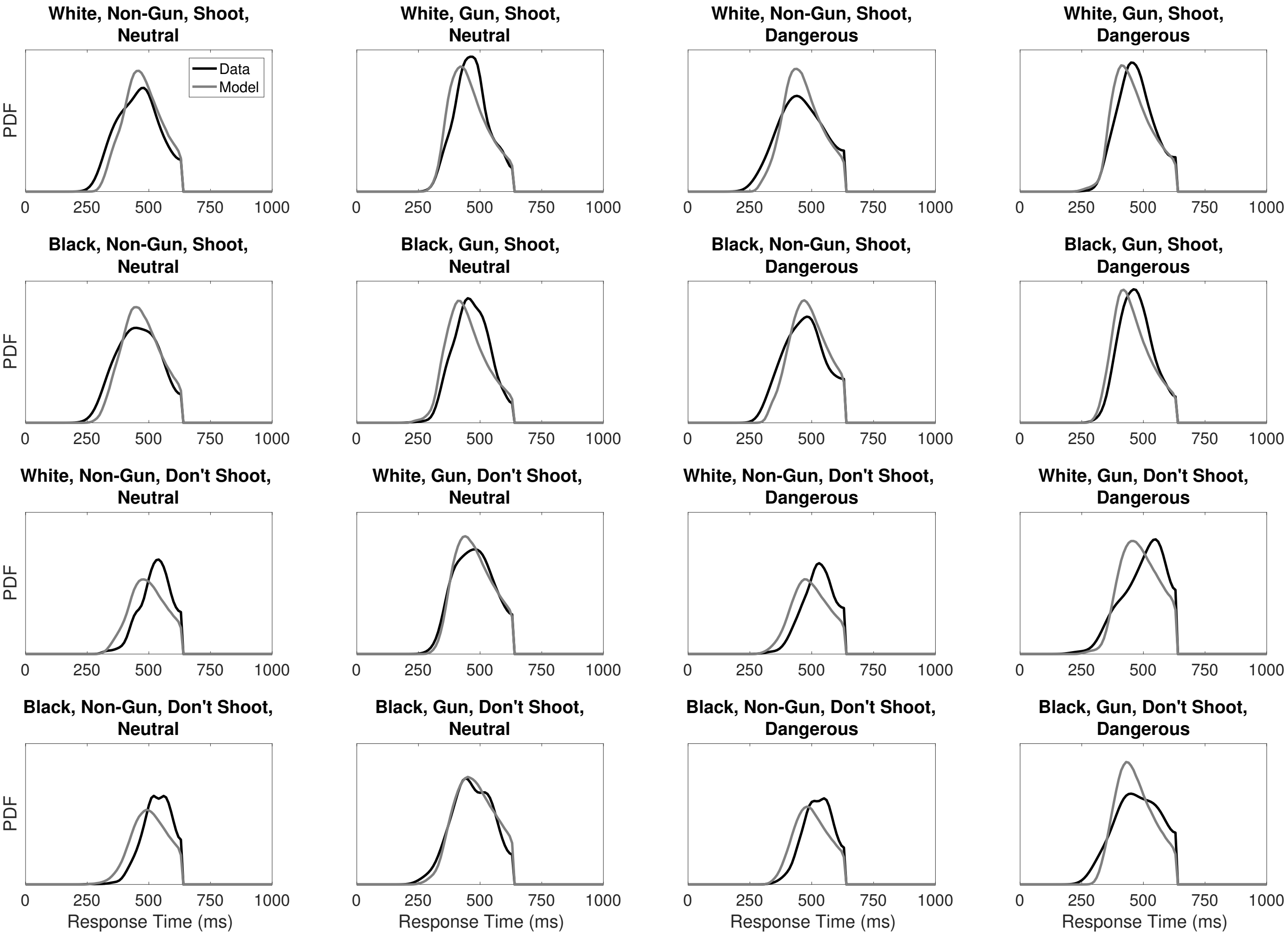

Figure S7: Observed (black) and predicted (grey) response time distributions at the group level for Study 2. The response window was 630 ms and all responses beyond this window were not recorded in this study. Referring back to Figure S6 note the response window tended to have a larger impact on the non-gun response time distributions, particularly for the “Don’t Shoot” response.

5.3 Study 3

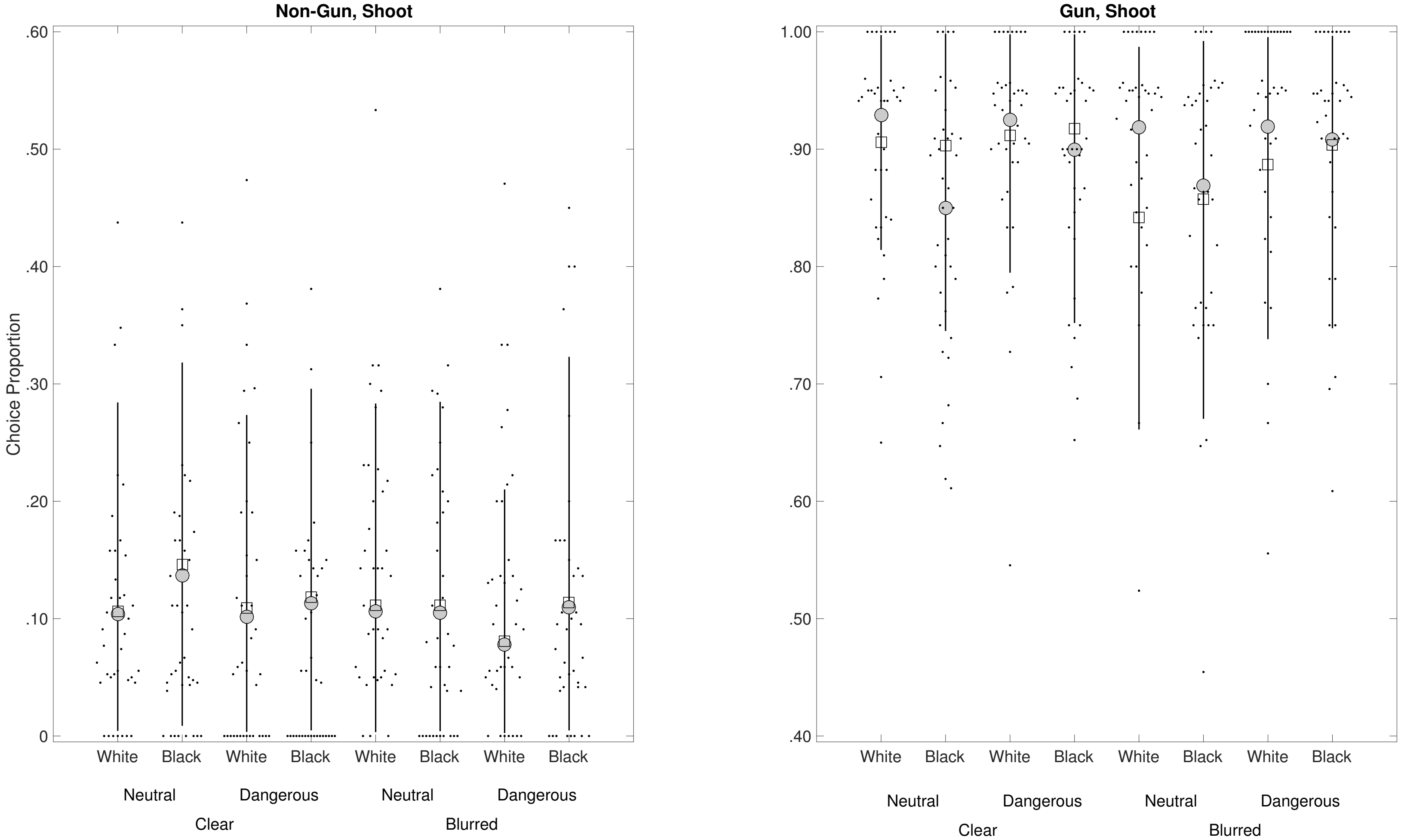

Figure S8: Posterior predictions of the false alarm and hit rates in Study 3. The large shaded dots are the mean posterior prediction at the group level. The bars are the 95% HDI. The unshaded squares are the observed average false alarm and hit rates. The small solid dots are the observed individual rates, which are scattered horizontally to better show the distribution of individual rates. Note that the predicted hit rates appear more regressive to the average hit rate across conditions than the observed hit rates. This is due to shrinkage in the hierarchical model. All values were normalized to 20 observations each, the number of trials in each condition.

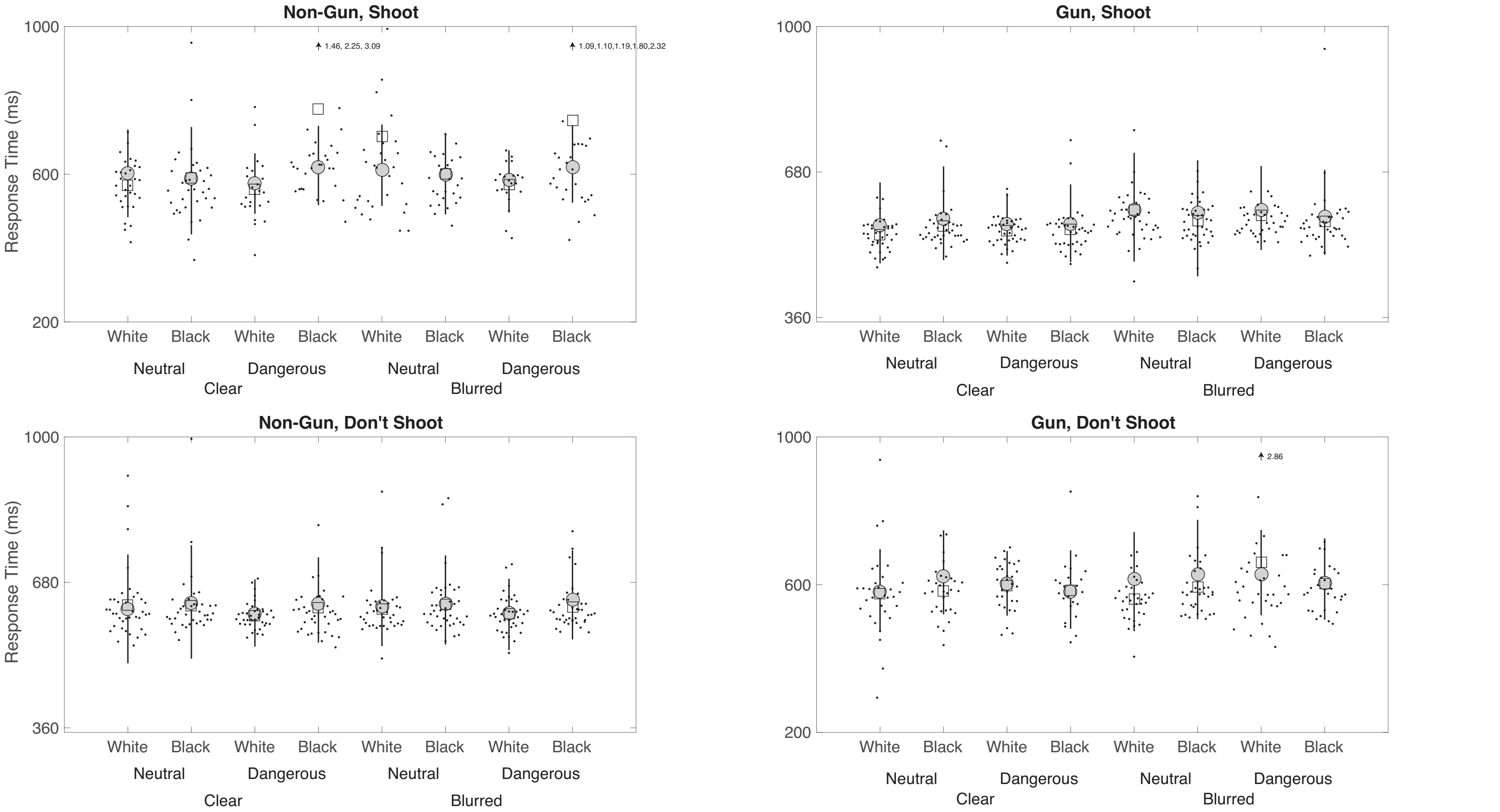

Figure S9: Posterior predictions of the mean response times in Study 3. The large shaded dots are the mean posterior prediction at the group level. The bars are the 95% HDI. The unshaded squares are the observed average response times. The small solid dots are the observed average response times, which are scattered horizontally to better show the distribution of individual response times.

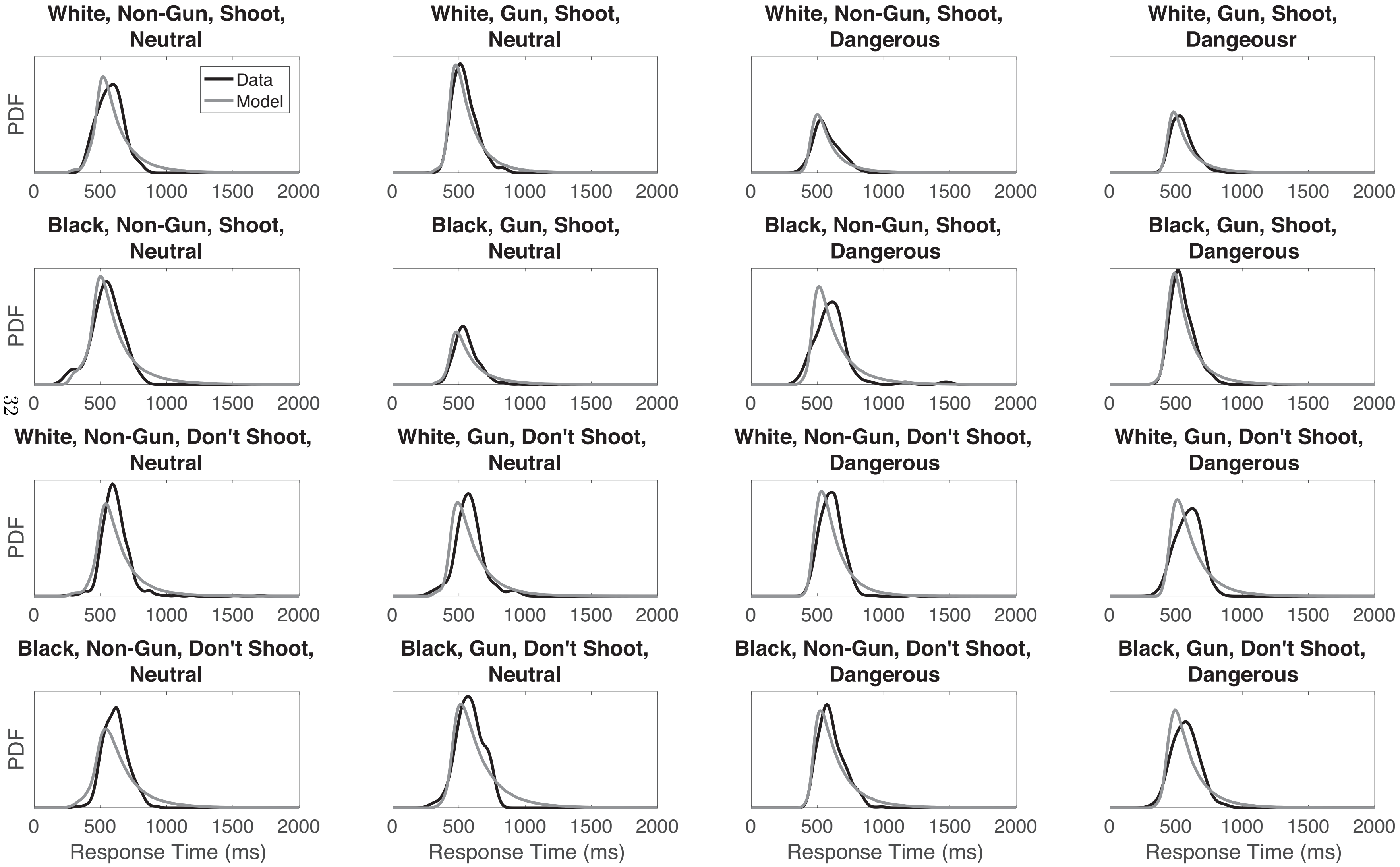

Figure S10: Observed (black) and predicted (grey) response time distributions at the group level for Study 3 in the clear condition.

Table S28: Proportion of observed choice proportions and mean response times falling outside the posterior predicted 95%HDI in Study 3 (see Figures S8 and S9).

	Choice Proportions	Response Times	
		Don't Shoot	Shoot
White, Non-Gun, Neutral, Clear	.08	.11	.11
Black, Non-Gun, Neutral, Clear	.08	.06	.16
White, Gun, Neutral, Clear	.11	.21	.03
Black, Gun, Neutral, Clear	.26	.13	.05
White, Non-Gun, Danger, Clear	.16	.03	.16
Black, Non-Gun, Danger, Clear	.08	.08	.13
White, Gun, Danger, Clear	.11	.13	.05
Black, Gun, Danger, Clear	.08	.08	.08
White, Non-Gun, Neutral, Blur	.08	.08	.32
Black, Non-Gun, Neutral, Blur	.05	.08	.08
White, Gun, Neutral, Blur	.05	.11	.08
Black, Gun, Neutral, Blur	.08	.08	0
White, Non-Gun, Danger, Blur	.08	.08	.08
Black, Non-Gun, Danger, Blur	.11	.05	.24
White, Gun, Danger, Blur	.08	.21	.03
Black, Gun, Danger, Blur	.08	.08	.05

Note the discrepancies between observed and the posterior predicted RTs tend to be greater in the errors, which are the responses with the fewest number of observations.

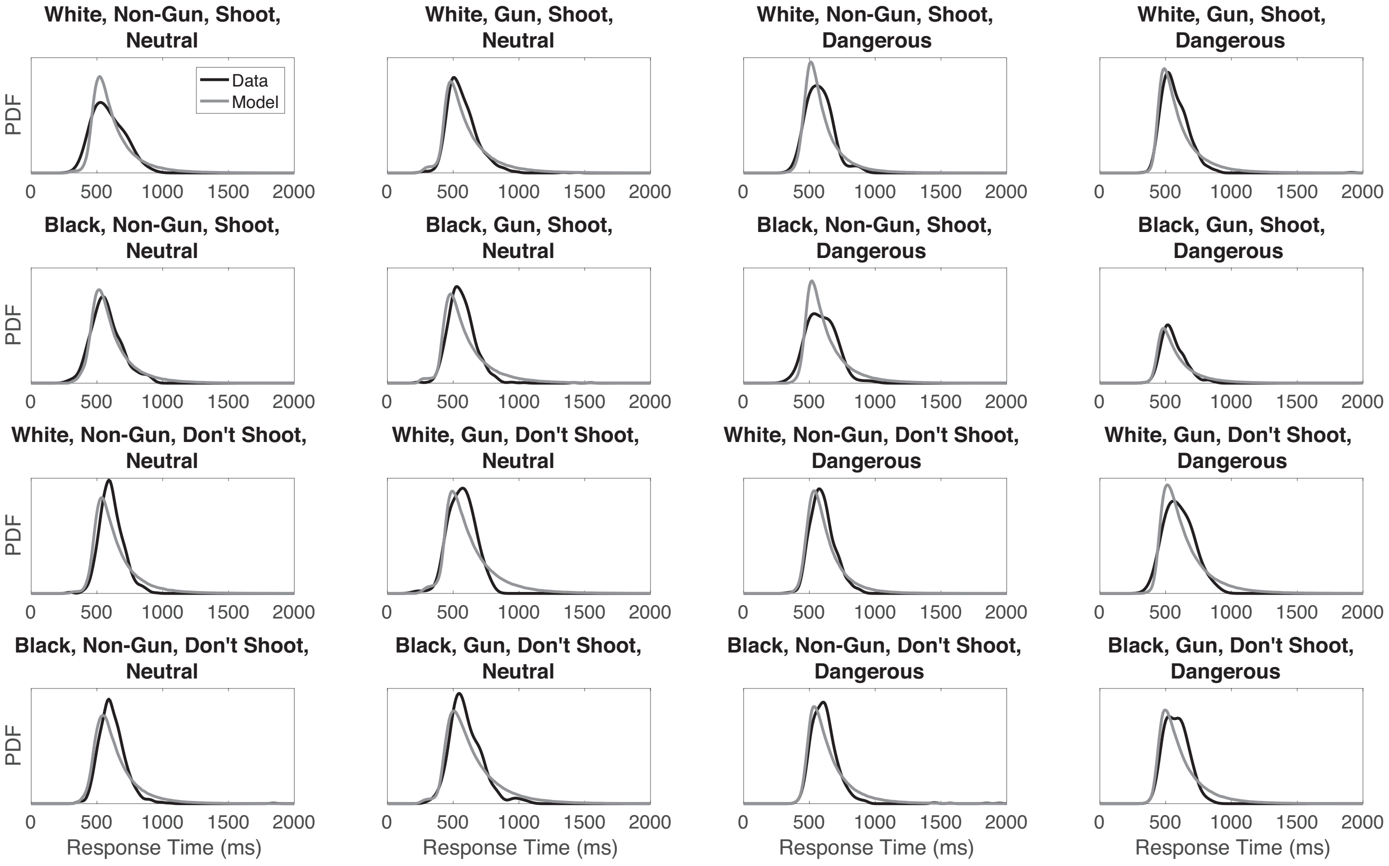

Figure S11: Observed (black) and predicted (grey) response time distributions at the group level for Study 3 in the blurred condition.

5.4 Study 4

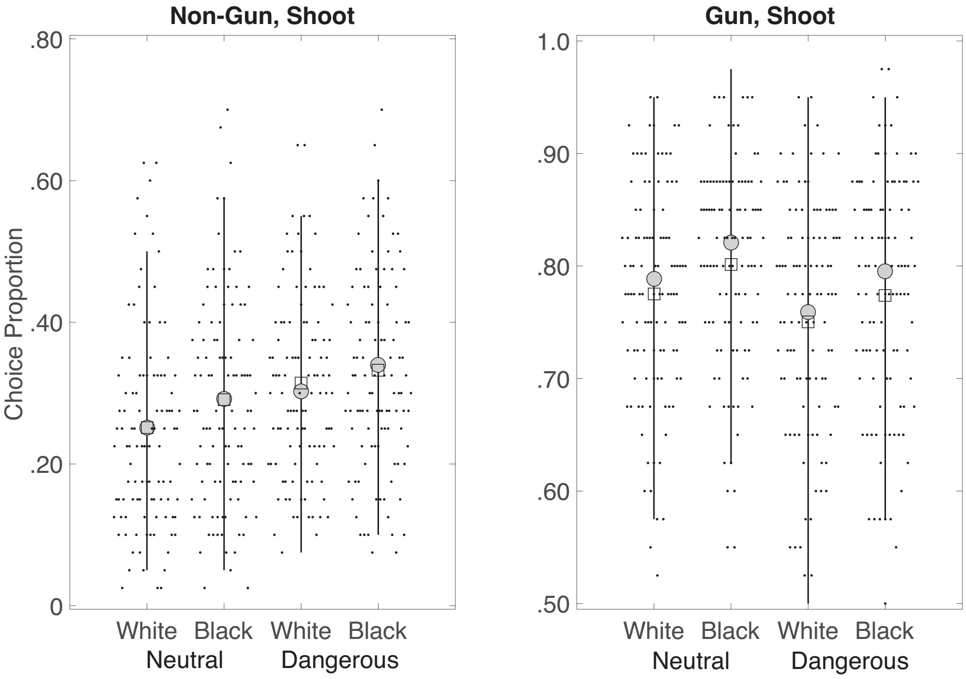

Figure S12: Posterior predictions of the false alarm and hit rates in Study 4. The large shaded dots are the mean posterior prediction at the group level. The bars are the 95% HDI. The unshaded squares are the observed average false alarm and hit rates. The small solid dots are the observed individual rates, which are scattered horizontally to better show the distribution of individual rates. All values were normalized to 40 observations each, the number of trials in each condition. Note that the predicted hit rates appear more regressive to the average hit rate across conditions than the observed hit rates. This is due to shrinkage in the hierarchical model.

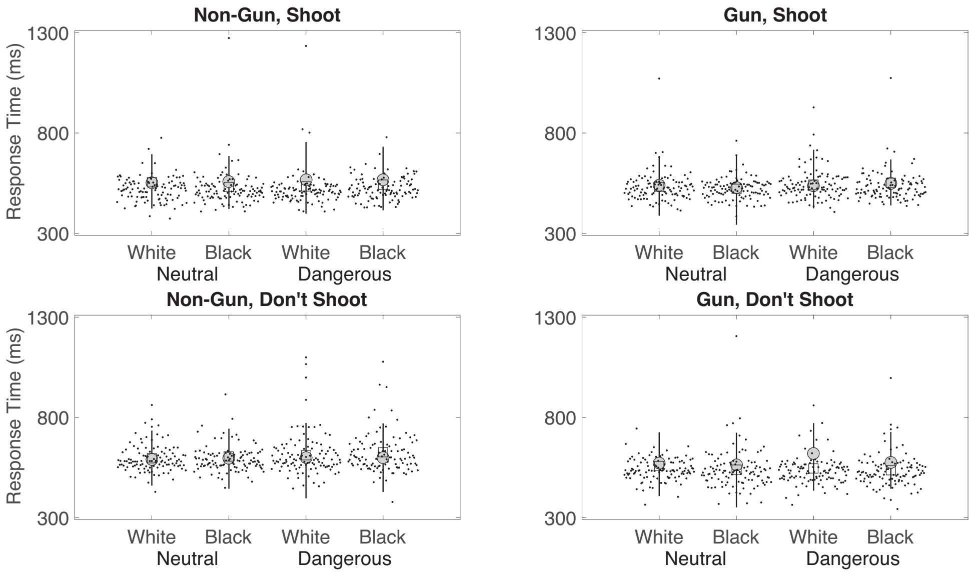

Figure S13: Posterior predictions of the mean response times in Study 4. The large shaded dots are the mean posterior prediction at the group level. The bars are the 95% HDI. The unshaded squares are the observed average response times. The small solid dots are the observed average response times, which are scattered horizontally to better show the distribution of individual response times.

Table S29: Proportion of observed choice proportions and mean response times falling outside the posterior predicted 95%HDI in Study 4 (see Figures S12 and S13).

	Choice Proportions	Response Times	
		Don't Shoot	Shoot
White, Non-Gun, Neutral	.10	.05	.08
Black, Non-Gun, Neutral	.04	.05	.08
White, Gun, Neutral	.06	.04	.07
Black, Gun, Neutral	.05	.10	.05
White, Non-Gun, Danger	.02	.05	.03
Black, Non-Gun, Danger	.03	.06	.02
White, Gun, Danger	.05	.10	.06
Black, Gun, Danger	.05	.15	.07

Note the discrepancies between observed and the posterior predicted RTs tend to be greater in the errors, which are the responses with the fewest number of observations.

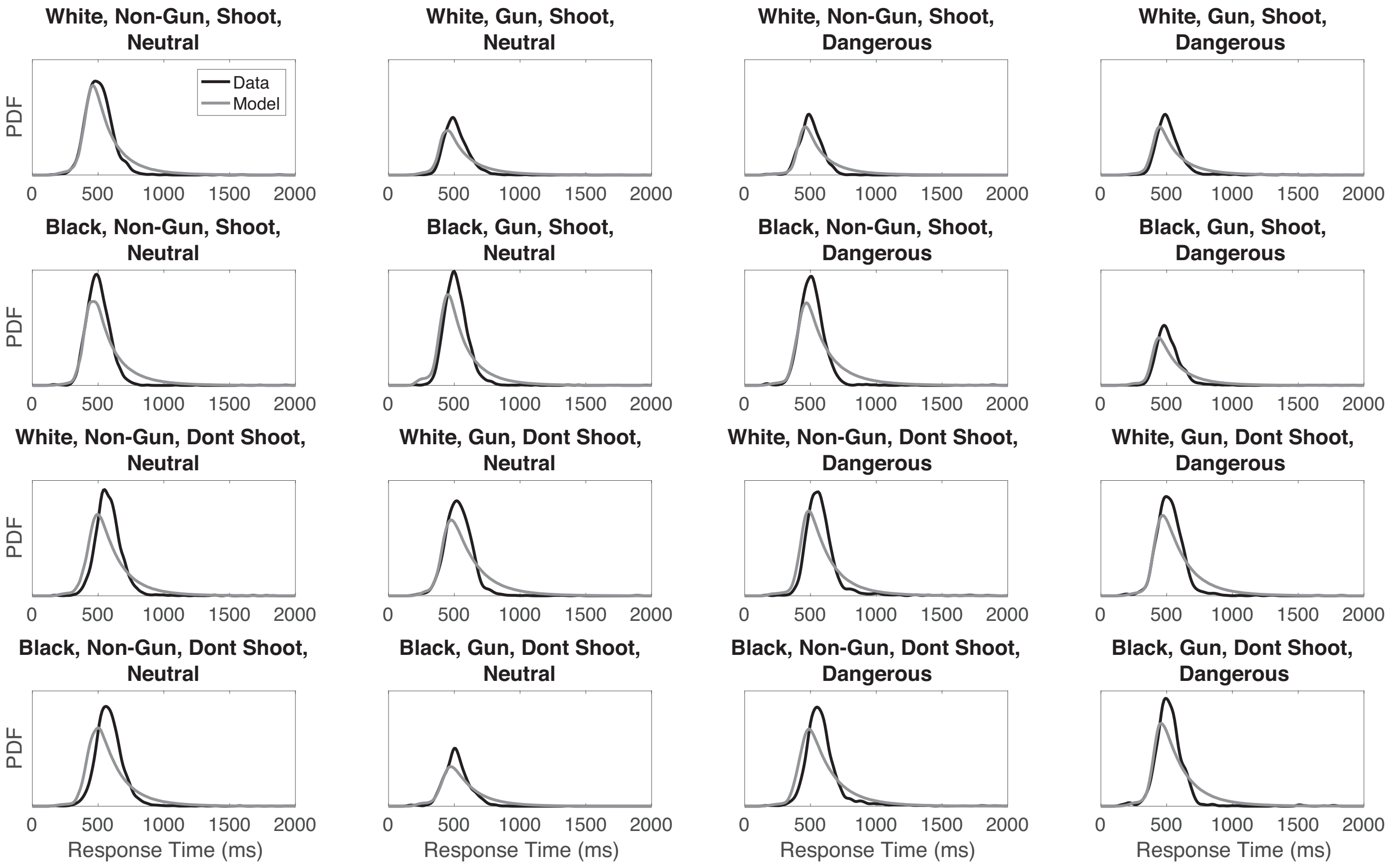

Figure S14: Observed (black) and predicted (grey) response time distributions at the group level for Study 4 in the clear condition. The correspondence between the posterior predicted response time distributions and observed response times is lower in this study. The observed data have a more symmetrical distribution. One way to account for this is to include trial-by-trial variability in the start point. However, we leave this investigation of trial-by-trial variability in the parameters to a study with more observations per subject.

6 Drift Diffusion Model Process Parameter Effects Tables

This appendix summarizes the effects on the group level process parameters in the DDM for Studies 2, 3, 4 and the composite analysis.

6.1 Study 2

Table S30: Summary of Standardized Effects of Race and Context on Group Level μ^{β} for Study 2

		95% HDI		
Factor	Mean	Lower	Upper	Prop <0
Race	-0.159	-0.546	0.223	.792
Context	-0.094	-0.479	0.292	.061
Race * Context	0.418	0.024	0.807	.476

Table S31: Summary of Standardized Effects of Race and Context on Group Level μ^{α} for Study 2

		95% HDI		
Factor	Mean	Lower	Upper	Prop <0
Race	0.250	-0.204	0.728	.142
Context	-0.071	-0.548	0.391	.616
Race * Context	0.573	0.088	1.084	.008

Table S32: Summary of Standardized Effects of Race and Context on Group Level μ^{δ} for Non-Gun Objects in Study 2

		95% HDI		
Factor	Mean	Lower	Upper	Prop <0
Race	0.043	-0.308	0.379	.405
Context	0.087	-0.260	0.438	.313
Race * Context	0.050	-0.293	0.405	.389

Table S33: Summary of Standardized Effects of Race and Context on Group Level μ^{δ} for Gun Objects in Study 2

		95% HDI		
Factor	Mean	Lower	Upper	Prop <0
Race	0.432	0.077	0.788	.005
Context	0.047	-0.313	0.401	.400
Race * Context	0.141	-0.213	0.497	.219

6.2 Study 3

Table S34: Summary of Standardized Effects of Race and Context on Group Level μ^{NDT} for Study 2

		95% HDI		
Factor	Mean	Lower	Upper	Prop <0
Race	0.008	-0.185	0.202	.468
Object	-0.646	-0.842	-0.450	1.00
Context	0.041	-0.156	0.234	.340
Race * Object	0.169	-0.025	0.357	.043
Race * Context	-0.205	-0.400	-0.011	.981
Object * Context	-0.077	-0.269	0.114	.786
Race * Object * Context	-0.084	-0.276	0.104	.808

Table S35: Summary of Standardized Effects of Race and Context on Group Level μ^{β} for Study 3

		95% HDI		
Factor	Mean	Lower	Upper	Prop <0
Race	0.047	-0.447	0.545	.424
Context	-0.027	-0.532	0.470	.542
Blur	-0.166	-0.662	0.327	.747
Race * Context	-0.712	-1.259	-0.188	.997
Race * Blur	0.096	-0.401	0.593	.347
Context * Blur	0.109	-0.389	0.602	.333
Race * Context * Blur	0.228	-0.286	0.731	.184

Table S36: Summary of Standardized Effects of Race and Context on Group Level μ^{α} for Study 3

		95% HDI		
Factor	Mean	Lower	Upper	Prop <0
Race	0.325	-0.683	0.683	.036
Context	-0.248	-0.103	-0.103	.915
Blur	0.036	-0.396	0.396	.420
Race * Context	-0.007	-0.348	0.348	.520
Race * Blur	-0.078	-0.283	0.283	.671
Context * Blur	0.149	-0.498	0.498	.205
Race * Context * Blur	-0.066	-0.282	0.282	.645

Table S37: Summary of Standardized Effects of Race and Context on Group Level μ^{δ} for Non-Gun Objects in Study 3

		95% HDI		
Factor	Mean	Lower	Upper	Prop <0
Race	0.342	0.047	0.643	.012
Context	-0.241	-0.535	-0.056	.944
Blur	-0.158	-0.461	0.132	.851
Race * Context	0.272	-0.018	0.572	.035
Race * Blur	-0.096	-0.391	0.197	.737
Context * Blur	-0.007	-0.306	0.290	.516
Race * Context * Blur	0.097	-0.203	0.388	.262

Table S38: Summary of Standardized Effects of Race and Context on Group Level μ^{δ} for Gun Objects in Study 3

		95% HDI		
Factor	Mean	Lower	Upper	Prop <0
Race	0.075	-0.228	0.376	.312
Context	0.420	0.117	0.721	.004
Blur	-0.624	-0.98	-0.318	.999
Race * Context	0.217	-0.083	0.522	.078
Race * Blur	0.104	-0.188	0.409	.247
Context * Blur	0.219	-0.743	0.525	.076
Race * Context * Blur	-0.067	-0.361	0.236	.672

6.3 Study 4

Table S39: Summary of Standardized Effects of Race and Context on Group Level μ^{NDT} for Study 3

		95% HDI		
Factor	Mean	Lower	Upper	Prop <0
Race	-0.153	-0.326	0.021	.959
Context	0.353	0.179	0.526	<.001
Blur	0.085	-0.087	0.255	.165
Object	-0.615	-0.791	-0.442	1.00
Race * Context	-0.015	-0.185	0.158	.568
Race * Blur	0.046	-0.127	0.216	.301
Race * Object	-0.008	-0.178	0.161	.539
Context * Blur	0.007	-0.164	0.178	.469
Context * Object	-0.042	-0.211	0.128	.688
Blur * Object	0.173	-0.000	0.340	.023
Race * Context * Blur	0.026	-0.145	0.197	.382
Object * Race * Context	0.167	-0.002	0.345	.028
Object * Race * Blur	0.083	-0.092	0.249	.172
Object * Context * Blur	-0.142	-0.310	0.030	.949
Race * Object * Context * Blur	-0.052	-0.223	0.116	.726

Table S40: Summary of Standardized Effects of Race and Context on Group Level μ^{β} for Study 4

		95% HDI		
Factor	Mean	Lower	Upper	Prop <0
Race	-0.244	-0.502	0.015	.969
Context	-0.206	-0.467	0.044	.944
Race * Context	0.044	-0.209	0.304	.368

Table S41: Summary of Standardized Effects of Race and Context on Group Level μ^{α} for Study 4

		95% HDI		
Factor	Mean	Lower	Upper	Prop <0
Race	0.068	-0.135	0.270	.255
Context	-0.105	-0.304	0.100	.845
Race * Context	-0.125	0.324	0.0822	.886

Table S42: Summary of Standardized Effects of Race and Context on Group Level μ^{δ} for Non-Gun Objects in Study 4

		95% HDI		
Factor	Mean	Lower	Upper	Prop <0
Race	0.311	0.088	0.530	.003
Context	0.475	0.255	0.696	< .001
Race * Context	0.012	-0.210	0.228	.456

Table S43: Summary of Effects of Race and Context on Group Level μ^{δ} for Gun Objects in Study 4

		95% HDI		
Factor	Mean	Lower	Upper	Prop <0
Race	0.326	0.102	0.553	.002
Context	-0.205	-0.433	0.016	.964
Race * Context	0.001	-0.212	0.235	.475

Table S44: Summary of Standardized Effects of Race and Context on Group Level μ^{NDT} for Study 4

		95% HDI		
Factor	Mean	Lower	Upper	Prop <0
Race	-0.064	-0.196	0.074	.827
Object	-0.194	-0.331	-0.062	.998
Context	0.008	-0.127	0.142	.453
Race * Object	-0.019	-0.154	0.115	.607
Race * Context	-0.037	-0.174	0.096	.704
Object * Context	0.014	-0.121	0.148	.419
Race * Object * Context	0.006	-0.126	0.143	.468

6.4 Composite Analysis

Table S45: Summary of Standardized Effects of Race and Context on Group Level μ^{β} for Composite Analysis

		95% HDI		
Factor	Mean	Lower	Upper	Prop <0
Race	0.007	-0.310	0.296	.519
Window	-0.131	-0.436	0.172	.803
Race * Window	-0.125	-0.425	0.181	.792

Table S46: Summary of Standardized Effects of Race and Context on Group Level μ^{α} for Composite Analysis

		95% HDI		
Factor	Mean	Lower	Upper	Prop <0
Race	0.315	0.047	0.576	.010
Window	1.64	1.350	1.939	0
Race * Window	0.159	-0.099	0.421	.115

Table S47: Summary of Standardized Effects of Race and Context on Group Level μ^{δ} for Non-Gun Objects in Composite Analysis

		95% HDI		
Factor	Mean	Lower	Upper	Prop <0
Race	0.171	-0.075	0.421	.087
Window	-1.18	-1.440	-0.926	1
Race * Window	0.041	-0.204	0.293	.374

Table S48: Summary of Effects of Race and Context on Group Level μ^{δ} for Gun Objects in Composite Analysis

		95% HDI		
Factor	Mean	Lower	Upper	Prop <0
Race	0.254	0.008	0.511	.023
Window	1.23	0.980	1.502	0
Race * Window	0.028	-0.218	0.285	.415

Table S49: Summary of Standardized Effects of Race and Context on Group Level μ^{NDT} for Composite Analysis

		95% HDI		
Factor	Mean	Lower	Upper	Prop <0
Race	-0.083	-0.221	0.051	.885
Window	0.921	0.780	1.063	0
Object	-0.470	-0.608	-0.335	1
Race * Window	-0.002	-0.136	0.135	.510
Race * Object	0.039	-0.099	0.171	.284
Window * Object	-0.002	-0.136	0.135	.510
Race * Window * Object	-0.001	-0.133	0.136	.509

References

Correll, J., Park, B., Judd, C. M., & Wittenbrink, B. (2002). The police officer's dilemma: Using ethnicity to disambiguate potentially threatening individuals. *Journal of Personality and Social Psychology*, *83*(6), 1314–1329. doi: 10.1037/0022-3514.83.6.1314

Correll, J., Park, B., Judd, C. M., & Wittenbrink, B. (2007). The influence of stereotypes on decisions to shoot. *European Journal of Social Psychology*, *37*(6), 1102–1117. doi: 10.1002/ejsp.450

Correll, J., Park, B., Judd, C. M., Wittenbrink, B., Sadler, M. S., & Keesee, T. (2007). Across the thin blue line: Police officers and racial bias in the decision to shoot. *Journal of Personality and Social Psychology*, *92*(6), 1006–1023. doi: 10.1037/0022-3514.92.6.1006

Correll, J., Wittenbrink, B., Park, B., Judd, C. M., & Goyle, A. (2011). Dangerous enough: Moderating racial bias with contextual threat cues. *Journal of Experimental Social Psychology*, *47*(1), 184–189. doi: 10.1016/j.jesp.2010.08.017

Gelman, A., & Carlin, J. (2014). Beyond power calculations assessing type s (sign) and type m (magnitude) errors. *Perspectives on Psychological Science*, *9*(6), 641–651. doi: 10.1177/1745691614551642

Gelman, A., Carlin, J. B., Stern, H. S., & Rubin, D. B. (2003). *Bayesian data analysis, second edition.* New York, Ny: Chapman & Hall/CRC.

Gelman, A., & Tuerlinckx, F. (2000). Type s error rates for classical and bayesian single and multiple comparison procedures. *Computational Statistics*, *15*(3), 373–390.

Greenwald, A. G., Oakes, M. A., & Hoffman, H. G. (2003). Targets of discrimination: Effects of race on responses to weapons holders. *Journal of Experimental Social Psychology*, *39*(4), 399–405. doi: 10.1016/S0022-1031(03)00020-9

Heathcote, A., Brown, S. D., & Wagenmakers, E.-J. (2015). An introduction to good practices in cognitive modeling. In B. Forstmann & E. J. Wagenmakers (Eds.), *An introduction to model-based cognitive neuroscience* (pp. 25–48). Springer.

JASP Team. (2017). *JASP (version 0.8.1)[computer software].* Retrieved from `https://jasp-stats.org`

Kenworthy, J. B., Barden, M. A., Diamond, S., & del Carmen, A. (2011). Ingroup identification as a moderator of racial bias in a shoot–no shoot decision task. *Group Processes & Intergroup Relations*, *14*(3), 311–318. doi: 10.1177/1368430210392932

Kruschke, J. K. (2010). What to believe: Bayesian methods for data analysis. *Trends in Cognitive Sciences*, *14*(7), 293–300. doi: 10.1016/J.Tics.2010.05.001

Kruschke, J. K. (2013). Bayesian estimation supersedes the t test. *Journal of Experimental Psychology: General*, *142*(2), 573–603. doi: 10.1037/a0029146

Kruschke, J. K. (2014). *Doing Bayesian data analysis: A tutorial with R, JAGS, and Stan* (2nd ed.). New York, NY: Academic Press/Elsevier.

Lee, M. D. (2008). BayesSDT: Software for Bayesian inference with signal detection theory. *Behavior Research Methods*, *40*(2), 450–456. doi: 10.3758/BRM.40.2.450

Lee, M. D., & Wagenmakers, E. J. (2013). *Bayesian modeling for cognitive science: A practical course.* New York, NY: Cambridge University Press.

Ma, D. S., Correll, J., Wittenbrink, B., Bar-Anan, Y., Sriram, N., & Nosek, B. A. (2013). When fatigue turns deadly: The association between fatigue and racial bias in the decision to shoot. *Basic and Applied Social Psychology*, *35*(6), 515–524. doi: 10.1080/01973533.2013.840630

Morey, R. D., & Rouder, J. N. (2015). *BayesFactor (version 0.9.10-2).* Retrieved from `https://jasp-stats.org`

Raftery, A. E. (1995). Bayesian model selection in social research. In P. V. Marsden (Ed.), *Sociological methodology 1995* (pp. 111–196). Cambridge, MA: Blackwell.

Ratcliff, R. (1978). A theory of memory retrieval. *Psychological Review*, *85*(2), 59–108. doi: 10.1037/0033-295X.85.2.59

Ratcliff, R., & Rouder, J. N. (1998). Modeling response times for two-choice decisions. *Psychological Science*, *9*(5), 347–356. doi: 10.1111/1467-9280.00067

Ratcliff, R., & Smith, P. L. (2004). A comparison of sequential sampling models for two-choice reaction time. *Psychological Review*, *111*(2), 333–367. doi: 10.1037/0033-295x.111.2.333

Rouder, J. N., Morey, R. D., Verhagen, J., Swagman, A. R., & Wagenmakers, E.-J. (2016). Bayesian analysis of factorial designs. *Psychological Methods*(Advance online publication). doi: 10.1037/met0000057

Sadler, M. S., Correll, J., Park, B., & Judd, C. M. (2012). The world is not black and white: Racial bias in the decision to shoot in a multiethnic context. *Journal of Social Issues*, *68*(2), 286–313. doi: 10.1111/j.1540-4560.2012.01749.x

Sim, J. J., Correll, J., & Sadler, M. S. (2013). Understanding police and expert performance: When training attenuates (vs. exacerbates) stereotypic bias in the decision to shoot. *Personality and Social Psychology Bulletin*, *39*(3), 291–304. doi: 10.1177/0146167212473157

Tuerlinckx, F., Maris, E., Ratcliff, R., & De Boeck, P. (2001). A comparison of four methods for simulating the diffusion process. *Behavior Research Methods, Instruments, & Computers*, *33*(4), 443–456. doi: 10.3758/BF03195402

Vandekerckhove, J., Tuerlinckx, F., & Lee, M. D. (2011). Hierarchical diffusion models for two-choice response times. *Psychological Methods*, *16*(1), 44–62. doi: 10.1037/a0021765

Van Zandt, T. (2000). How to fit a response time distribution. *Psychonomic Bulletin & Review*, *7*(3), 424–465. doi: 10.3758/BF03214357

Wabersich, D., & Vandekerckhove, J. (2014). Extending JAGS: A tutorial on adding custom distributions to JAGS (with a diffusion model example). *Behavior Research Methods*, *46*(1), 15–28. doi: 10.3758/s13428-013-0369-3
